# Utilization of the evidence from studies with no events in meta-analyses of adverse events: an empirical investigation

**DOI:** 10.1186/s12916-021-02008-2

**Published:** 2021-06-15

**Authors:** Chang Xu, Xiaoqin Zhou, Liliane Zorzela, Ke Ju, Luis Furuya-Kanamori, Lifeng Lin, Cuncun Lu, Omran A. H. Musa, Sunita Vohra

**Affiliations:** 1 Department of Population Medicine, College of Medicine, Qatar University, Al Jamiaa Street, P. O. Box, 2713 Doha, Qatar; 2 Department of Clinical Research Management, West China Hospital, Sichuan University, Chengdu, China; 3 Department of Pediatrics, Faculty of Medicine & Dentistry, University of Alberta, Edmonton, Alberta Canada; 4 West China School of Public Health and West China Fourth Hospital, Sichuan University, Chengdu, China; 5 UQ Centre for Clinical Research, Faculty of Medicine, University of Queensland, Brisbane, Australia; 6 Department of Statistics, Florida State University, Tallahassee, FL USA; 7 Evidence-Based Medicine Center, School of Basic Medical Sciences, Lanzhou University, Lanzhou, China; 8 Institute of Basic Research in Clinical Medicine, China Academy of Chinese Medical Sciences, Beijing, China; 9 Department of Psychiatry, Faculty of Medicine & Dentistry, University of Alberta, Edmonton, Alberta Canada

**Keywords:** Systematic review, Meta-analysis, Adverse events, Zero-events studies

## Abstract

**Backgrounds:**

Zero-events studies frequently occur in systematic reviews of adverse events, which consist of an important source of evidence. We aimed to examine how evidence of zero-events studies was utilized in the meta-analyses of systematic reviews of adverse events.

**Methods:**

We conducted a survey of systematic reviews published in two periods: January 1, 2015, to January 1, 2020, and January 1, 2008, to April 25, 2011. Databases were searched for systematic reviews that conducted at least one meta-analysis of any healthcare intervention and used adverse events as the exclusive outcome. An adverse event was defined as any untoward medical occurrence in a patient or subject in healthcare practice. We summarized the frequency of occurrence of zero-events studies in eligible systematic reviews and how these studies were dealt with in the meta-analyses of these systematic reviews.

**Results:**

We included 640 eligible systematic reviews. There were 406 (63.45%) systematic reviews involving zero-events studies in their meta-analyses, among which 389 (95.11%) involved single-arm-zero-events studies and 223 (54.93%) involved double-arm-zero-events studies. The majority (98.71%) of these systematic reviews incorporated single-arm-zero-events studies into the meta-analyses. On the other hand, the majority (76.23%) of them excluded double-arm-zero-events studies from the meta-analyses, of which the majority (87.06%) did not discuss the potential impact of excluding such studies. Systematic reviews published at present (2015-2020) tended to incorporate zero-events studies in meta-analyses than those published in the past (2008-2011), but the difference was not significant (proportion difference=−0.09, 95% CI −0.21 to 0.03, p = 0.12).

**Conclusion:**

Systematic review authors routinely treated studies with zero-events in both arms as “non-informative” carriers and excluded them from their reviews. Whether studies with no events are “informative” or not largely depends on the methods and assumptions applied, thus sensitivity analyses using different methods should be considered in future meta-analyses.

**Supplementary Information:**

The online version contains supplementary material available at 10.1186/s12916-021-02008-2.

## Background

In an era of evidence-based medicine, systematic reviews and meta-analyses represent the most important source of evidence and have been widely used to assess the effectiveness and safety of healthcare interventions [1]. In a meta-analysis, data from available studies on the same topic are quantitatively synthesized in an effort to reduce uncertainty and increase precision [2, 3]. Standard meta-analysis methods are based on large-sample approximation, and it can achieve reasonable statistical properties for assessment of efficacy. However, for assessment of safety, due to the potential low event rate and limited sample size, the observed events tend to be rare and often zero, and thus the assumption may no longer hold, further challenging data synthesis [4, 5].

Researchers generally divide studies with zero-events as single-arm-zero-events studies and double-arm-zero-events studies [6]. The former indicates that no events occur in one of the comparative arms; the latter indicates no events occur in both arms. For studies with zero-events in a single arm, there are several well-established methods (e.g., Peto odds ratio [7–9]) available to synthesize the information from such studies into a meta-analysis, and there is a unanimous agreement that such studies should not be excluded [5, 10]. However, for studies with zero-events in both arms, incorporating these studies is controversial and complex [11]. Standard meta-analysis methods focus on the intervention effects, but they seldom take care of the absolute risks of the arms; the relative measurements (i.e., odds ratio, risk ratio) and their variances are undefined for such studies and are therefore treated as non-informative in the meta-analysis. While an increasing body of evidence has shown that such studies are not necessarily non-informative from both clinical and statistical perspectives [6, 12, 13], depending on the methods or assumptions utilized for the synthesis (e.g., generalized linear mixed model, beta-binomial model [6, 14–19]).

The contradictory views regarding studies with zero events in both arms complicate decisions for systematic review authors and policymakers, who ultimately determine healthcare practice [20, 21]. The preference on dealing with such studies may influence future systematic review authors. It is, therefore, necessary and essential to keep abreast of how current meta-analyses deal with studies with zero events and make timely recommendations to promote the appropriate future practice. In this study, we conducted a survey of systematic reviews of adverse events to explore the frequency of occurrence of zero-events studies and how such studies were dealt with in the meta-analyses of these systematic reviews. We further discussed the issue of how to deal with the zero-events studies in meta-analysis.

## Methods

### Research protocol

An a priori protocol for this study was developed to formulate its design and conduct. Any protocol deviations were recorded (Additional file: Protocol). The current study is reported according to the PRIO-harms checklist [22].

### Data source

We searched PubMed for all relevant systematic reviews with adverse events as the exclusive outcome from January 1, 2015, to January 1, 2020. Although the limitation on the time range is somewhat arbitrary, we believe it would be representative of the current methods employed by meta-analysts to deal with zero-events studies. The primary search strategy was developed by a librarian and then discussed with the lead author (a methodologist) for further improvement. The complete search strategy is presented in the Additional file. In order to understand the potential changes in the preference of the methods for zero-events studies, we used a previous dataset that was collected in 2011, which included published systematic reviews of adverse events from January 1, 2008 to April 25, 2011. The details were documented in our published article [20].

### Inclusion criteria

We considered all systematic reviews that conducted at least one meta-analysis of any healthcare intervention and used safety as the exclusive outcome, regardless of whether the included studies were randomized controlled trials (RCT) or non-randomized studies of intervention (NRSI) or both. We defined adverse events as “any untoward medical occurrence in a patient or subject in clinical practice” [23], which could be an adverse effect, adverse reaction, harm, or complication associated with any healthcare intervention [24]. All studies of the meta-analyses were restricted to have binary outcomes. We did not consider meta-analyses assessing both effectiveness and safety; we also did not consider those synthesizing incidence or prevalence of adverse events that only had a single arm. Systematic reviews that were combined with publication of an original study and systematic reviews without meta-analysis were not considered [25]. In addition, the gray literature was not considered as we only focused on published meta-analyses. We did not consider the reference lists of included systematic reviews by hand search.

Two authors (XZ, CX) screened the literature searches separately through the online application Rayyan. We first screened the titles and abstracts to exclude those records obviously out of our scope (e.g., narrative review, meta-analysis of effectiveness). Full-text was obtained for included titles and abstracts or those that had disagreement to make a final decision. Any disagreements were discussed by the two authors until a final consensus was reached [26].

### Data collection

The current study focused on how zero-events studies were dealt with in meta-analyses of the included systematic reviews. The following baseline information was extracted (by Access 2016, Microsoft, USA): author name, year of publication, region of the corresponding author, type of meta-analysis employed, study type for meta-analysis, protocol registration, effect estimator (e.g., odds ratio, OR; risk ratio, RR; and risk difference, RD), and funding information. We were particularly interested in information about how zero-studies were handled. Specifically, we considered the following questions for each selected systematic review:

1. In the systematic review, did any meta-analysis contain zero-events studies?
2. If zero-events studies were involved, what were the types of zero-events studies (e.g., no event in a single arm, double arms, or both)?
3. If zero-events studies were involved, whether the authors specified methods to deal with zero-events studies?
4. If the authors specified methods, whether they clarified reasons for selecting the chosen method for handling zero-events studies?
5. If zero-events studies were involved, how were they dealt with by the authors?
6. Whether a sensitivity analysis was employed through at least one different synthesis method for dealing with zero-events studies?
7. For those that failed to synthesize studies with zero-events, did the authors discuss the potential impact of excluding such studies on the results?

The baseline information was extracted by one author (XZ) and then double-checked by another author (KJ). The information about how the meta-analyses dealt with zero-events studies was collected by one methodologist (CX) in this area and verified by two other statisticians (LFK, LL). It should be noted that for each included systematic review, two or more meta-analyses were likely to be involved. We only counted each systematic review once in terms of whether zero-events studies were included and how these studies were handled, because the pooling method was consistent across meta-analyses within the same systematic review. The judgment of whether a meta-analysis contained zero-events studies was based on the forest plot, baseline table, and/or text that indicated that studies with no events were excluded. For network meta-analyses, we only considered pairwise comparisons, if applicable.

### Data analysis

The baseline information was summarized in terms of frequencies and proportions. Our primary outcomes of interest were how meta-analyses with zero-events were dealt with; we classified them by zero-events types (i.e., single-arm-zero-events, double-arm-zero-events). Other relevant information was also summarized in terms of frequencies and proportions with an exact 95% confidence interval (CI). The exact method has been proven to be a valid solution for calculating proportions even when zero-events occur [27].

We further compared the proportions of studies excluding studies with zero-events in a single arm and in both arms for the two datasets (2015–2020 vs. 2008–2011) as planned in the protocol. The proportions were also compared in terms of whether an a priori protocol for handling zero events was developed or not. The proportion difference (PD) was used to measure the difference as a valid method for measuring the effect even in the presence of zero counts [26, 28]. Fisher’s exact test was used for sensitivity analysis when events were rare (i.e., less than 5). We did not use a continuity correction or Peto’s OR since the sample sizes in comparative groups were generally unbalanced, and these two methods could lead to large bias [6, 29]. Given the potentially high proportions of meta-analyses that excluded studies with zero events, we expected no PD involving double zero counts in this study.

All statistical analyses were performed by Stata (version 14.0/SE, Stata, College Station, TX) and MetaXL (version 5.3, EpiGear, Australia). The significance level was pre-specified as alpha = 0.05.

## Results

### Basic characteristics

**Fig. 1:**
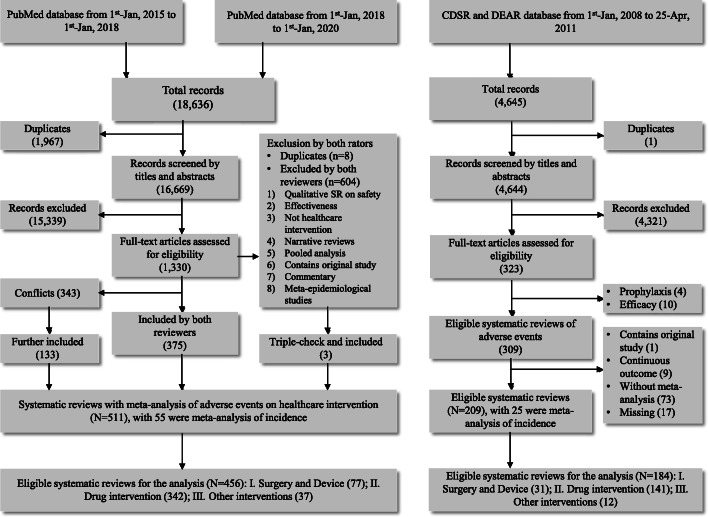
The literature screen process

In the dataset collected in 2011, we identified 309 potentially relevant systematic reviews, of which 17 were missing, leaving 292 with full texts for assessment. We further excluded those that did not conduct meta-analyses, had all outcomes as continuous variables, contained original research, or only investigated the incidence of adverse events. We included 184 systematic reviews for analysis (Fig. 1). In the dataset collected currently, we identified 511 eligible systematic reviews; all conducted meta-analyses. After excluding 55 reviews that only investigated the incidence of adverse events, we obtained 456 systematic reviews for analysis (Fig. 1).

Among the 640 systematic reviews in total, 483 (75.47%) focused on drug interventions, 108 (16.88%) on surgery or devices, and 49 (7.66%) on other interventions. These proportions were similar for the two datasets analyzed separately (Fig. 1). Most (n = 585, 91.41%) of these systematic reviews employed pairwise comparisons in the meta-analyses. Only four used individual participant data. Protocols were reported by 166 (25.94%) of the systematic reviews, and the majority (n = 474, 74.06%) failed to refer to any information on protocol development. Most systematic reviews used evidence from RCTs (n = 460, 71.88%) for their meta-analyses; 89 (13.91%) used evidence from NRSIs. The most widely used effect estimators were the OR and RR; the proportions were 41.56% and 49.38%, respectively. The RD was seldom used to measure the effect (n = 15, 2.34%) (Table 1).

**Table 1:** Basic characteristics of included systematic reviews on adverse events

Basic characteristics	No. of systematic reviews (N = 640)
**Year**	
2008–2011	184 (28.75%)
2015–2020	456 (71.25%)
**Region of corresponding author**	
Africa	15 (2.34%)
America (North and South)	193 (30.16%)
Asia	193 (30.16%)
European	219 (34.22%)
Oceania	20 (3.13%)
**Intervention**	
Drug	483 (75.47%)
Surgery or device	108 (16.88%)
Others (e.g., complementary medicine)	49 (7.66%)
**Type of meta-analysis**	
Pairwise meta-analysis	585 (91.41%)
Network meta-analysis	17 (2.66%)
Both pairwise and network meta-analyses	32 (5.0%)
Association	2 (0.31%)
Individual participant data	4 (0.63%)
**Protocol**	
Yes	166 (25.94%)
No	474 (74.06%)
**Type of study for meta-analysis**	
Randomized controlled trial (RCT)	460 (71.88%)
Non-randomized study of intervention (NRSI)	89 (13.91%)
Both RCT and NRSI	87 (13.59%)
Not reported	4 (0.63%)
**Effect estimator**	
Odds ratio, including Peto’s OR	266 (41.56%)
Risk ratio or relative risk	316 (49.38%)
Hazard ratio	9 (1.41%)
Risk difference	15 (2.34%)
Others (e.g., mixed use, Chi2, coef.)	34 (5.31%)
**Systematic review with zero-events studies in meta-analysis**	
Yes	406 (63.45%)
No	88 (13.75%)
No data available for judgment	146 (22.80%)
**Power analysis**	
Yes	16 (2.50%)
No	624 (97.5%)

For the 640 systematic reviews, 146 (n = 146, 22.80%) did not provide sufficient information on whether zero-events studies were involved, and 406 (63.45%) systematic reviews encountered zero-events studies in their meta-analyses. Among these 406 reviews, 90 were identified from the first dataset, and 316 were from the second dataset. There was a significantly higher proportion of zero-events studies in systematic reviews published from 2015 to 2020 than those published from 2008 to 2011 (67.11% vs. 48.91%, p < 0.001). For the 406 systematic reviews with zero-events studies in their meta-analyses, 174 (42.86%) involved single-arm-zero-events studies only, 7 (1.72%) involved double-arm-zero-events studies only, and 208 (51.23%) involved both single- and double-zero-events studies. Only 131 (32.27%) systematic reviews reported methods to deal with zero-events studies in meta-analyses. Among these 131 systematic reviews, only 44 (33.59%) provided reason(s) for selecting relevant methods to deal with zero-events studies (Table 2).

**Table 2:** Methodological information for dealing with zero-events studies

Methodological information for dealing with zero-events studies	Yes (%, exact CI)	No (%, exact CI)	NC or NA (%, exact CI)	Total
1) In the systematic review, did any meta-analysis contain zero-events studies?	406 (63.44, 59.57 to 67.18)	88 (13.75, 11.18 to 16.66)	146 (22.81, 19.62 to 26.26)	640
2) If zero-events studies were involved, what were the types of zero-events studies?				406
• Single-arm-zero-events only	174 (42.86, 37.99 to 47.83)	‐	‐	
• Double-arm-zero-events only	7 (1.72, 0.70 to 3.52)	‐	‐	
• Both single and double	208 (51.23, 46.25 to 56.19)	‐	‐	
• At least containing single-arm-zero-events study	7 (1.72, 0.70 to 3.52)	‐	‐	
• At least containing double-arm-zero-events study	8 (1.97, 0.85 to 3.85)	‐	‐	
• Cannot be judged	2 (0.49, 0.06 to 1.77)	‐	‐	
3) If zero-events studies were involved, whether the authors specified methods to deal with zero-events studies?	131 (32.27, 27.74 to 37.05)	275 (67.73, 62.95 to 72.26)	0 (0.00, 0.00 to 0.90)	406
4) If the authors specified methods, whether they clarified reasons for selecting the method for zero-events studies?	44 (33.59, 25.58 to 42.36)	87 (66.41, 57.64 to 74.42)	0 (0.00, 0.00 to 2.78)	131
5) If zero-events studies were involved, how zero-events studies were dealt with by the authors?	See detailed methods in Fig. 2			
• Discarding single-arm-zero-events studies (174 + 208 + 7 = 389)	3 (0.77, 0.16 to 2.24)	384 (98.71, 97.03 to 99.58)	2 (0.51, 0.06 to 1.85)	389
• Discarding double-arm-zero-events studies (7 + 208 + 8 = 223)	170 (76.23, 70.09 to 81.66)	50 (22.42, 17.12 to 28.47)	3 (1.35, 0.28 to 3.85)	223
6) Whether a sensitivity analysis was employed through at least one different synthesis method for dealing with zero-events studies?	53 (13.05, 9.93 to 16.73)	353 (86.95, 83.27 to 90.07)	0 (0.00, 0.00 to 0.90)	406
7) For those failed to synthesize studies with zero-events, did the authors discuss about the potential impact of such studies (e.g., discarding them) on the results?	22 (12.94, 8.29 to 18.94)	148 (87.06, 81.06 to 91.71)	0 (0.00, 0.00 to 2.15)	170

*NC* not clear; *NA* not applicable

### Dealing with zero-events studies in meta-analysis: in the past

**Fig. 2:**
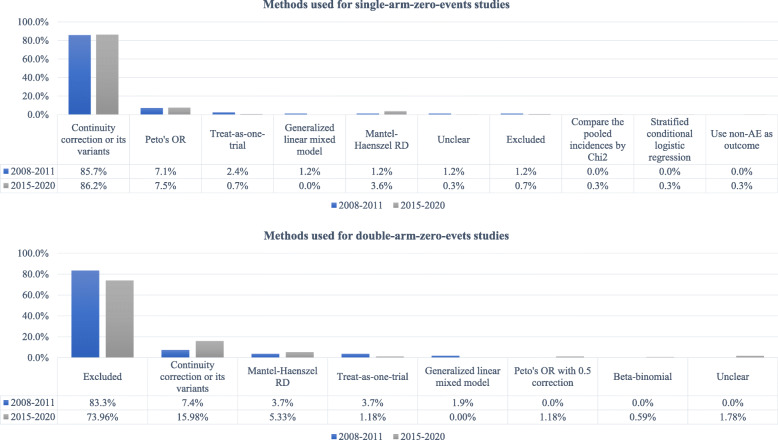
Methods for dealing with zero-events studies in eligible systematic reviews

In the 90 systematic reviews involving zero-events studies in meta-analyses in 2008–2011, 84 involved single-arm-zero-events studies, and 54 involved double-arm-zero-events studies. Figure 2 presents the methods used to deal with zero-events studies.

Among the 84 systematic reviews with single-arm-zero-events studies, 83 incorporated evidence of these studies in meta-analyses, and 1 (1.19%) excluded these studies from meta-analyses. The most commonly used method for dealing with single-arm-zero-events studies was the continuity correction or its variants (e.g., empirical correction, opposite-arm correction [29]) (n = 72), followed by Peto’s OR (n = 6, 7.14%). Other methods, such as the generalized linear mixed model (n = 1, 1.19%) and Mantel-Haenszel RD (n = 2, 2.38%), were seldom used. For 1 review, we could not judge which method they employed. We also identified two (2.38%) systematic reviews that used the “collapsed table” (also refers to “treat-as-one-trial”) method for the pooling; however, without weighting, this method could be misleading due to Simpson’s paradox (i.e., a trend that appears in individual studies but disappears or reverses after unweighted incorporation) [30].

For 54 systematic reviews with double-arm-zero-events studies, the most commonly used procedure was excluding these studies (n = 45, 83.33%) from the meta-analyses. Importantly, 40 (88.89%) of these 45 systematic reviews did not discuss any potential impact of excluding such studies on the results. The remaining 9 systematic reviews used the continuity correction or its variants (n = 4, 7.4%), Mantel-Haenszel RD (n = 2, 3.70%), “collapsed table” (n = 2, 3.70%), or generalized linear mixed model (n = 1, 1.85%) to synthesize evidence from these studies in meta-analyses (Fig. 2).

### Dealing with zero-events studies in meta-analysis: at present

In the 316 systematic reviews involving zero-events studies in meta-analyses in 2015–2020, 305 involved single-arm-zero-events studies, and 169 involved double-arm-zero-events studies.

Among the 305 systematic reviews with single-arm-zero-events studies in meta-analyses, the most commonly used method for dealing with single-arm-zero-events studies remained continuity correction or its variants (n = 263, 86.23%), followed by Peto’s OR (n = 23, 7.54%), and then the Mantel-Haenszel RD (n = 11, 3.61%). Other methods, such as the “collapsed table” (n = 2, 0.66%), stratified conditional logistic regression (n = 1, 0.33%), using non-adverse events as outcomes (n = 1, 0.33%), were seldom used. Again, for 1 (0.33%) systematic review, we could not judge which method they employed. It should be highlighted that two (0.66%) systematic reviews excluded such studies from their meta-analyses.

For 169 systematic reviews with double-arm-zero-events studies, the most commonly used procedure was excluding these studies (n = 125, 73.96%) from the meta-analyses. Again, in these 125 systematic reviews, 108 (86.40%) failed to discuss the potential impact of excluding zero-events studies on the results. There were 41 systematic reviews that used the continuity correction or its variants (n = 27, 15.98%), Mantel-Haenszel RD (n = 9, 5.33%), “collapsed table” (n = 2, 1.18%), Peto’s OR with 0.5 correction (n = 2, 1.18%), and beta-binomial model (n = 1, 0.59%) to synthesize evidence of such studies in meta-analyses (Fig. 2). However, for 3 (1.78%) reviews, we could not judge which method they employed.

### Comparing the present to the past

Compared to the past, there were more systematic reviews incorporating evidence from double-arm-zero-events studies into meta-analyses rather than excluding them. This change was primarily attributable to the increased use of the continuity correction (or its variants) method and Mantel-Haenszel RD (Fig. 2). We further quantitatively compared the proportions of excluding zero-events studies for these two time periods (2015–2020 vs. 2008–2011); see Additional file: Fig.S1. Our results suggested that although recent systematic reviews tended to incorporate zero-events studies in meta-analyses, the difference was not significant from either the practical or statistical perspective (for single-arm-zero-events: PD=−0.01, 95% CI −0.03, 0.02, p = 0.67; for double-arm-zero-events: PD=−0.09, 95% CI −0.21, 0.03, p = 0.12). Sensitivity analysis was employed by Fisher’s exact test, which showed consistent results (p = 0.52).

### Additional analysis

The proportions of excluding zero-events studies were also compared in terms of whether a protocol was reported in those systematic reviews (Additional file: Fig.S2). Our results suggested no substantial difference (for single-arm-zero-events: PD=−0.00, 95% CI −0.02, 0.01, p = 0.64; for double-arm-zero-events: PD=−0.07, 95% CI −0.19, 0.06, p = 0.29) in the proportions of excluding for those with and without a protocol. Sensitivity analysis was employed by Fisher’s exact test, which showed consistent results (p = 1.00).

## Discussion

### Main findings

In this study, we conducted a large-scale survey on systematic reviews with meta-analyses of adverse events associated with various healthcare interventions to examine how they dealt with zero-events studies. Our results suggested that in the majority (76.23%) of the systematic reviews, studies with zero events in both arms were excluded from meta-analyses. This was more common in systematic reviews published earlier (83.33%). Although an increasing proportion of systematic reviews attempted to synthesize evidence from studies with zero events in both arms in recent years, the majority still excluded such studies (73.95%).

### Implications

In our study, we noticed that among the majority of systematic reviews that excluded zero-events studies, only a small proportion (12.94%, Table 2) discussed the potential impact of excluding zero-events studies in both arms on their results. Although there is still controversy among statisticians for whether studies with zero-events in both arms should be synthesized in a meta-analysis, it is not doubtful that systematic review authors should inform and discuss the results of such studies [20]. Clinical trials might tend to under-report adverse events in the publications [31, 32], so that the zero-events may be caused by the selective reporting. This practice is dangerous because such selective reporting may lead to a large amount of bias for the meta-results. From this point, simply treating studies with zero-events studies as “non-informative” may be unreasonable because this leads to neglecting such studies by systematic review authors.

The controversy of dealing with studies with zero events in both arms is mainly due to the methods used and assumptions of the absolute risks. As we mentioned earlier, the standard methods make no assumptions on the absolute risks of the arms; when there are no events in both arms, the relative measures cannot be defined. Böhning and Sangnawakij recently compared the likelihood of excluding (based on a conditional binomial model) and including (based on a Poisson regression model) studies with zero-events in both arms by mathematical derivations; they found that such studies do not contribute to the likelihood [19], supporting the exclusion of studies with no events. However, this may not be the case under some other models (e.g., multilevel logistic regression model [14], beta-binomial [18]), where the relative measure could be defined based on certain assumptions. Many approaches have been proposed to include studies with no events and compare the corresponding results with those excluding such studies [6, 11–13, 18, 33–35]. Considering the opinions of researchers on both sides, perhaps sensitivity analysis using different methods for including and excluding studies with no events should be considered in future meta-analyses [29, 36].

We noticed that for those meta-analyses that synthesize the information from studies with no events, the majority used the continuity correction or its variants. It has been criticized that the continuity correction and its variants add pseudo-observations to the data set that potentially lead to large bias in the estimates [6, 11]. Statisticians have advocated avoiding the use of continuity correction in this situation and provided several better solutions (e.g., beta-binomial [18]) that do not need additional information [6]. Unfortunately, it seems that more systematic review authors continued to use continuity correction for studies with no events over time (16% at present vs. 7.4% in the past). This implies that most of the systematic review authors may not be aware of the problems of continuity correction and more efforts are needed to improve the common practice.

In our study, we did not detect a significant effect of reporting a protocol on reducing the proportion of excluding zero-events studies. This is not surprising as our recent research has shown that most systematic review protocols on intervention safety did not develop a synthesis plan for dealing with zero-events studies [27]. This again indicates that systematic review authors routinely neglect zero-events studies or may be inexperienced in handling such analyses. We believe a well-established protocol with a clear synthesis plan for zero-event studies would benefit the data synthesis process in systematic reviews of adverse events.

### How to make full use of zero-events studies in meta-analysis?

There are currently several well-established methods that are considered appropriate to synthesize studies with no events, including the Mantel-Haenszel RD [37], the exact *p* value function [38], and several one-stage methods [6, 11, 14–19, 39]. The Mantel-Haenszel RD has been justified for its advantage for dealing with studies with no events as RD is well-defined in such cases and does not need any post hoc correction or prior information. Bradburn et al. found that Mantel-Haenszel RD could achieve almost unbiased estimation, while the limitation is that RD showed low statistical power that makes it not the optimal choice for meta-analysis of rare events [40]. Another problem of RD could be that, for studies with the same duration of intervention and control arms, the duration could be canceled out when using RR but not for RD, so RD is not invariant under study duration variation, and one should consider the potential impact of duration on RD [19]. However, among the two-stage methods, perhaps RD is one of the good choices, and in some situations (e.g., all studies are zero-events studies) it is the only choice, considering that OR and RR could not be defined in the majority of the two-stage methods [41, 42].

Kuss has summarized existing methods that do not require a post hoc correction that could be considered for the synthesis of studies with no events in both arms [6]. In the meanwhile, Böhning et al. proposed the zero-inflated Poisson models to deal with excess zeros for meta-analysis with zero-events [11, 19]. Systematic review authors are strongly recommended to refer to these methods for their meta-analysis when zero-events studies are involved. One problem could be that the applicability of these methods differs a lot, and most of the systematic review authors may not know which of them are feasible for their meta-analysis. For example, when the total event count in one of the arms or both arms is zero in the meta-analysis, one-stage methods are no longer feasible. A recent framework for meta-analysis with zero-events studies proposed by our group could be considered to help systematic review authors appropriately make use of zero-events in meta-analysis [21]. Based on the total event count within the meta-analysis and zero-events type of individual studies, we can classify meta-analyses with zero-events studies into six subtypes (see Additional file: Fig.S3 for details). For each subtype of meta-analysis, the applicability of available methods could be well distinguished. They are listed under the diagram of the framework (Additional file: Fig.S4), and systematic review authors can easily identify one or more appropriate methods for their meta-analysis [21].

Some further recommendations can be considered in practice as an effort to make full use of zero-events studies in meta-analysis.

- First, standard meta-analysis methods, say, the inverse-variance method with continuity correction, are not recommended as the primary choice for meta-analysis with zero-events studies [5, 6, 29].
- Second, in case that the total event count of *each arm* of a meta-analysis is at least 10, the one-stage methods, e.g., the mixed Poisson regression models, the beta-binomial model, the generalized linear mixed models, and the generalized estimating equations, could be considered as the primary option [5, 6, 11, 16, 18, 19, 21, 43].
- Third, when the total event count of a meta-analysis is < 10 or one-stage methods cannot achieve convergence, two-stage methods such as Peto’s OR, Mantel-Haenszel OR, and Mantel-Haenszel RD could be considered, while Peto’s OR and Mantel-Haenszel OR can only be considered when there are no studies with no events in both arms [6, 26, 38, 44].

### Strengths and limitations

To the best of our knowledge, this is the first study that investigated the methods used in published systematic reviews for dealing with zero-events studies. Our findings provided a clear picture of the preference of systematic review authors to exclude data when dealing with zero-events studies in the past and at present. We provided practical recommendations to help systematic review authors appropriately make use of zero-events studies in meta-analysis. These findings will have implications for methodology guidelines, evidence-based practice, and healthcare policy.

Several important limitations need to be highlighted. First, the information of whether a systematic review involved zero-events studies in its meta-analysis relied on the results reported by the systematic review. We identified that 23% of systematic reviews failed to report the case counts and sample sizes of the individual studies in meta-analyses, making it difficult to determine if they contained zero-events studies. One could consider this a type of missing data (missing not at random [44, 45]) and therefore advocate caution about the potential bias (on the estimation of the proportions of excluding zero-events studies) caused by these missing data. Second, the information of how zero-events studies were dealt with was collected by a methodologist and verified by two statisticians; however, we were unable to judge how zero-events studies were dealt with in 5 systematic reviews. In addition, although we repeated the analyses for zero-events studies for each systematic review, there were 12 systematic reviews in which we could not repeat the results according to the methods claimed in the reviews. They may also introduce some bias in the estimated proportions. Despite these potential issues, we believe they did not change the fact that zero-events studies were excluded by most systematic review authors.

## Conclusions

Based on our findings, the majority of systematic review authors excluded studies with zero events in both arms from meta-analyses and treated them as “non-informative.” This preference was popular in systematic reviews published 10 years ago and remains popular at present. Whether studies with no events are “informative” or not largely depends on the methods and assumptions applied; thus, sensitivity analyses using different methods should be considered in future meta-analyses.

## Supplementary Information

**Additional file 1.** Additional materials including the research protocol, search strategy, list of included studies, and additional figures for the main context.

## Data Availability

Data could be obtained from the corresponding author upon request.
