# Supplementary material for: Utilization of the evidence from studies with no events in meta-analyses of adverse events: an empirical investigation
Source: BMC Med. 2021 Jun 15;19:141. doi: 10.1186/s12916-021-02008-2 (PMC8204528; doi:10.1186/s12916-021-02008-2)
Supplement: Supplementary file 1 — Additional file 1. Additional materials including the research protocol, search strategy, list of included studies, and additional figures for the main context. [file 12916_2021_2008_MOESM1_ESM.docx]

**PROTOCOL**

**Handling methods for zero-events studies in meta-analyses of adverse events: meta-epidemiological study**

**Drafted by:** Chang Xu^1,2^

1. Department of Population Medicine, College of Medicine, Qatar University, Al Jamiaa Street, P. O. Box 2713, Doha, Qatar;
2. Chinese Evidence-based Medicine Center, West China Hospital, Sichuan University, Chengdu, China;

## ^🖂^Correspondence to:

Dr. Chang Xu - [xuchang2016@runbox.com](mailto:xuchang2016@runbox.com)

Department of Population Medicine, College of Medicine, Qatar University, Al Jamiaa Street, P. O. Box 2713, Doha, Qatar;

## Research question

In the era of evidence-based medicine, systematic review and meta-analysis contributes the most important source of evidence and has been widely used to assess the effectiveness and safety of healthcare intervention. In a meta-analysis, data from available studies on the same topic are quantitively synthesized as an effort to reduce the uncertainty and increase the credibility. It has been well-recognized that meta-analysis could achieve reasonable statistical properties for efficacy assessment; however, for safety assessment, due to the potential low events rate and limited sample size, the observed events tend to be rare and often zero, make the data synthesis challengeable.

For studies with zero-events in a single arm, several well-established methods (e.g. Peto Odds ratio, continuity correction, Mantel–Haenszel) were available to synthesis the information of such studies into meta-analysis, and there is a unanimous agreement that such studies should be incorporated into meta-analysis. For studies with zero-events in both arms, researchers either discard them from the meta-analysis or incorporate them through some sophisticated methods (e.g. Bayesian, generalized linear mixed model (GLMM)). Due to the nature of such data, the results were most likely unstable and susceptive to the methods employed, which further largely impacted the credibility of the conclusions of a meta-analysis. Understanding how such studies were handled is necessarily important, which will have implications for further guidelines, practice and policy.

## Searches

We will search PubMed for all related meta-analyses of randomized controlled trials for adverse events. The primary search strategy was developed by a librarian, and then discussed with the lead author for further adjustment (see Supplements). Considering the huge number of meta-analysis published in recent decades, we will limit our search to recent 5 years (2015/01/01 to 2020/01/01). To better understand how studies of zero-events were handled in meta-analyses of adverse events in the past, we will also include our previous dataset for meta-analyses of rare events (January 2008 to April 2011).

Grey literature will not be considered as we only aimed at published meta-analyses. We hypothesized that these published meta-analyses would be of more deliberate in dealing with zero-events studies after strict peer review process. Considering the sample would be representative, we will not use hand search for the reference lists of each systematic reviews and meta-analyses.

## Types of study to be included

Meta-analyses of randomized controlled trials with adverse events as the unique outcome(s). We defined adverse events as “any unintended effect or effects of an intervention”, which could be an adverse event, adverse effect, adverse reaction, harms, or complications associated with any healthcare intervention. And all “adverse events” should be binary outcomes. We will not consider those meta-analyses assessing both effectiveness and safety; we will also not consider meta-analyses of incidence or prevalence of adverse events that with only a single arm. Systematic reviews without a meta-analysis will be excluded.

## Context

This study will focus on how zero-events studies were dealt with in meta-analyses of adverse events. We will summarize this information for zero-events in a single arm and zero-events in both arms separately, with a special focus on the later. These include:

***Main information: Meta-analysis with zero-events in both arms***

1. Whether studies with zero-events in both arms were synthesized;
2. Which effect estimator (e.g. OR, RR, RD) was used;
3. Which synthesis method was used;
4. Whether a sensitivity analysis was employed through at least one different synthesis methods;
5. For those failed to synthesize studies with zero-events in both arms, whether any further analysis was conducted to evaluate the potential impact (of excluding such studies) on the results;

***Main information: Meta-analysis with zero-events in*** ***single arm***

1. which effect estimator (e.g. OR, RR, RD) was used;
2. which synthesis method was used;
3. whether a sensitivity analysis was employed through at least one different synthesis methods;

***Other information***

1. How between-study heterogeneity was measured, and if conducted, whether a substantial heterogeneity was detected (According to Cochrane’s standard, for example, I^2^ > 50%);
2. How publication bias was measured, and if conducted, whether a publication bias was detected (According to the measuring method itself, for p-value driven method, we set the cut-off point as p = 0.10);

We will further categorize the methods used in meta-analyses as frequentist and Bayesian framework, where for each framework, the detailed methods will be summarized. Such information will be compared for meta-analyses published in recent 5 years and those published in 2008 to 2011.

## Main outcome(s)

The primary outcome is the percentage (and ranks) of the methods used for dealing with zero-events studies. The co-primary outcome is the proportion of meta-analyses that zero-events studies were correctly dealt with over years. We defined those meta-analyses discard zero-events studies as incorrectly dealt with.

## Assessment of risk of bias

The PRISMA-harms and AMSTAR 2.0 checklists will be used to assess the potential bias (quality) on reporting and methodology. This will be treated as separate paper(s).

## Data extraction (selection and coding)

Two authors will screen the literature for eligibility independently through the Rayyan online app, since this app allows a restrict blinding for the two rators to ensure the process was independent. The titles and abstracts will be first screened and those obviously not meet the criteria will be excluded; then the full-text will be checked for final decision.

Data exaction (above mentioned information in “Context” section) will be employed by an experienced statistician (master degree), and will be double checked by the lead author. Baseline characteristics such as author name, number of authors, publication year, region of first author, use of reporting guidance, use of GRADE, funding information, type of meta-analyses (e.g. generic, network) will also be extracted.

## Analysis of subgroups or subsets

- Eligible meta-analyses published in recent 5 years (2015/01/01 to 2020/01/01)
- Eligible meta-analyses published from Jan-2008 to April-2011.

## Contact details for further information

[xuchang2016@runbox.com](mailto:xuchang2016@runbox.com)

## Conflicts of interest

We declare no conflict of interest.

## Funding

This study did not receive any financial supporting.

Stage of review
Review Ongoing: Literature search has been conducted and is currently on literature screen (By 28^th^-July, 2020).

**Deviations of protocol and the article**

Some minor changes were made as follows: First, in the protocol, we planned to collect the information of how between-study heterogeneity was measured and how publication bias was detected. This was not done as such information was already represented in our other recent study. Second, one of our aims was to categorize the methods for dealing with zero-events studies in terms of the frequentist and Bayesian frameworks. However, we only identified one study using Bayesian methods, so we removed this aim. Third, our primary “population” was meta-analyses of randomized controlled trials (RCTs) on intervention safety. However, during the search procedure, we realized that many meta-analyses with non-randomized studies of intervention (NRSI) also faced the zero-events problem. We therefore decided to include meta-analyses with non-randomized studies as well.

## Supplements-Search strategy (PubMed, conducted at 28^th^-July, 2020)

1. "Systematic Reviews as Topic"[Mesh] OR "Systematic Review" [Publication Type] OR "Meta-Analysis as Topic"[Mesh] OR "Meta-Analysis" [Publication Type] OR "meta-analysis"[Title/Abstract] OR "meta analysis"[Title/Abstract] OR "systematic review"[Title/Abstract]
2. "randomized controlled trials as topic"[MeSH Major Topic] OR "clinical trials as topic"[MeSH Major Topic] OR "controlled clinical trials as topic"[MeSH Major Topic]
3. "randomized controlled trial*"[Title/Abstract] OR "controlled clinical trial*"[Title/Abstract] OR "clinical trial*"[Title/Abstract] OR "controlled trial*"[Title/Abstract] OR "trial*"[Title/Abstract]
4. "safety"[Title/Abstract] OR "harm*"[Title/Abstract] OR safe[Title/Abstract] OR poisoning[Title/Abstract] OR toxicity[Title/Abstract] OR tolerability[Title/Abstract] OR "complication*"[Title/Abstract] OR "adverse event*"[Title/Abstract] OR "adverse outcome*"[Title/Abstract] OR "untoward effect*"[Title/Abstract] OR "side effect*"[Title/Abstract] OR adverse n2 reaction[Title/Abstract]
5. #2 or #3
6. #1 AND #4 AND #5
7. Protocol[Title] OR overview [Title] OR "narrative review" [Title]
8. #6 NOT #7
9. (#8) AND (("2018/01/02"[Date - Publication]: "2020/01/01"[Date - Publication])) AND (humans[Filter]) Filters: Humans
10. (#8) AND (("2015/01/01"[Date - Publication]: "2018/01/01"[Date - Publication])) AND (humans[Filter]) Filters: Humans
11. #9 or # 10

**List of included studies (2015-2020)**

1. Schiphorst AH, Verweij NM, Pronk A, Borel Rinkes IH, Hamaker ME. Non-surgical complications after laparoscopic and open surgery for colorectal cancer - A systematic review of randomised controlled trials. Eur J Surg Oncol. 2015;41(9):1118-1127. doi:10.1016/j.ejso.2015.04.007
2. Fabisiak A, Włodarczyk M, Fabisiak N, Storr M, Fichna J. Gastrointestinal Adverse Events of Cannabinoid 1 Receptor Inverse Agonists suggest their Potential Use in Irritable Bowel Syndrome with Constipation: A Systematic Review and Meta-Analysis. J Gastrointestin Liver Dis. 2019;28(4):473-481. Published 2019 Dec 9. doi:10.15403/jgld-265
3. Varma A, Zis P. Nocebo effect in myasthenia gravis: systematic review and meta-analysis of placebo-controlled clinical trials. Acta Neurol Belg. 2019;119(2):257-264. doi:10.1007/s13760-019-01143-1
4. Eke AC, Shukr GH, Chaalan TT, Nashif SK, Eleje GU. Intra-abdominal saline irrigation at cesarean section: a systematic review and meta-analysis. J Matern Fetal Neonatal Med. 2016;29(10):1588-1594. doi:10.3109/14767058.2015.1055723
5. Ouyang AJ, Lv YN, Zhong HL, et al. Meta-analysis of digoxin use and risk of mortality in patients with atrial fibrillation. Am J Cardiol. 2015;115(7):901-906. doi:10.1016/j.amjcard.2015.01.013
6. Yamada A, Wang J, Komaki Y, Komaki F, Micic D, Sakuraba A. Systematic review with meta-analysis: risk of new onset IBD with the use of anti-interleukin-17 agents. Aliment Pharmacol Ther. 2019;50(4):373-385. doi:10.1111/apt.15397
7. Verrotti A, Prezioso G, Di Sabatino F, Franco V, Chiarelli F, Zaccara G. The adverse event profile of levetiracetam: A meta-analysis on children and adults. Seizure. 2015;31:49-55. doi:10.1016/j.seizure.2015.07.004
8. Sirker A, Kwok CS, Kotronias R, et al. Influence of access site choice for cardiac catheterization on risk of adverse neurological events: A systematic review and meta-analysis. Am Heart J. 2016;181:107-119. doi:10.1016/j.ahj.2016.06.027
9. Alexander PE, Barty R, Fei Y, et al. Transfusion of fresher vs older red blood cells in hospitalized patients: a systematic review and meta-analysis. Blood. 2016;127(4):400-410. doi:10.1182/blood-2015-09-670950
10. Brotis AG, Tasiou A, Paterakis K, Tzerefos C, Fountas KN. Complications Associated with Surgery for Thoracic Disc Herniation: A Systematic Review and Network Meta-Analysis. World Neurosurg. 2019;132:334-342. doi:10.1016/j.wneu.2019.08.202
11. Ahmed AM, Moahammed AT, Mattar OM, et al. Surgical treatment of diverticulitis and its complications: A systematic review and meta-analysis of randomized control trials. Surgeon. 2018;16(6):372-383. doi:10.1016/j.surge.2018.03.011
12. Capogrosso Sansone A, Mantarro S, Tuccori M, et al. Safety Profile of Certolizumab Pegol in Patients with Immune-Mediated Inflammatory Diseases: A Systematic Review and Meta-Analysis. Drug Saf. 2015;38(10):869-888. doi:10.1007/s40264-015-0336-2
13. Schulman AR, Popov V, Thompson CC. Randomized sham-controlled trials in endoscopy: a systematic review and meta-analysis of adverse events. Gastrointest Endosc. 2017;86(6):972-985.e3. doi:10.1016/j.gie.2017.07.046
14. Keir A, Pal S, Trivella M, et al. Adverse effects of red blood cell transfusions in neonates: a systematic review and meta-analysis. Transfusion. 2016;56(11):2773-2780. doi:10.1111/trf.13785
15. de Carvalho ALR, Vital RB, de Lira CCS, et al. Laryngeal Mask Airway Versus Other Airway Devices for Anesthesia in Children With an Upper Respiratory Tract Infection: A Systematic Review and Meta-analysis of Respiratory Complications [published correction appears in Anesth Analg. 2019 Feb;128(2):e37]. Anesth Analg. 2018;127(4):941-950. doi:10.1213/ANE.0000000000003674
16. Costa APF, Cobucci RNO, da Silva JM, da Costa Lima PH, Giraldo PC, Gonçalves AK. Safety of Human Papillomavirus 9-Valent Vaccine: A Meta-Analysis of Randomized Trials. J Immunol Res. 2017;2017:3736201. doi:10.1155/2017/3736201
17. Gafter-Gvili A, Gurion R, Raanani P, Shpilberg O, Vidal L. Bendamustine-associated infections-systematic review and meta-analysis of randomized controlled trials. Hematol Oncol. 2017;35(4):424-431. doi:10.1002/hon.2350
18. Tricco AC, Soobiah C, Blondal E, et al. Comparative safety of serotonin (5-HT3) receptor antagonists in patients undergoing surgery: a systematic review and network meta-analysis. BMC Med. 2015;13:142. Published 2015 Jun 18. doi:10.1186/s12916-015-0379-3
19. Toner AJ, Ganeshanathan V, Chan MT, Ho KM, Corcoran TB. Safety of Perioperative Glucocorticoids in Elective Noncardiac Surgery: A Systematic Review and Meta-analysis. Anesthesiology. 2017;126(2):234-248. doi:10.1097/ALN.0000000000001466
20. Kirby A, Hobson RP, Burke D, Cleveland V, Ford G, West RM. Appendicectomy for suspected uncomplicated appendicitis is associated with fewer complications than conservative antibiotic management: a meta-analysis of post-intervention complications. J Infect. 2015;70(2):105-110. doi:10.1016/j.jinf.2014.08.009
21. Hill AM, Mitchell N, Hughes S, Pozniak AL. Risks of cardiovascular or central nervous system adverse events and immune reconstitution inflammatory syndrome, for dolutegravir versus other antiretrovirals: meta-analysis of randomized trials. Curr Opin HIV AIDS. 2018;13(2):102-111. doi:10.1097/COH.0000000000000445
22. Wei A, Gu Z, Li J, et al. Clinical Adverse Effects of Endothelin Receptor Antagonists: Insights From the Meta-Analysis of 4894 Patients From 24 Randomized Double-Blind Placebo-Controlled Clinical Trials. J Am Heart Assoc. 2016;5(11):e003896. Published 2016 Oct 26. doi:10.1161/JAHA.116.003896
23. Bertrand A, Kostine M, Barnetche T, Truchetet ME, Schaeverbeke T. Immune related adverse events associated with anti-CTLA-4 antibodies: systematic review and meta-analysis. BMC Med. 2015;13:211. Published 2015 Sep 4. doi:10.1186/s12916-015-0455-8
24. Dahal A, Bellows BK, Sonpavde G, et al. Incidence of Severe Nephrotoxicity With Cisplatin Based on Renal Function Eligibility Criteria: Indirect Comparison Meta-analysis. Am J Clin Oncol. 2016;39(5):497-506. doi:10.1097/COC.0000000000000081
25. Veroniki AA, Rios P, Cogo E, et al. Comparative safety of antiepileptic drugs for neurological development in children exposed during pregnancy and breast feeding: a systematic review and network meta-analysis. BMJ Open. 2017;7(7):e017248. Published 2017 Jul 20. doi:10.1136/bmjopen-2017-017248
26. Katsanos AH, Schellinger PD, Köhrmann M, et al. Fatal oral anticoagulant-related intracranial hemorrhage: a systematic review and meta-analysis. Eur J Neurol. 2018;25(10):1299-1302. doi:10.1111/ene.13742
27. Nagy A, Mátrai P, Hegyi P, et al. The effects of TNF-alpha inhibitor therapy on the incidence of infection in JIA children: a meta-analysis. Pediatr Rheumatol Online J. 2019;17(1):4. Published 2019 Jan 18. doi:10.1186/s12969-019-0305-x
28. Neto AS, Hemmes SN, Barbas CS, et al. Association between driving pressure and development of postoperative pulmonary complications in patients undergoing mechanical ventilation for general anaesthesia: a meta-analysis of individual patient data [published correction appears in Lancet Respir Med. 2016 Jun;4(6):e34]. Lancet Respir Med. 2016;4(4):272-280. doi:10.1016/S2213-2600(16)00057-6
29. Kaneoka A, Pisegna JM, Saito H, et al. A systematic review and meta-analysis of pneumonia associated with thin liquid vs. thickened liquid intake in patients who aspirate. Clin Rehabil. 2017;31(8):1116-1125. doi:10.1177/0269215516677739
30. Almufleh A, Ramirez FD, So D, et al. H2 Receptor Antagonists versus Proton Pump Inhibitors in Patients on Dual Antiplatelet Therapy for Coronary Artery Disease: A Systematic Review. Cardiology. 2018;140(2):115-123. doi:10.1159/000489165
31. Doleman B, Moppett IK. Is early hip fracture surgery safe for patients on clopidogrel? Systematic review, meta-analysis and meta-regression. Injury. 2015;46(6):954-962. doi:10.1016/j.injury.2015.03.024
32. Gyawali B, Shimokata T, Ando M, Honda K, Ando Y. Risk of serious adverse events and fatal adverse events with sorafenib in patients with solid cancer: a meta-analysis of phase 3 randomized controlled trials†. Ann Oncol. 2017;28(2):246-253. doi:10.1093/annonc/mdw549
33. Gunter BR, Butler KA, Wallace RL, Smith SM, Harirforoosh S. Non-steroidal anti-inflammatory drug-induced cardiovascular adverse events: a meta-analysis. J Clin Pharm Ther. 2017;42(1):27-38. doi:10.1111/jcpt.12484
34. Li BD, Bi ZY, Liu JF, et al. Adverse effects produced by different drugs used in the treatment of Parkinson's disease: A mixed treatment comparison. CNS Neurosci Ther. 2017;23(10):827-842. doi:10.1111/cns.12727
35. Alfageh BH, Wang Z, Mongkhon P, et al. Safety and Tolerability of Antipsychotic Medication in Individuals with Autism Spectrum Disorder: A Systematic Review and Meta-Analysis. Paediatr Drugs. 2019;21(3):153-167. doi:10.1007/s40272-019-00333-x
36. Bell S, Rennie T, Marwick CA, Davey P. Effects of peri-operative nonsteroidal anti-inflammatory drugs on post-operative kidney function for adults with normal kidney function. Cochrane Database Syst Rev. 2018;11(11):CD011274. Published 2018 Nov 29. doi:10.1002/14651858.CD011274.pub2
37. Castagné B, Viprey M, Martin J, Schott AM, Cucherat M, Soubrier M. Cardiovascular safety of tocilizumab: A systematic review and network meta-analysis. PLoS One. 2019;14(8):e0220178. Published 2019 Aug 1. doi:10.1371/journal.pone.0220178
38. Gu B, Gao W, Chu H, et al. Adverse events risk associated with anti-VEGFR agents in the treatment of advanced nonsmall-cell lung cancer: A meta-analysis. Medicine (Baltimore). 2016;95(48):e3752. doi:10.1097/MD.0000000000003752
39. Biardeau X, Zanaty M, Aoun F, Benbouzid S, Peyronnet B. Voies d'abord et complications des bandelettes sous-urétrales synthétiques chez la femme : revue systématique de la littérature et méta-analyse [Approach and complications associated with suburethral synthetic slings in women: Systematic review and meta-analysis]. Prog Urol. 2016;26(4):254-269. doi:10.1016/j.purol.2015.08.314
40. Tang B, Wang J, Luo LL, Li QG, Huang D. Risks of budesonide/formoterol for the treatment of stable COPD: a meta-analysis. Int J Chron Obstruct Pulmon Dis. 2019;14:757-766. Published 2019 Apr 1. doi:10.2147/COPD.S192166
41. Zhao B, Zhao H, Zhao J. Risk of fatal adverse events in cancer patients treated with sunitinib. Crit Rev Oncol Hematol. 2019;137:115-122. doi:10.1016/j.critrevonc.2019.03.007
42. Liu B, Ding F, Liu Y, et al. Incidence and risk of hypertension associated with vascular endothelial growth factor receptor tyrosine kinase inhibitors in cancer patients: a comprehensive network meta-analysis of 72 randomized controlled trials involving 30013 patients. Oncotarget. 2016;7(41):67661-67673. doi:10.18632/oncotarget.11813
43. Zhang B, Wu Q, Zhou YL, Guo X, Ge J, Fu J. Immune-related adverse events from combination immunotherapy in cancer patients: A comprehensive meta-analysis of randomized controlled trials. Int Immunopharmacol. 2018;63:292-298. doi:10.1016/j.intimp.2018.08.014
44. Botero Aguirre JP, Restrepo Hamid AM. Amphotericin B deoxycholate versus liposomal amphotericin B: effects on kidney function. Cochrane Database Syst Rev. 2015;(11):CD010481. Published 2015 Nov 23. doi:10.1002/14651858.CD010481.pub2
45. Morris BJ, Krieger JN. Does Circumcision Increase Meatal Stenosis Risk?-A Systematic Review and Meta-analysis. Urology. 2017;110:16-26. doi:10.1016/j.urology.2017.07.027
46. Galling B, Roldán A, Rietschel L, et al. Safety and tolerability of antipsychotic co-treatment in patients with schizophrenia: results from a systematic review and meta-analysis of randomized controlled trials. Expert Opin Drug Saf. 2016;15(5):591-612. doi:10.1517/14740338.2016.1165668
47. Raccah BH, Perlman A, Danenberg HD, Pollak A, Muszkat M, Matok I. Major Bleeding and Hemorrhagic Stroke With Direct Oral Anticoagulants in Patients With Renal Failure: Systematic Review and Meta-Analysis of Randomized Trials. Chest. 2016;149(6):1516-1524. doi:10.1016/j.chest.2015.12.029
48. Yoon BH, Ha YC, Lee YK, Koo KH. Postoperative Deep Infection After Cemented Versus Cementless Total Hip Arthroplasty: A Meta-Analysis. J Arthroplasty. 2015;30(10):1823-1827. doi:10.1016/j.arth.2015.04.041
49. Yoon BH, Seo JG, Koo KH. Comparison of Postoperative Infection-Related Complications between Cemented and Cementless Hemiarthroplasty in Elderly Patients: A Meta-Analysis. Clin Orthop Surg. 2017;9(2):145-152. doi:10.4055/cios.2017.9.2.145
50. Diener C, Horneff G. Comparison of adverse events of biologicals for treatment of juvenile idiopathic arthritis: a systematic review. Expert Opin Drug Saf. 2019;18(8):719-732. doi:10.1080/14740338.2019.1632288
51. Alves C, Ribeiro I, Penedones A, Mendes D, Batel Marques F. Risk of Ophthalmic Adverse Effects in Patients Treated with MEK Inhibitors: A Systematic Review and Meta-Analysis. Ophthalmic Res. 2017;57(1):60-69. doi:10.1159/000446845
52. Cates CJ, Schmidt S, Ferrer M, Sayer B, Waterson S. Inhaled steroids with and without regular salmeterol for asthma: serious adverse events. Cochrane Database Syst Rev. 2018;12(12):CD006922. Published 2018 Dec 3. doi:10.1002/14651858.CD006922.pub4
53. Ching C, Eslick GD, Poulton AS. Evaluation of Methylphenidate Safety and Maximum-Dose Titration Rationale in Attention-Deficit/Hyperactivity Disorder: A Meta-analysis. JAMA Pediatr. 2019;173(7):630-639. doi:10.1001/jamapediatrics.2019.0905
54. Qu CP, Sun GX, Yang SQ, Tian J, Si JG, Wang YF. Toxicities of different first-line chemotherapy regimens in the treatment of advanced ovarian cancer: A network meta-analysis. Medicine (Baltimore). 2017;96(2):e5797. doi:10.1097/MD.0000000000005797
55. Peng C, Ling Y, Ma C, et al. Safety Outcomes of NOTES Cholecystectomy Versus Laparoscopic Cholecystectomy: A Systematic Review and Meta-Analysis. Surg Laparosc Endosc Percutan Tech. 2016;26(5):347-353. doi:10.1097/SLE.0000000000000284
56. Xu C, Chen YP, Du XJ, et al. Comparative safety of immune checkpoint inhibitors in cancer: systematic review and network meta-analysis. BMJ. 2018;363:k4226. Published 2018 Nov 8. doi:10.1136/bmj.k4226
57. Chang CH, Tseng PT, Chen NY, et al. Safety and tolerability of prescription omega-3 fatty acids: A systematic review and meta-analysis of randomized controlled trials. Prostaglandins Leukot Essent Fatty Acids. 2018;129:1-12. doi:10.1016/j.plefa.2018.01.001
58. Du CR, Ying HM, Kong FF, Zhai RP, Hu CS. Concurrent chemoradiotherapy was associated with a higher severe late toxicity rate in nasopharyngeal carcinoma patients compared with radiotherapy alone: a meta-analysis based on randomized controlled trials. Radiat Oncol. 2015;10:70. Published 2015 Mar 26. doi:10.1186/s13014-015-0377-9
59. Li C, Cheng W, Guo J, Guan W. Relationship of inhaled long-acting bronchodilators with cardiovascular outcomes among patients with stable COPD: a meta-analysis and systematic review of 43 randomized trials. Int J Chron Obstruct Pulmon Dis. 2019;14:799-808. Published 2019 Apr 11. doi:10.2147/COPD.S198288
60. Sathya C, Wayne C, Gotsch A, Vincent J, Sullivan KJ, Nasr A. Laparoscopic versus open pyloromyotomy in infants: a systematic review and meta-analysis. Pediatr Surg Int. 2017;33(3):325-333. doi:10.1007/s00383-016-4030-y
61. Chou CY, Chang YT, Yang JL, et al. Effect of Long-term Incretin-Based Therapies on Ischemic Heart Diseases in Patients with Type 2 Diabetes Mellitus: A Network Meta-analysis. Sci Rep. 2017;7(1):15795. Published 2017 Nov 17. doi:10.1038/s41598-017-16101-1
62. Chi CC, Wang SH, Wojnarowska F, Kirtschig G, Davies E, Bennett C. Safety of topical corticosteroids in pregnancy. Cochrane Database Syst Rev. 2015;(10):CD007346. Published 2015 Oct 26. doi:10.1002/14651858.CD007346.pub3
63. Ciccarese C, Iacovelli R, Bria E, et al. The incidence and relative risk of pulmonary toxicity in patients treated with anti-PD1/PD-L1 therapy for solid tumors: a meta-analysis of current studies. Immunotherapy. 2017;9(7):579-587. doi:10.2217/imt-2017-0018
64. You CH, Lin CK, Chen PH, et al. Clarithromycin use and the risk of mortality and cardiovascular events: A systematic review and meta-analysis. PLoS One. 2019;14(12):e0226637. Published 2019 Dec 27. doi:10.1371/journal.pone.0226637
65. Gao C, Wang J, Li Y, et al. Incidence and risk of hematologic toxicities with hypomethylating agents in the treatment of myelodysplastic syndromes and acute myeloid leukopenia: A systematic review and meta-analysis. Medicine (Baltimore). 2018;97(34):e11860. doi:10.1097/MD.0000000000011860
66. Soo CG, Della Torre PK, Yolland TJ, Shatwell MA. Clopidogrel and hip fractures, is it safe? A systematic review and meta-analysis. BMC Musculoskelet Disord. 2016;17:136. Published 2016 Mar 22. doi:10.1186/s12891-016-0988-9
67. Ma C, Panaccione NR, Nguyen TM, et al. Adverse Events and Nocebo Effects in Inflammatory Bowel Disease: A Systematic Review and Meta-Analysis of Randomized Controlled Trials. J Crohns Colitis. 2019;13(9):1201-1216. doi:10.1093/ecco-jcc/jjz087
68. Uhlig C, Bluth T, Schwarz K, et al. Effects of Volatile Anesthetics on Mortality and Postoperative Pulmonary and Other Complications in Patients Undergoing Surgery: A Systematic Review and Meta-analysis. Anesthesiology. 2016;124(6):1230-1245. doi:10.1097/ALN.0000000000001120
69. Maniotis C, Andreou C, Karalis I, Koutouzi G, Agelaki M, Koutouzis M. A systematic review on the safety of Prostar XL versus ProGlide after TAVR and EVAR. Cardiovasc Revasc Med. 2017;18(2):145-150. doi:10.1016/j.carrev.2016.11.004
70. Wang C, Wang F, Min X, et al. Toxicities of chemoradiotherapy and radiotherapy in nasopharyngeal carcinoma: an updated meta-analysis. J Int Med Res. 2019;47(7):2832-2847. doi:10.1177/0300060519858031
71. Yang C, Yi Q, Zhang L, Cui H, Mao J. Safety of aripiprazole for tics in children and adolescents: A systematic review and meta-analysis. Medicine (Baltimore). 2019;98(22):e15816. doi:10.1097/MD.0000000000015816
72. Thomopoulos C, Parati G, Zanchetti A. Effects of blood-pressure-lowering treatment in hypertension: 9. Discontinuations for adverse events attributed to different classes of antihypertensive drugs: meta-analyses of randomized trials. J Hypertens. 2016;34(10):1921-1932. doi:10.1097/HJH.0000000000001052
73. Li D, Yang JY, Wang T, Shen S, Tang H. Risks of diabetic foot syndrome and amputation associated with sodium glucose co-transporter 2 inhibitors: A Meta-analysis of Randomized Controlled Trials. Diabetes Metab. 2018;44(5):410-414. doi:10.1016/j.diabet.2018.02.001
74. Kim DH, Rogers JR, Fulchino LA, Kim CA, Solomon DH, Kim SC. Bisphosphonates and risk of cardiovascular events: a meta-analysis. PLoS One. 2015;10(4):e0122646. Published 2015 Apr 17. doi:10.1371/journal.pone.0122646
75. Yahav D, Green H, Eliakim-Raz N, Mor E, Husain S. Early double J stent removal in renal transplant patients to prevent urinary tract infection - systematic review and meta-analysis of randomized controlled trials. Eur J Clin Microbiol Infect Dis. 2018;37(4):773-778. doi:10.1007/s10096-017-3173-7
76. Caldeira D, Barra M, Ferreira A, et al. Systematic review with meta-analysis: the risk of major gastrointestinal bleeding with non-vitamin K antagonist oral anticoagulants. Aliment Pharmacol Ther. 2015;42(11-12):1239-1249. doi:10.1111/apt.13412
77. Caldeira D, Barra M, Santos AT, de Abreu D, Costa J, Ferreira JJ. Risk of insomnia with non-vitamin K oral anticoagulants: systematic review and meta-analysis. Sleep Breath. 2015;19(3):1043-1049. doi:10.1007/s11325-014-1112-8
78. Caldeira D, Canastro M, Barra M, et al. Risk of Substantial Intraocular Bleeding With Novel Oral Anticoagulants: Systematic Review and Meta-analysis. JAMA Ophthalmol. 2015;133(7):834-839. doi:10.1001/jamaophthalmol.2015.0985
79. Caldeira D, Gonçalves N, Pinto FJ, Costa J, Ferreira JJ. Risk of renal failure with the non-vitamin K antagonist oral anticoagulants: systematic review and meta-analysis. Pharmacoepidemiol Drug Saf. 2015;24(7):757-764. doi:10.1002/pds.3791
80. Caldeira D, Rodrigues FB, Barra M, et al. Non-vitamin K antagonist oral anticoagulants and major bleeding-related fatality in patients with atrial fibrillation and venous thromboembolism: a systematic review and meta-analysis. Heart. 2015;101(15):1204-1211. doi:10.1136/heartjnl-2015-307489
81. Caldeira D, Ferreira JJ, Pinto FJ, Costa J. Safety of non-vitamin K antagonist oral anticoagulants - coronary risks. Expert Opin Drug Saf. 2016;15(6):731-740. doi:10.1517/14740338.2016.1164689
82. Caldeira D, Rodrigues FB, Duarte MM, et al. Cardiac Harms of Sofosbuvir: Systematic Review and Meta-Analysis. Drug Saf. 2018;41(1):77-86. doi:10.1007/s40264-017-0586-2
83. Caldeira D, Alves D, Costa J, Ferreira JJ, Pinto FJ. Ibrutinib increases the risk of hypertension and atrial fibrillation: Systematic review and meta-analysis. PLoS One. 2019;14(2):e0211228. Published 2019 Feb 20. doi:10.1371/journal.pone.0211228
84. Giacoppo D, Colleran R, Cassese S, et al. Percutaneous Coronary Intervention vs Coronary Artery Bypass Grafting in Patients With Left Main Coronary Artery Stenosis: A Systematic Review and Meta-analysis. JAMA Cardiol. 2017;2(10):1079-1088. doi:10.1001/jamacardio.2017.2895
85. Chierrito de Oliveira D, Guerrero de Sousa P, Borges Dos Reis C, et al. Safety of Treatments for ADHD in Adults: Pairwise and Network Meta-Analyses. J Atten Disord. 2019;23(2):111-120. doi:10.1177/1087054717696773
86. Giugliano D, Maiorino MI, Longo M, Bellastella G, Chiodini P, Esposito K. Type 2 diabetes and risk of heart failure: a systematic review and meta-analysis from cardiovascular outcome trials. Endocrine. 2019;65(1):15-24. doi:10.1007/s12020-019-01931-y
87. Vukadinović D, Scholz SS, Messerli FH, et al. Peripheral edema and headache associated with amlodipine treatment: a meta-analysis of randomized, placebo-controlled trials. J Hypertens. 2019;37(10):2093-2103. doi:10.1097/HJH.0000000000002145
88. Cheng D, Gao H, Li W. Long-term risk of rosiglitazone on cardiovascular events - a systematic review and meta-analysis. Endokrynol Pol. 2018;69(4):381-394. doi:10.5603/EP.a2018.0036
89. Li DF, Yang MF, Chang X, et al. Endocut Versus Conventional Blended Electrosurgical Current for Endoscopic Biliary Sphincterotomy: A Meta-Analysis of Complications. Dig Dis Sci. 2019;64(8):2088-2094. doi:10.1007/s10620-019-05513-w
90. Demiri M, Antunes T, Fletcher D, Martinez V. Perioperative adverse events attributed to α2-adrenoceptor agonists in patients not at risk of cardiovascular events: systematic review and meta-analysis. Br J Anaesth. 2019;123(6):795-807. doi:10.1016/j.bja.2019.07.029
91. Sobieraj DM, Martinez BK, Hernandez AV, et al. Adverse Effects of Pharmacologic Treatments of Major Depression in Older Adults. J Am Geriatr Soc. 2019;67(8):1571-1581. doi:10.1111/jgs.15966
92. Varvaki Rados D, Catani Pinto L, Reck Remonti L, Bauermann Leitão C, Gross JL. The Association between Sulfonylurea Use and All-Cause and Cardiovascular Mortality: A Meta-Analysis with Trial Sequential Analysis of Randomized Clinical Trials. PLoS Med. 2016;13(4):e1001992. Published 2016 Apr 12. doi:10.1371/journal.pmed.1001992
93. Mohananey D, Sengodan P, Banerjee K, et al. Comparative analysis of cerebrovascular events in transcatheter and surgical aortic valve replacement: a systematic review and meta-analysis of randomised trials. EuroIntervention. 2018;14(1):69-77. doi:10.4244/EIJ-D-17-00732
94. Hong D, Zhang G, Zhang X, Lian X. Pulmonary Toxicities of Gefitinib in Patients With Advanced Non-Small-Cell Lung Cancer: A Meta-Analysis of Randomized Controlled Trials [published correction appears in Medicine (Baltimore). 2016 Jul 29;95(30):e156a]. Medicine (Baltimore). 2016;95(9):e3008. doi:10.1097/MD.0000000000003008
95. Mannucci E, Monami M. Cardiovascular Safety of Incretin-Based Therapies in Type 2 Diabetes: Systematic Review of Integrated Analyses and Randomized Controlled Trials. Adv Ther. 2017;34(1):1-40. doi:10.1007/s12325-016-0432-4
96. Zabaleta-Del-Olmo E, Vlacho B, Jodar-Fernández L, et al. Safety of the reuse of needles for subcutaneous insulin injection: A systematic review and meta-analysis. Int J Nurs Stud. 2016;60:121-132. doi:10.1016/j.ijnurstu.2016.04.010
97. Gorelik E, Masarwa R, Perlman A, et al. Fluoroquinolones and Cardiovascular Risk: A Systematic Review, Meta-analysis and Network Meta-analysis. Drug Saf. 2019;42(4):529-538. doi:10.1007/s40264-018-0751-2
98. Esaiassen E, Fjalstad JW, Juvet LK, van den Anker JN, Klingenberg C. Antibiotic exposure in neonates and early adverse outcomes: a systematic review and meta-analysis. J Antimicrob Chemother. 2017;72(7):1858-1870. doi:10.1093/jac/dkx088
99. Karyotaki E, Kemmeren L, Riper H, et al. Is self-guided internet-based cognitive behavioural therapy (iCBT) harmful? An individual participant data meta-analysis. Psychol Med. 2018;48(15):2456-2466. doi:10.1017/S0033291718000648
100. Elgendy IY, Mahmoud AN, Brilakis ES, Bavry AA. Drug-eluting stents versus bare metal stents for saphenous vein graft revascularisation: a meta-analysis of randomised trials. EuroIntervention. 2018;14(2):215-223. doi:10.4244/EIJ-D-17-00839
101. Penninga EI, Graudal N, Ladekarl MB, Jürgens G. Adverse Events Associated with Flumazenil Treatment for the Management of Suspected Benzodiazepine Intoxication--A Systematic Review with Meta-Analyses of Randomised Trials. Basic Clin Pharmacol Toxicol. 2016;118(1):37-44. doi:10.1111/bcpt.12434
102. Curtis E, Fuggle N, Shaw S, et al. Safety of Cyclooxygenase-2 Inhibitors in Osteoarthritis: Outcomes of a Systematic Review and Meta-Analysis. Drugs Aging. 2019;36(Suppl 1):25-44. doi:10.1007/s40266-019-00664-x
103. Osland E, Yunus RM, Khan S, Memon B, Memon MA. Late Postoperative Complications in Laparoscopic Sleeve Gastrectomy (LVSG) Versus Laparoscopic Roux-en-y Gastric Bypass (LRYGB): Meta-analysis and Systematic Review. Surg Laparosc Endosc Percutan Tech. 2016;26(3):193-201. doi:10.1097/SLE.0000000000000279
104. Barzilay E, Gadot Y, Koren G. Safety of vaginal delivery in very low birthweight vertex singletons: a meta-analysis. J Matern Fetal Neonatal Med. 2016;29(22):3724-3729. doi:10.3109/14767058.2016.1141889
105. Shah ED, Farida JP, Siegel CA, Chong K, Melmed GY. Risk for Overall Infection with Anti-TNF and Anti-integrin Agents Used in IBD: A Systematic Review and Meta-analysis. Inflamm Bowel Dis. 2017;23(4):570-577. doi:10.1097/MIB.0000000000001049
106. Ammann EM, Haskins CB, Fillman KM, et al. Intravenous immune globulin and thromboembolic adverse events: A systematic review and meta-analysis of RCTs. Am J Hematol. 2016;91(6):594-605. doi:10.1002/ajh.24358
107. Balk EM, Earley A, Avendano EA, Raman G. Long-Term Health Outcomes in Women With Silicone Gel Breast Implants: A Systematic Review. Ann Intern Med. 2016;164(3):164-175. doi:10.7326/M15-1169
108. Whitlock EP, Burda BU, Williams SB, Guirguis-Blake JM, Evans CV. Bleeding Risks With Aspirin Use for Primary Prevention in Adults: A Systematic Review for the U.S. Preventive Services Task Force. Ann Intern Med. 2016;164(12):826-835. doi:10.7326/M15-2112
109. Khosrow-Khavar F, Filion KB, Al-Qurashi S, et al. Cardiotoxicity of aromatase inhibitors and tamoxifen in postmenopausal women with breast cancer: a systematic review and meta-analysis of randomized controlled trials. Ann Oncol. 2017;28(3):487-496. doi:10.1093/annonc/mdw673
110. Aljebab F, Choonara I, Conroy S. Systematic review of the toxicity of short-course oral corticosteroids in children. Arch Dis Child. 2016;101(4):365-370. doi:10.1136/archdischild-2015-309522
111. Aljebab F, Choonara I, Conroy S. Systematic Review of the Toxicity of Long-Course Oral Corticosteroids in Children. PLoS One. 2017;12(1):e0170259. Published 2017 Jan 26. doi:10.1371/journal.pone.0170259
112. Farah D, Leme GM, Eliaschewitz FG, Fonseca MCM. A safety and tolerability profile comparison between dipeptidyl peptidase-4 inhibitors and sulfonylureas in diabetic patients: A systematic review and meta-analysis. Diabetes Res Clin Pract. 2019;149:47-63. doi:10.1016/j.diabres.2019.01.025
113. Petrelli F, Ardito R, Ghidini A, et al. Different Toxicity of Cetuximab and Panitumumab in Metastatic Colorectal Cancer Treatment: A Systematic Review and Meta-Analysis. Oncology. 2018;94(4):191-199. doi:10.1159/000486338
114. Fogacci F, Banach M, Mikhailidis DP, et al. Safety of red yeast rice supplementation: A systematic review and meta-analysis of randomized controlled trials. Pharmacol Res. 2019;143:1-16. doi:10.1016/j.phrs.2019.02.028
115. Lussana F, Cattaneo M, Rambaldi A, Squizzato A. Ruxolitinib-associated infections: A systematic review and meta-analysis. Am J Hematol. 2018;93(3):339-347. doi:10.1002/ajh.24976
116. Zhao F, Lei R, Yang SK, et al. Comparative effect of iso-osmolar versus low-osmolar contrast media on the incidence of contrast-induced acute kidney injury in diabetic patients: a systematic review and meta-analysis. Cancer Imaging. 2019;19(1):38. Published 2019 Jun 18. doi:10.1186/s40644-019-0224-6
117. Aires FT, Dedivitis RA, Petrarolha SM, Bernardo WM, Cernea CR, Brandão LG. Early oral feeding after total laryngectomy: A systematic review. Head Neck. 2015;37(10):1532-1535. doi:10.1002/hed.23755
118. Huang F, Luo ZC. Risk of Adverse Drug Events Observed with Baricitinib 2 mg Versus Baricitinib 4 mg Once Daily for the Treatment of Rheumatoid Arthritis: A Systematic Review and Meta-Analysis of Randomized Controlled Trials. BioDrugs. 2018;32(5):415-423. doi:10.1007/s40259-018-0304-3
119. Huang F, Luo ZC. Adverse drug events associated with 5mg versus 10mg Tofacitinib (Janus kinase inhibitor) twice daily for the treatment of autoimmune diseases: A systematic review and meta-analysis of randomized controlled trials. Clin Rheumatol. 2019;38(2):523-534. doi:10.1007/s10067-018-4299-4
120. Wang FB, Pu YW, Zhong FY, Lv XD, Yang ZX, Xing CG. Laparoscopic permanent sigmoid stoma creation through the extraperitoneal route versus transperitoneal route. A meta-analysis of stoma-related complications. Saudi Med J. 2015;36(2):159-163. doi:10.15537/smj.2015.2.10203
121. Chen F, Pu F. Safety of Denosumab Versus Zoledronic Acid in Patients with Bone Metastases: A Meta-Analysis of Randomized Controlled Trials. Oncol Res Treat. 2016;39(7-8):453-459. doi:10.1159/000447372
122. Catalá-López F, Corrales I, de la Fuente-Honrubia C, et al. Risk of thromboembolism with thrombopoietin receptor agonists in adult patients with thrombocytopenia: Systematic review and meta-analysis of randomized controlled trials. Med Clin (Barc). 2015;145(12):511-519. doi:10.1016/j.medcli.2015.03.014
123. Sandberg F, Viktorsdóttir MB, Salö M, Stenström P, Arnbjörnsson E. Comparison of major complications in children after laparoscopy-assisted gastrostomy and percutaneous endoscopic gastrostomy placement: a meta-analysis. Pediatr Surg Int. 2018;34(12):1321-1327. doi:10.1007/s00383-018-4358-6
124. Zagmutt FJ, Carroll CA. Meta-analysis of adverse events in recent randomized clinical trials for dimethyl fumarate, glatiramer acetate and teriflunomide for the treatment of relapsing forms of multiple sclerosis. Int J Neurosci. 2015;125(11):798-807. doi:10.3109/00207454.2014.979982
125. Hovaguimian F, Köppel S, Spahn DR. Safety of Anticoagulation Interruption in Patients Undergoing Surgery or Invasive Procedures: A Systematic Review and Meta-analyses of Randomized Controlled Trials and Non-randomized Studies. World J Surg. 2017;41(10):2444-2456. doi:10.1007/s00268-017-4072-x
126. Misawa F, Kishimoto T, Hagi K, Kane JM, Correll CU. Safety and tolerability of long-acting injectable versus oral antipsychotics: A meta-analysis of randomized controlled studies comparing the same antipsychotics. Schizophr Res. 2016;176(2-3):220-230. doi:10.1016/j.schres.2016.07.018
127. Roberto G, Raschi E, Piccinni C, et al. Adverse cardiovascular events associated with triptans and ergotamines for treatment of migraine: systematic review of observational studies. Cephalalgia. 2015;35(2):118-131. doi:10.1177/0333102414550416
128. Corona G, Tirabassi G, Santi D, et al. Sexual dysfunction in subjects treated with inhibitors of 5α-reductase for benign prostatic hyperplasia: a comprehensive review and meta-analysis. Andrology. 2017;5(4):671-678. doi:10.1111/andr.12353
129. Geminiani A, Tsigarida A, Chochlidakis K, Papaspyridakos PV, Feng C, Ercoli C. A meta-analysis of complications during sinus augmentation procedure. Quintessence Int. 2017;48(3):231-240. doi:10.3290/j.qi.a37644
130. Vaos G, Dimopoulou A, Gkioka E, Zavras N. Immediate surgery or conservative treatment for complicated acute appendicitis in children? A meta-analysis. J Pediatr Surg. 2019;54(7):1365-1371. doi:10.1016/j.jpedsurg.2018.07.017
131. Honvo G, Leclercq V, Geerinck A, et al. Safety of Topical Non-steroidal Anti-Inflammatory Drugs in Osteoarthritis: Outcomes of a Systematic Review and Meta-Analysis. Drugs Aging. 2019;36(Suppl 1):45-64. doi:10.1007/s40266-019-00661-0
132. Honvo G, Reginster JY, Rabenda V, et al. Safety of Symptomatic Slow-Acting Drugs for Osteoarthritis: Outcomes of a Systematic Review and Meta-Analysis. Drugs Aging. 2019;36(Suppl 1):65-99. doi:10.1007/s40266-019-00662-z
133. Honvo G, Reginster JY, Rannou F, et al. Safety of Intra-articular Hyaluronic Acid Injections in Osteoarthritis: Outcomes of a Systematic Review and Meta-Analysis. Drugs Aging. 2019;36(Suppl 1):101-127. doi:10.1007/s40266-019-00657-w
134. Roviello G, Sigala S, Danesi R, et al. Incidence and relative risk of adverse events of special interest in patients with castration resistant prostate cancer treated with CYP-17 inhibitors: A meta-analysis of published trials. Crit Rev Oncol Hematol. 2016;101:12-20. doi:10.1016/j.critrevonc.2016.02.013
135. Roviello G, Generali D. Is the fatigue an adverse event of the second generation of hormonal therapy? Data from a literature-based meta-analysis. Med Oncol. 2018;35(3):29. Published 2018 Jan 31. doi:10.1007/s12032-018-1081-z
136. Boleto G, Kanagaratnam L, Dramé M, Salmon JH. Safety of combination therapy with two bDMARDs in patients with rheumatoid arthritis: A systematic review and meta-analysis. Semin Arthritis Rheum. 2019;49(1):35-42. doi:10.1016/j.semarthrit.2018.12.003
137. Duarte GS, Rodrigues FB, Ferreira JJ, Costa J. Adverse events with botulinum toxin treatment in cervical dystonia: How much should we blame placebo?. Parkinsonism Relat Disord. 2018;56:16-19. doi:10.1016/j.parkreldis.2018.06.017
138. Duarte GS, Nunes-Ferreira A, Rodrigues FB, et al. Morphine in acute coronary syndrome: systematic review and meta-analysis. BMJ Open. 2019;9(3):e025232. Published 2019 Mar 15. doi:10.1136/bmjopen-2018-025232
139. Beger HG, Siech M, Poch B, Mayer B, Schoenberg MH. Limited surgery for benign tumours of the pancreas: a systematic review. World J Surg. 2015;39(6):1557-1566. doi:10.1007/s00268-015-2976-x
140. Tang HL, Li DD, Zhang JJ, et al. Lack of evidence for a harmful effect of sodium-glucose co-transporter 2 (SGLT2) inhibitors on fracture risk among type 2 diabetes patients: a network and cumulative meta-analysis of randomized controlled trials. Diabetes Obes Metab. 2016;18(12):1199-1206. doi:10.1111/dom.12742
141. Boltman-Binkowski H. A systematic review: Are herbal and homeopathic remedies used during pregnancy safe?. Curationis. 2016;39(1):1514. Published 2016 Apr 13. doi:10.4102/curationis.v39i1.1514
142. Li H, Wang C, Zhang S, et al. Safety Profile of Atorvastatin 80 mg: A Meta-Analysis of 17 Randomized Controlled Trials in 21,910 Participants. Drug Saf. 2016;39(5):409-419. doi:10.1007/s40264-016-0394-0
143. Xu H, Tan P, Zheng X, et al. Immune-related adverse events following administration of anti-cytotoxic T-lymphocyte-associated protein-4 drugs: a comprehensive systematic review and meta-analysis. Drug Des Devel Ther. 2019;13:2215-2234. Published 2019 Jul 4. doi:10.2147/DDDT.S196316
144. Dinges HC, Otto S, Stay DK, et al. Side Effect Rates of Opioids in Equianalgesic Doses via Intravenous Patient-Controlled Analgesia: A Systematic Review and Network Meta-analysis. Anesth Analg. 2019;129(4):1153-1162. doi:10.1213/ANE.0000000000003887
145. Winberg H, Arnbjörnsson E, Anderberg M, Stenström P. Postoperative outcomes in distal hypospadias: a meta-analysis of the Mathieu and tubularized incised plate repair methods for development of urethrocutaneous fistula and urethral stricture. Pediatr Surg Int. 2019;35(11):1301-1308. doi:10.1007/s00383-019-04523-z
146. Hansen MP, Scott AM, McCullough A, et al. Adverse events in people taking macrolide antibiotics versus placebo for any indication. Cochrane Database Syst Rev. 2019;1(1):CD011825. Published 2019 Jan 18. doi:10.1002/14651858.CD011825.pub2
147. Storgaard H, Cold F, Gluud LL, Vilsbøll T, Knop FK. Glucagon-like peptide-1 receptor agonists and risk of acute pancreatitis in patients with type 2 diabetes. Diabetes Obes Metab. 2017;19(6):906-908. doi:10.1111/dom.12885
148. Shang H, Zhang Z, Feng A, et al. The overall safety evaluation of programmed cell death/programmed cell death ligand 1 (PD-1/PD-L1) treatment for lung cancer patients: An updated systematic review and meta-analysis. Medicine (Baltimore). 2019;98(30):e16439. doi:10.1097/MD.0000000000016439
149. Fan H, Li L, Wijlaars L, Gilbert RE. Associations between use of macrolide antibiotics during pregnancy and adverse child outcomes: A systematic review and meta-analysis. PLoS One. 2019;14(2):e0212212. Published 2019 Feb 19. doi:10.1371/journal.pone.0212212
150. Leung HW, Chan AL. Trastuzumab-induced cardiotoxicity in elderly women with HER-2-positive breast cancer: a meta-analysis of real-world data. Expert Opin Drug Saf. 2015;14(11):1661-1671. doi:10.1517/14740338.2015.1089231
151. Cramer H, Ward L, Saper R, Fishbein D, Dobos G, Lauche R. The Safety of Yoga: A Systematic Review and Meta-Analysis of Randomized Controlled Trials. Am J Epidemiol. 2015;182(4):281-293. doi:10.1093/aje/kwv071
152. Geng HZ, Nasier D, Liu B, Gao H, Xu YK. Meta-analysis of elective surgical complications related to defunctioning loop ileostomy compared with loop colostomy after low anterior resection for rectal carcinoma. Ann R Coll Surg Engl. 2015;97(7):494-501. doi:10.1308/003588415X14181254789240
153. Zhang H, Huang Z, Zou X, Liu T. Bevacizumab and wound-healing complications: a systematic review and meta-analysis of randomized controlled trials. Oncotarget. 2016;7(50):82473-82481. doi:10.18632/oncotarget.12666
154. Tong H, Zhu Y, Liu Y. Incidence and risk of fatigue in cancer patients treated with MET inhibitors: A systematic review and meta-analysis. Medicine (Baltimore). 2019;98(22):e15522. doi:10.1097/MD.0000000000015522
155. Ma H, Liu Y, Huang L, et al. The Adverse Events of Oxycodone in Cancer-Related Pain: A Systematic Review and Meta-Analysis of Randomized Controlled Trials. Medicine (Baltimore). 2016;95(15):e3341. doi:10.1097/MD.0000000000003341
156. Tang H, Fang Z, Wang T, Cui W, Zhai S, Song Y. Meta-Analysis of Effects of Sodium-Glucose Cotransporter 2 Inhibitors on Cardiovascular Outcomes and All-Cause Mortality Among Patients With Type 2 Diabetes Mellitus. Am J Cardiol. 2016;118(11):1774-1780. doi:10.1016/j.amjcard.2016.08.061
157. Tang H, Li D, Zhang J, et al. Sodium-glucose co-transporter-2 inhibitors and risk of adverse renal outcomes among patients with type 2 diabetes: A network and cumulative meta-analysis of randomized controlled trials. Diabetes Obes Metab. 2017;19(8):1106-1115. doi:10.1111/dom.12917
158. Abdel-Qadir H, Ethier JL, Lee DS, Thavendiranathan P, Amir E. Cardiovascular toxicity of angiogenesis inhibitors in treatment of malignancy: A systematic review and meta-analysis. Cancer Treat Rev. 2017;53:120-127. doi:10.1016/j.ctrv.2016.12.002
159. Tanboğa İH, Topçu S, Aksakal E, et al. The Risk of Atrial Fibrillation With Ivabradine Treatment: A Meta-analysis With Trial Sequential Analysis of More Than 40000 Patients. Clin Cardiol. 2016;39(10):615-620. doi:10.1002/clc.22578
160. Carvalho ÍT, Baccaglini W, Claros OR, et al. Genitourinary and gastrointestinal toxicity among patients with localized prostate cancer treated with conventional versus moderately hypofractionated radiation therapy: systematic review and meta-analysis. Acta Oncol. 2018;57(8):1003-1010. doi:10.1080/0284186X.2018.1478126
161. Dicembrini I, Tomberli B, Nreu B, et al. Peripheral artery disease and amputations with Sodium-Glucose co-Transporter-2 (SGLT-2) inhibitors: A meta-analysis of randomized controlled trials. Diabetes Res Clin Pract. 2019;153:138-144. doi:10.1016/j.diabres.2019.05.028
162. Hougen I, Collister D, Bourrier M, et al. Safety of Intravenous Iron in Dialysis: A Systematic Review and Meta-Analysis. Clin J Am Soc Nephrol. 2018;13(3):457-467. doi:10.2215/CJN.05390517
163. Andreou I, Briasoulis A, Pappas C, Ikonomidis I, Alexopoulos D. Ticagrelor Versus Clopidogrel as Part of Dual or Triple Antithrombotic Therapy: a Systematic Review and Meta-Analysis. Cardiovasc Drugs Ther. 2018;32(3):287-294. doi:10.1007/s10557-018-6795-9
164. Balasubramanian I, Fleming C, Mohan HM, Schmidt K, Haglind E, Winter DC. Out-Patient Management of Mild or Uncomplicated Diverticulitis: A Systematic Review. Dig Surg. 2017;34(2):151-160. doi:10.1159/000450865
165. Elgendy IY, Mahmoud AN, Barakat AF, et al. Cardiovascular Safety of Dipeptidyl-Peptidase IV Inhibitors: A Meta-Analysis of Placebo-Controlled Randomized Trials. Am J Cardiovasc Drugs. 2017;17(2):143-155. doi:10.1007/s40256-016-0208-x
166. Cavero-Redondo I, Álvarez-Bueno C, Pozuelo-Carrascosa DP, Díez-Fernández A, Notario-Pacheco B. Risk of extrapyramidal side effects comparing continuous vs. bolus intravenous metoclopramide administration: a systematic review and meta-analysis of randomised controlled trials. J Clin Nurs. 2015;24(23-24):3638-3646. doi:10.1111/jocn.12984
167. Mikačić I, Bosnar D. Intravitreal Bevacizumab and Cardiovascular Risk in Patients with Age-Related Macular Degeneration: Systematic Review and Meta-Analysis of Randomized Controlled Trials and Observational Studies. Drug Saf. 2016;39(6):517-541. doi:10.1007/s40264-016-0408-y
168. Li-Kim-Moy J, Yin JK, Rashid H, et al. Systematic review of fever, febrile convulsions and serious adverse events following administration of inactivated trivalent influenza vaccines in children [published correction appears in Euro Surveill. 2015;20(25). pii: 21164]. Euro Surveill. 2015;20(24):21159. Published 2015 Jun 18. doi:10.2807/1560-7917.es2015.20.24.21159
169. Menne J, Dumann E, Haller H, Schmidt BMW. Acute kidney injury and adverse renal events in patients receiving SGLT2-inhibitors: A systematic review and meta-analysis. PLoS Med. 2019;16(12):e1002983. Published 2019 Dec 9. doi:10.1371/journal.pmed.1002983
170. Janjua S, Schmidt S, Ferrer M, Cates CJ. Inhaled steroids with and without regular formoterol for asthma: serious adverse events. Cochrane Database Syst Rev. 2019;9(9):CD006924. Published 2019 Sep 25. doi:10.1002/14651858.CD006924.pub4
171. Singh JA, Cameron C, Noorbaloochi S, et al. Risk of serious infection in biological treatment of patients with rheumatoid arthritis: a systematic review and meta-analysis. Lancet. 2015;386(9990):258-265. doi:10.1016/S0140-6736(14)61704-9
172. Singh JA, Hossain A, Kotb A, Wells G. Risk of serious infections with immunosuppressive drugs and glucocorticoids for lupus nephritis: a systematic review and network meta-analysis. BMC Med. 2016;14(1):137. Published 2016 Sep 13. doi:10.1186/s12916-016-0673-8
173. Bilal J, Berlinberg A, Riaz IB, et al. Risk of Infections and Cancer in Patients With Rheumatologic Diseases Receiving Interleukin Inhibitors: A Systematic Review and Meta-analysis. JAMA Netw Open. 2019;2(10):e1913102. Published 2019 Oct 2. doi:10.1001/jamanetworkopen.2019.13102
174. Wessler JD, Giugliano RP. Risk of thrombocytopenia with glycoprotein IIb/IIIa inhibitors across drugs and patient populations: a meta-analysis of 29 large placebo-controlled randomized trials [published correction appears in Eur Heart J Cardiovasc Pharmacother. 2016 Oct;2(4):217]. Eur Heart J Cardiovasc Pharmacother. 2015;1(2):97-106. doi:10.1093/ehjcvp/pvu008
175. Donnan JR, Grandy CA, Chibrikov E, et al. Comparative safety of the sodium glucose co-transporter 2 (SGLT2) inhibitors: a systematic review and meta-analysis. BMJ Open. 2019;9(1):e022577. Published 2019 Feb 1. doi:10.1136/bmjopen-2018-022577
176. de Filette J, Andreescu CE, Cools F, Bravenboer B, Velkeniers B. A Systematic Review and Meta-Analysis of Endocrine-Related Adverse Events Associated with Immune Checkpoint Inhibitors. Horm Metab Res. 2019;51(3):145-156. doi:10.1055/a-0843-3366
177. Chen J, Lu Y, Zheng Y. Incidence and risk of hypertension with bevacizumab in non-small-cell lung cancer patients: a meta-analysis of randomized controlled trials. Drug Des Devel Ther. 2015;9:4751-4760. Published 2015 Aug 18. doi:10.2147/DDDT.S87258
178. Zhou JX, Feng LJ, Zhang X. Risk of severe hematologic toxicities in cancer patients treated with PARP inhibitors: a meta-analysis of randomized controlled trials. Drug Des Devel Ther. 2017;11:3009-3017. Published 2017 Oct 13. doi:10.2147/DDDT.S147726
179. Sui JD, Wang Y, Wan Y, Wu YZ. Risk of hematologic toxicities with programmed cell death-1 inhibitors in cancer patients: a meta-analysis of current studies. Drug Des Devel Ther. 2018;12:1645-1657. Published 2018 Jun 8. doi:10.2147/DDDT.S167077
180. Zhu J, Wu J, Li G, et al. Meta-analysis of randomized controlled trials for the incidence and risk of fatal adverse events in cancer patients treated with ipilimumab. Expert Opin Drug Saf. 2017;16(4):423-428. doi:10.1080/14740338.2017.1297420
181. Zhu J, Liao R, Su C, et al. Toxicity profile characteristics of novel androgen-deprivation therapy agents in patients with prostate cancer: a meta-analysis. Expert Rev Anticancer Ther. 2018;18(2):193-198. doi:10.1080/14737140.2018.1419871
182. Zhu J, Zhao W, Liang D, et al. Risk of fatigue in cancer patients receiving anti-EGFR monoclonal antibodies: results from a systematic review and meta-analysis of randomized controlled trial. Int J Clin Oncol. 2018;23(2):389-399. doi:10.1007/s10147-017-1218-7
183. Wang J, Wang Z, Zhao Y. Incidence and risk of hypertension with ramucirumab in cancer patients: a meta-analysis of published studies. Clin Drug Investig. 2015;35(4):221-228. doi:10.1007/s40261-015-0272-z
184. Dong J, Meng X, Li S, et al. Risk of Adverse Vascular Events in Patients with Malignant Glioma Treated with Bevacizumab Plus Irinotecan: A Systematic Review and Meta-Analysis. World Neurosurg. 2019;130:e236-e243. doi:10.1016/j.wneu.2019.06.043
185. Ding J, Han J, Jing Z, Jiang Y. Is it dangerous to treat acute ischemic stroke by thrombolytic therapy in patients with comorbid intracranial aneurysms?. Am J Emerg Med. 2016;34(3):636-642. doi:10.1016/j.ajem.2015.12.025
186. Hua J, He Z, Qian D, Meng H, Zhou B, Song Z. Duct-to-Mucosa Versus Invagination Pancreaticojejunostomy Following Pancreaticoduodenectomy: a Systematic Review and Meta-Analysis. J Gastrointest Surg. 2015;19(10):1900-1909. doi:10.1007/s11605-015-2913-1
187. Li J, Gu J. Cardiovascular Toxicities with Vascular Endothelial Growth Factor Receptor Tyrosine Kinase Inhibitors in Cancer Patients: A Meta-Analysis of 77 Randomized Controlled Trials. Clin Drug Investig. 2018;38(12):1109-1123. doi:10.1007/s40261-018-0709-2
188. Li J, Yan H. Skin toxicity with anti-EGFR monoclonal antibody in cancer patients: a meta-analysis of 65 randomized controlled trials. Cancer Chemother Pharmacol. 2018;82(4):571-583. doi:10.1007/s00280-018-3644-2
189. Li J, Gu J. Rash and Pruritus With PD-1 Inhibitors in Cancer Patients: A Meta-Analysis of Randomized Controlled Trials. J Clin Pharmacol. 2019;59(1):45-54. doi:10.1002/jcph.1291
190. Zhao J, Xia Y, Kaminski J, et al. Treatment-Related Death during Concurrent Chemoradiotherapy for Locally Advanced Non-Small Cell Lung Cancer: A Meta-Analysis of Randomized Studies. PLoS One. 2016;11(6):e0157455. Published 2016 Jun 14. doi:10.1371/journal.pone.0157455
191. Schneider-Thoma J, Efthimiou O, Huhn M, et al. Second-generation antipsychotic drugs and short-term mortality: a systematic review and meta-analysis of placebo-controlled randomised controlled trials. Lancet Psychiatry. 2018;5(8):653-663. doi:10.1016/S2215-0366(18)30177-9
192. Schneider-Thoma J, Efthimiou O, Bighelli I, et al. Second-generation antipsychotic drugs and short-term somatic serious adverse events: a systematic review and meta-analysis. Lancet Psychiatry. 2019;6(9):753-765. doi:10.1016/S2215-0366(19)30223-8
193. Man J, Ritchie G, Links M, Lord S, Lee CK. Treatment-related toxicities of immune checkpoint inhibitors in advanced cancers: A meta-analysis. Asia Pac J Clin Oncol. 2018;14(3):141-152. doi:10.1111/ajco.12838
194. Stassijns J, Bollaerts K, Baay M, Verstraeten T. A systematic review and meta-analysis on the safety of newly adjuvanted vaccines among children. Vaccine. 2016;34(6):714-722. doi:10.1016/j.vaccine.2015.12.024
195. de Resende JA Júnior, Cavalini LT, Crispi CP, de Freitas Fonseca M. Risk of urinary retention after nerve-sparing surgery for deep infiltrating endometriosis: A systematic review and meta-analysis. Neurourol Urodyn. 2017;36(1):57-61. doi:10.1002/nau.22915
196. Berstock JR, Blom AW, Beswick AD. A systematic review and meta-analysis of complications following the posterior and lateral surgical approaches to total hip arthroplasty. Ann R Coll Surg Engl. 2015;97(1):11-16. doi:10.1308/003588414X13946184904008
197. Dai J, Belum VR, Wu S, Sibaud V, Lacouture ME. Pigmentary changes in patients treated with targeted anticancer agents: A systematic review and meta-analysis. J Am Acad Dermatol. 2017;77(5):902-910.e2. doi:10.1016/j.jaad.2017.06.044
198. Vavken J, Mameghani A, Vavken P, Schaeren S. Complications and cancer rates in spine fusion with recombinant human bone morphogenetic protein-2 (rhBMP-2). Eur Spine J. 2016;25(12):3979-3989. doi:10.1007/s00586-015-3870-9
199. Jesson J, Dahourou DL, Renaud F, Penazzato M, Leroy V. Adverse events associated with abacavir use in HIV-infected children and adolescents: a systematic review and meta-analysis. Lancet HIV. 2016;3(2):e64-e75. doi:10.1016/S2352-3018(15)00225-8
200. Jiang J, Zou J, Ma H, et al. Network Meta-analysis of Randomized Trials on the Safety of Vascular Closure Devices for Femoral Arterial Puncture Site Haemostasis. Sci Rep. 2015;5:13761. Published 2015 Sep 8. doi:10.1038/srep13761
201. Johansen KGV, Tarp S, Astrup A, Lund H, Pagsberg AK, Christensen R. Harms associated with taking nalmefene for substance use and impulse control disorders: A systematic review and meta-analysis of randomised controlled trials. PLoS One. 2017;12(8):e0183821. Published 2017 Aug 29. doi:10.1371/journal.pone.0183821
202. Mattishent K, Thavarajah M, Sinha A, et al. Safety of 80% vs 30-35% fraction of inspired oxygen in patients undergoing surgery: a systematic review and meta-analysis. Br J Anaesth. 2019;122(3):311-324. doi:10.1016/j.bja.2018.11.026
203. Zarrabi K, Wu S. Risk of Liver Toxicity with Nivolumab Immunotherapy in Cancer Patients. Oncology. 2018;94(5):259-273. doi:10.1159/000486679
204. Dahal K, Sharma S, Yousuf A, et al. A comparison of standard versus low dose heparin on access-related complications after coronary angiography through radial access: A meta-analysis of randomized controlled trials. Cardiovasc Revasc Med. 2018;19(5 Pt B):575-579. doi:10.1016/j.carrev.2017.10.018
205. Lao KS, He Y, Wong IC, Besag FM, Chan EW. Tolerability and Safety Profile of Cariprazine in Treating Psychotic Disorders, Bipolar Disorder and Major Depressive Disorder: A Systematic Review with Meta-Analysis of Randomized Controlled Trials. CNS Drugs. 2016;30(11):1043-1054. doi:10.1007/s40263-016-0382-z
206. Thong KM, Chan TM. Infectious complications in lupus nephritis treatment: a systematic review and meta-analysis. Lupus. 2019;28(3):334-346. doi:10.1177/0961203319829817
207. Almpani K, Papageorgiou SN, Papadopoulos MA. Autotransplantation of teeth in humans: a systematic review and meta-analysis. Clin Oral Investig. 2015;19(6):1157-1179. doi:10.1007/s00784-015-1473-9
208. Katsanos K, Spiliopoulos S, Kitrou P, Krokidis M, Karnabatidis D. Risk of Death Following Application of Paclitaxel-Coated Balloons and Stents in the Femoropopliteal Artery of the Leg: A Systematic Review and Meta-Analysis of Randomized Controlled Trials. J Am Heart Assoc. 2018;7(24):e011245. doi:10.1161/JAHA.118.011245
209. Dinh K, Limmer AM, Paravastu SCV, et al. Mortality After Paclitaxel-Coated Device Use in Dialysis Access: A Systematic Review and Meta-Analysis. J Endovasc Ther. 2019;26(5):600-612. doi:10.1177/1526602819872154
210. Abdelaziz HK, Megaly M, Debski M, et al. Meta-Analysis Comparing Percutaneous to Surgical Access in Trans-Femoral Transcatheter Aortic Valve Implantation. Am J Cardiol. 2020;125(8):1239-1248. doi:10.1016/j.amjcard.2020.01.021
211. Shah K, Chaker Z, Busu T, et al. Meta-Analysis Comparing Renal Outcomes after Transcatheter versus Surgical Aortic Valve Replacement. J Interv Cardiol. 2019;2019:3537256. Published 2019 Apr 24. doi:10.1155/2019/3537256
212. Thomas KH, Martin RM, Knipe DW, Higgins JP, Gunnell D. Risk of neuropsychiatric adverse events associated with varenicline: systematic review and meta-analysis. BMJ. 2015;350:h1109. Published 2015 Mar 12. doi:10.1136/bmj.h1109
213. Khoja L, Day D, Wei-Wu Chen T, Siu LL, Hansen AR. Tumour- and class-specific patterns of immune-related adverse events of immune checkpoint inhibitors: a systematic review. Ann Oncol. 2017;28(10):2377-2385. doi:10.1093/annonc/mdx286
214. Luo L, Yuan X, Huang W, et al. Safety of coadministration of ezetimibe and statins in patients with hypercholesterolaemia: a meta-analysis. Intern Med J. 2015;45(5):546-557. doi:10.1111/imj.12706
215. la Chapelle CF, Swank HA, Wessels ME, Mol BW, Rubinstein SM, Jansen FW. Trocar types in laparoscopy. Cochrane Database Syst Rev. 2015;(12):CD009814. Published 2015 Dec 16. doi:10.1002/14651858.CD009814.pub2
216. Touma L, Filion KB, Atallah R, Eberg M, Eisenberg MJ. A meta-analysis of randomized controlled trials of the risk of bleeding with apixaban versus vitamin K antagonists. Am J Cardiol. 2015;115(4):533-541. doi:10.1016/j.amjcard.2014.11.039
217. Quagliato LA, Cosci F, Shader RI, et al. Selective serotonin reuptake inhibitors and benzodiazepines in panic disorder: A meta-analysis of common side effects in acute treatment. J Psychopharmacol. 2019;33(11):1340-1351. doi:10.1177/0269881119859372
218. Azoulay L, Suissa S. Sulfonylureas and the Risks of Cardiovascular Events and Death: A Methodological Meta-Regression Analysis of the Observational Studies. Diabetes Care. 2017;40(5):706-714. doi:10.2337/dc16-1943
219. Sterling LH, Windle SB, Filion KB, Touma L, Eisenberg MJ. Varenicline and Adverse Cardiovascular Events: A Systematic Review and Meta-Analysis of Randomized Controlled Trials. J Am Heart Assoc. 2016;5(2):e002849. Published 2016 Feb 22. doi:10.1161/JAHA.115.002849
220. 李志霞,武珊珊,杨智荣,詹思延,孙凤.胰高血糖素样肽1受体激动剂类降糖药致2型糖尿病患者鼻咽炎和上呼吸道感染的网状meta分析[J].北京大学学报(医学版),2016,48(03):454-459.
221. Peng L, Ye X, Zhou Y, Zhang J, Zhao Q. Meta-analysis of incidence and risk of peripheral neuropathy associated with intravenous bortezomib. Support Care Cancer. 2015;23(9):2813-2824. doi:10.1007/s00520-015-2648-2
222. Peng L, Zhou Y, Ye X, Zhao Q. Treatment-related fatigue with everolimus and temsirolimus in patients with cancer-a meta-analysis of clinical trials. Tumour Biol. 2015;36(2):643-654. doi:10.1007/s13277-014-2669-3
223. Peng L, Bu Z, Ye X, Zhou Y, Zhao Q. Incidence and risk of peripheral neuropathy with nab-paclitaxel in patients with cancer: a meta-analysis. Eur J Cancer Care (Engl). 2017;26(5):10.1111/ecc.12407. doi:10.1111/ecc.12407
224. Li L, Zhang Z, Yao Z, et al. The impact of laryngeal mask versus other airways on perioperative respiratory adverse events in children: A systematic review and meta-analysis of randomized controlled trials. Int J Surg. 2019;64:40-48. doi:10.1016/j.ijsu.2019.02.020
225. Hou LQ, Jiang GX, Chen YF, et al. The Comparative Safety of TNF Inhibitors in Ankylosing Spondylitis-a Meta-Analysis Update of 14 Randomized Controlled Trials. Clin Rev Allergy Immunol. 2018;54(2):234-243. doi:10.1007/s12016-017-8623-6
226. Li L, Li Y, Xu X, et al. Safety evaluation on low-molecular-weight hydroxyethyl starch for volume expansion therapy in pediatric patients: a meta-analysis of randomized controlled trials. Crit Care. 2015;19(1):79. Published 2015 Mar 10. doi:10.1186/s13054-015-0815-y
227. Kassem L, Shohdy KS, Lasheen S, Abdel-Rahman O, Bachelot T. Hematological adverse effects in breast cancer patients treated with cyclin-dependent kinase 4 and 6 inhibitors: a systematic review and meta-analysis. Breast Cancer. 2018;25(1):17-27. doi:10.1007/s12282-017-0818-4
228. Furuya-Kanamori L, Doi SA, Onitilo A, Akhtar S. Is there truly an increase in risk of cardiovascular and hematological adverse events with vascular endothelial growth factor receptor tyrosine kinase inhibitors?. Expert Opin Drug Saf. 2020;19(2):223-228. doi:10.1080/14740338.2020.1691167
229. Tandan M, Cormican M, Vellinga A. Adverse events of fluoroquinolones vs. other antimicrobials prescribed in primary care: A systematic review and meta-analysis of randomized controlled trials. Int J Antimicrob Agents. 2018;52(5):529-540. doi:10.1016/j.ijantimicag.2018.04.014
230. de La Forest Divonne M, Gottenberg JE, Salliot C. Safety of biologic DMARDs in RA patients in real life: A systematic literature review and meta-analyses of biologic registers. Joint Bone Spine. 2017;84(2):133-140. doi:10.1016/j.jbspin.2016.02.028
231. Bakheet MF, Pearce LA, Hart RG. Effect of addition of clopidogrel to aspirin on subdural hematoma: meta-analysis of randomized clinical trials. Int J Stroke. 2015;10(4):501-505. doi:10.1111/ijs.12419
232. Gillies M, Ranakusuma A, Hoffmann T, et al. Common harms from amoxicillin: a systematic review and meta-analysis of randomized placebo-controlled trials for any indication. CMAJ. 2015;187(1):E21-E31. doi:10.1503/cmaj.140848
233. Lalu MM, Fayad A, Ahmed O, et al. Ultrasound-Guided Subclavian Vein Catheterization: A Systematic Review and Meta-Analysis. Crit Care Med. 2015;43(7):1498-1507. doi:10.1097/CCM.0000000000000973
234. Baay M, Bollaerts K, Verstraeten T. A systematic review and meta-analysis on the safety of newly adjuvanted vaccines among older adults. Vaccine. 2018;36(29):4207-4214. doi:10.1016/j.vaccine.2018.06.004
235. Ceresoli M, Tamini N, Gianotti L, Braga M, Nespoli L. Are endoscopic loop ties safe even in complicated acute appendicitis? A systematic review and meta-analysis. Int J Surg. 2019;68:40-47. doi:10.1016/j.ijsu.2019.06.011
236. Miroddi M, Sterrantino C, Simonelli I, Ciminata G, Phillips RS, Calapai G. Risk of grade 3-4 diarrhea and mucositis in colorectal cancer patients receiving anti-EGFR monoclonal antibodies regimens: A meta-analysis of 18 randomized controlled clinical trials. Crit Rev Oncol Hematol. 2015;96(2):355-371. doi:10.1016/j.critrevonc.2015.06.004
237. Miroddi M, Sterrantino C, Simmonds M, et al. Systematic review and meta-analysis of the risk of severe and life-threatening thromboembolism in cancer patients receiving anti-EGFR monoclonal antibodies (cetuximab or panitumumab). Int J Cancer. 2016;139(10):2370-2380. doi:10.1002/ijc.30280
238. Pozzi M, Carnovale C, Peeters GGAM, et al. Adverse drug events related to mood and emotion in paediatric patients treated for ADHD: A meta-analysis. J Affect Disord. 2018;238:161-178. doi:10.1016/j.jad.2018.05.021
239. Dore MP, Bibbò S, Fresi G, Bassotti G, Pes GM. Side Effects Associated with Probiotic Use in Adult Patients with Inflammatory Bowel Disease: A Systematic Review and Meta-Analysis of Randomized Controlled Trials. Nutrients. 2019;11(12):2913. Published 2019 Dec 2. doi:10.3390/nu11122913
240. Johnstone MS. Vertical Rectus Abdominis Myocutaneous Versus Alternative Flaps for Perineal Repair After Abdominoperineal Excision of the Rectum in the Era of Laparoscopic Surgery. Ann Plast Surg. 2017;79(1):101-106. doi:10.1097/SAP.0000000000001137
241. Parker M, Raval P, Gjertsen JE. Nail or plate fixation for A3 trochanteric hip fractures: A systematic review of randomised controlled trials. Injury. 2018;49(7):1319-1323. doi:10.1016/j.injury.2018.05.017
242. Franchini M, Mengoli C, Marietta M, et al. Safety of intravenous tranexamic acid in patients undergoing majororthopaedic surgery: a meta-analysis of randomised controlled trials. Blood Transfus. 2018;16(1):36-43. doi:10.2450//2017.0219-17
243. Mathew PJ, Mathew JL. Early versus late removal of the laryngeal mask airway (LMA) for general anaesthesia. Cochrane Database Syst Rev. 2015;(8):CD007082. Published 2015 Aug 10. doi:10.1002/14651858.CD007082.pub2
244. Holmskov M, Storebø OJ, Moreira-Maia CR, et al. Gastrointestinal adverse events during methylphenidate treatment of children and adolescents with attention deficit hyperactivity disorder: A systematic review with meta-analysis and Trial Sequential Analysis of randomised clinical trials. PLoS One. 2017;12(6):e0178187. Published 2017 Jun 15. doi:10.1371/journal.pone.0178187
245. Monami M, Nreu B, Zannoni S, Lualdi C, Mannucci E. Effects of SGLT-2 inhibitors on diabetic ketoacidosis: A meta-analysis of randomised controlled trials. Diabetes Res Clin Pract. 2017;130:53-60. doi:10.1016/j.diabres.2017.04.017
246. Monami M, Nreu B, Scatena A, et al. Safety issues with glucagon-like peptide-1 receptor agonists (pancreatitis, pancreatic cancer and cholelithiasis): Data from randomized controlled trials. Diabetes Obes Metab. 2017;19(9):1233-1241. doi:10.1111/dom.12926
247. Santoni M, Guerra F, Conti A, et al. Incidence and risk of cardiotoxicity in cancer patients treated with targeted therapies. Cancer Treat Rev. 2017;59:123-131. doi:10.1016/j.ctrv.2017.07.006
248. Totzeck M, Mincu RI, Rassaf T. Cardiovascular Adverse Events in Patients With Cancer Treated With Bevacizumab: A Meta-Analysis of More Than 20 000 Patients. J Am Heart Assoc. 2017;6(8):e006278. Published 2017 Aug 10. doi:10.1161/JAHA.117.006278
249. Mazaud C, Fardet L. Relative risk of and determinants for adverse events of methotrexate prescribed at a low dose: a systematic review and meta-analysis of randomized placebo-controlled trials. Br J Dermatol. 2017;177(4):978-986. doi:10.1111/bjd.15377
250. Cao M, Li F, Wang Y, Zhang J. Treatment-related serious adverse events and fatal adverse events with regorafenib in cancer patients: a meta-analysis of phase 3 randomized controlled trials. Invest New Drugs. 2017;35(6):834-838. doi:10.1007/s10637-017-0512-6
251. Brener MI, Bush A, Miller JM, Hasan RK. Influence of radial versus femoral access site on coronary angiography and intervention outcomes: A systematic review and meta-analysis. Catheter Cardiovasc Interv. 2017;90(7):1093-1104. doi:10.1002/ccd.27043
252. Inokuchi M, Otsuki S, Fujimori Y, Sato Y, Nakagawa M, Kojima K. Systematic review of anastomotic complications of esophagojejunostomy after laparoscopic total gastrectomy. World J Gastroenterol. 2015;21(32):9656-9665. doi:10.3748/wjg.v21.i32.9656
253. Inokuchi M, Sugita H, Otsuki S, Sato Y, Nakagawa M, Kojima K. Laparoscopic distal gastrectomy reduced surgical site infection as compared with open distal gastrectomy for gastric cancer in a meta-analysis of both randomized controlled and case-controlled studies. Int J Surg. 2015;15:61-67. doi:10.1016/j.ijsu.2015.01.030
254. Xu M, Nie Y, Yang Y, Lu YT, Su Q. Risk of Neurological Toxicities Following the Use of Different Immune Checkpoint Inhibitor Regimens in Solid Tumors: A Systematic Review and Meta-analysis. Neurologist. 2019;24(3):75-83. doi:10.1097/NRL.0000000000000230
255. Yang M, Zhang Y, Chen H, Lin J, Zeng J, Xu Z. Inhaled corticosteroids and risk of upper respiratory tract infection in patients with asthma: a meta-analysis. Infection. 2019;47(3):377-385. doi:10.1007/s15010-018-1229-y
256. Buller M, Schulz S, Kasdan M, Wilhelmi BJ. The Incidence of Complex Regional Pain Syndrome in Simultaneous Surgical Treatment of Carpal Tunnel Syndrome and Dupuytren Contracture. Hand (N Y). 2018;13(4):391-394. doi:10.1177/1558944717718345
257. Rahouma M, Karim NA, Baudo M, et al. Cardiotoxicity with immune system targeting drugs: a meta-analysis of anti-PD/PD-L1 immunotherapy randomized clinical trials. Immunotherapy. 2019;11(8):725-735. doi:10.2217/imt-2018-0118
258. Jalili M, Bahreini M, Doosti-Irani A, Masoomi R, Arbab M, Mirfazaelian H. Ketamine-propofol combination (ketofol) vs propofol for procedural sedation and analgesia: systematic review and meta-analysis. Am J Emerg Med. 2016;34(3):558-569. doi:10.1016/j.ajem.2015.12.074
259. Ruzieh M, Moroi MK, Aboujamous NM, et al. Meta-Analysis Comparing the Relative Risk of Adverse Events for Amiodarone Versus Placebo. Am J Cardiol. 2019;124(12):1889-1893. doi:10.1016/j.amjcard.2019.09.008
260. Bolton M, Hodkinson A, Boda S, et al. Serious adverse events reported in placebo randomised controlled trials of oral naltrexone: a systematic review and meta-analysis. BMC Med. 2019;17(1):10. Published 2019 Jan 15. doi:10.1186/s12916-018-1242-0
261. Henriksen NA, Deerenberg EB, Venclauskas L, et al. Triclosan-coated sutures and surgical site infection in abdominal surgery: the TRISTAN review, meta-analysis and trial sequential analysis. Hernia. 2017;21(6):833-841. doi:10.1007/s10029-017-1681-0
262. Ford N, Shubber Z, Pozniak A, et al. Comparative Safety and Neuropsychiatric Adverse Events Associated With Efavirenz Use in First-Line Antiretroviral Therapy: A Systematic Review and Meta-Analysis of Randomized Trials. J Acquir Immune Defic Syndr. 2015;69(4):422-429. doi:10.1097/QAI.0000000000000606
263. Taglieri N, Bacchi Reggiani ML, Ghetti G, et al. Risk of Stroke in Patients with Stable Coronary Artery Disease Undergoing Percutaneous Coronary Intervention versus Optimal Medical Therapy: Systematic Review and Meta-Analysis of Randomized Controlled Trials. PLoS One. 2016;11(7):e0158769. Published 2016 Jul 8. doi:10.1371/journal.pone.0158769
264. Fuggle N, Curtis E, Shaw S, et al. Safety of Opioids in Osteoarthritis: Outcomes of a Systematic Review and Meta-Analysis. Drugs Aging. 2019;36(Suppl 1):129-143. doi:10.1007/s40266-019-00666-9
265. Delanoy N, Pécuchet N, Fabre E, et al. Bleomycin-Induced Pneumonitis in the Treatment of Ovarian Sex Cord-Stromal Tumors: A Systematic Review and Meta-analysis. Int J Gynecol Cancer. 2015;25(9):1593-1598. doi:10.1097/IGC.0000000000000530
266. Riva N, Dentali F, Permunian ET, Ageno W. Major Bleeding and Case Fatality Rate with the Direct Oral Anticoagulants in Orthopedic Surgery: A Systematic Review and Meta-Analysis. Semin Thromb Hemost. 2016;42(1):42-54. doi:10.1055/s-0035-1568875
267. Pecorelli N, Greco M, Amodeo S, Braga M. Small bowel obstruction and incisional hernia after laparoscopic and open colorectal surgery: a meta-analysis of comparative trials. Surg Endosc. 2017;31(1):85-99. doi:10.1007/s00464-016-4995-6
268. Xia N, Wang H, Nie X. Inhaled Long-Acting β2-Agonists Do Not Increase Fatal Cardiovascular Adverse Events in COPD: A Meta-Analysis. PLoS One. 2015;10(9):e0137904. Published 2015 Sep 17. doi:10.1371/journal.pone.0137904
269. Switzer NJ, Dykstra MA, Gill RS, et al. Endoscopic versus open component separation: systematic review and meta-analysis. Surg Endosc. 2015;29(4):787-795. doi:10.1007/s00464-014-3741-1
270. Yamaguchi N, Fujii T, Aoi S, Kozuch PS, Hortobagyi GN, Blum RH. Comparison of cardiac events associated with liposomal doxorubicin, epirubicin and doxorubicin in breast cancer: a Bayesian network meta-analysis. Eur J Cancer. 2015;51(16):2314-2320. doi:10.1016/j.ejca.2015.07.031
271. Craveiro NS, Silva Lopes B, Tomás L, et al. L-TRUST: Long-term risk of cancer in patients under statins therapy. A systematic review and meta-analysis. Pharmacoepidemiol Drug Saf. 2019;28(11):1431-1439. doi:10.1002/pds.4895
272. Egunsola O, Choonara I, Sammons HM. Safety of lamotrigine in paediatrics: a systematic review. BMJ Open. 2015;5(6):e007711. Published 2015 Jun 12. doi:10.1136/bmjopen-2015-007711
273. Abdel-Rahman O, Oweira H, Petrausch U, et al. Immune-related ocular toxicities in solid tumor patients treated with immune checkpoint inhibitors: a systematic review. Expert Rev Anticancer Ther. 2017;17(4):387-394. doi:10.1080/14737140.2017.1296765
274. Abdel-Rahman O, ElHalawani H. Risk of hematological toxicities in patients with solid tumors treated with ramucirumab: a meta-analysis. Future Oncol. 2015;11(21):2949-2961. doi:10.2217/fon.15.178
275. Abdel-Rahman O, ElHalawani H, Fouad M. Risk of gastrointestinal complications in cancer patients treated with immune checkpoint inhibitors: a meta-analysis. Immunotherapy. 2015;7(11):1213-1227. doi:10.2217/imt.15.87
276. Abdel-Rahman O, Fouad M. Risk of oral and gastrointestinal mucosal injury in patients with solid tumors treated with everolimus, temsirolimus or ridaforolimus: a comparative systematic review and meta-analysis. Expert Rev Anticancer Ther. 2015;15(7):847-858. doi:10.1586/14737140.2015.1047350
277. Abdel-Rahman O, Fouad M. Risk of mucocutaneous toxicities in patients with solid tumors treated with lapatinib: a systematic review and meta-analysis. Curr Med Res Opin. 2015;31(5):975-986. doi:10.1185/03007995.2015.1020367
278. Abdel-Rahman O, ElHalawani H. Risk of cardiovascular adverse events in patients with solid tumors treated with ramucirumab: A meta analysis and summary of other VEGF targeted agents. Crit Rev Oncol Hematol. 2016;102:89-100. doi:10.1016/j.critrevonc.2016.04.003
279. Abdel-Rahman O, ElHalawani H, Essam-Eldin S. S-1-based regimens and the risk of leucopenic complications; a Meta-analysis with comparison to other fluoropyrimidines and non fluoropyrimidines. Expert Opin Drug Saf. 2016;15(4):437-448. doi:10.1517/14740338.2016.1146674
280. Abdel-Rahman O, ElHalawani H, Fouad M. Risk of endocrine complications in cancer patients treated with immune check point inhibitors: a meta-analysis. Future Oncol. 2016;12(3):413-425. doi:10.2217/fon.15.222
281. Abdel-Rahman O, Fouad M. A network meta-analysis of the risk of immune-related renal toxicity in cancer patients treated with immune checkpoint inhibitors. Immunotherapy. 2016;8(5):665-674. doi:10.2217/imt-2015-0020
282. Abdel-Rahman O, Helbling D, Schmidt J, et al. Treatment-associated Fatigue in Cancer Patients Treated with Immune Checkpoint Inhibitors; a Systematic Review and Meta-analysis. Clin Oncol (R Coll Radiol). 2016;28(10):e127-e138. doi:10.1016/j.clon.2016.06.008
283. Alfayez OM, Almutairi AR, Aldosari A, Al Yami MS. Update on Cardiovascular Safety of Incretin-Based Therapy in Adults With Type 2 Diabetes Mellitus: A Meta-Analysis of Cardiovascular Outcome Trials. Can J Diabetes. 2019;43(7):538-545.e2. doi:10.1016/j.jcjd.2019.04.003
284. Alfayez OM, Al Yami MS, Alshibani M, et al. Network meta-analysis of nine large cardiovascular outcome trials of new antidiabetic drugs. Prim Care Diabetes. 2019;13(3):204-211. doi:10.1016/j.pcd.2019.01.003
285. Liu P, Lin H, Chen Y, Wu YS, Tang M, Liu C. Comparison of Metal and Plastic Stents for Preoperative Biliary Drainage in Resectable and Borderline Resectable Periampullary Cancer: A Meta-Analysis and System Review. J Laparoendosc Adv Surg Tech A. 2018;28(9):1074-1082. doi:10.1089/lap.2018.0029
286. Zis P, Hadjivassiliou M, Sarrigiannis PG, Jenkins TM, Mitsikostas DD. Nocebo in chronic inflammatory demyelinating polyneuropathy; a systematic review and meta-analysis of placebo-controlled clinical trials. J Neurol Sci. 2018;388:79-83. doi:10.1016/j.jns.2018.03.009
287. Salas PAO, Parra CO, Florez CEP, Goez LM, Velez-van-Meerbeke A, Rodriguez JH. Safety liver profile of teriflunomide versus interferon β in multiple sclerosis: Systematic review and indirect comparison meta-analysis. Mult Scler Relat Disord. 2018;26:192-200. doi:10.1016/j.msard.2018.09.014
288. Rogliani P, Matera MG, Ora J, Cazzola M, Calzetta L. The impact of dual bronchodilation on cardiovascular serious adverse events and mortality in COPD: a quantitative synthesis [published correction appears in Int J Chron Obstruct Pulmon Dis. 2018 Oct 31;13:3597]. Int J Chron Obstruct Pulmon Dis. 2017;12:3469-3485. Published 2017 Dec 5. doi:10.2147/COPD.S146338
289. Sardar P, Udell JA, Chatterjee S, Bansilal S, Mukherjee D, Farkouh ME. Effect of Intensive Versus Standard Blood Glucose Control in Patients With Type 2 Diabetes Mellitus in Different Regions of the World: Systematic Review and Meta-analysis of Randomized Controlled Trials. J Am Heart Assoc. 2015;4(5):e001577. Published 2015 May 5. doi:10.1161/JAHA.114.001577
290. Wusiman P, Tayie A, Ling W, Moming A. Management of Mandibular Fractures Using Locking and Nonlocking Miniplates [published correction appears in J Craniofac Surg. 2019 Jul;30(5):1593]. J Craniofac Surg. 2019;30(2):448-452. doi:10.1097/SCS.0000000000005128
291. Nicolas P, Maia MF, Bassat Q, et al. Safety of oral ivermectin during pregnancy: a systematic review and meta-analysis. Lancet Glob Health. 2020;8(1):e92-e100. doi:10.1016/S2214-109X(19)30453-X
292. Scheiermann P, Herzog F, Siebenhofer A, Strametz R, Weberschock T. Intravenous versus inhalational anesthesia for pediatric inpatient surgery - A systematic review and meta-analysis. J Clin Anesth. 2018;49:19-25. doi:10.1016/j.jclinane.2018.05.014
293. Massey PR, Okman JS, Wilkerson J, Cowen EW. Tyrosine kinase inhibitors directed against the vascular endothelial growth factor receptor (VEGFR) have distinct cutaneous toxicity profiles: a meta-analysis and review of the literature. Support Care Cancer. 2015;23(6):1827-1835. doi:10.1007/s00520-014-2520-9
294. Moćko P, Kawalec P, Pilc A. Safety Profile of Biologic Drugs in the Therapy of Ulcerative Colitis: A Systematic Review and Network Meta-Analysis. Pharmacotherapy. 2016;36(8):870-879. doi:10.1002/phar.1785
295. Moćko P, Kawalec P, Pilc A. Safety profile of biologic drugs in the therapy of Crohn disease: A systematic review and network meta-analysis. Pharmacol Rep. 2016;68(6):1237-1243. doi:10.1016/j.pharep.2016.07.013
296. Moćko P, Kawalec P, Pilc A. Safety Profile of Biologic Drugs in the Treatment of Inflammatory Bowel Diseases: A Systematic Review and Network Meta-analysis of Randomized Controlled Trials. Clin Drug Investig. 2017;37(1):25-37. doi:10.1007/s40261-016-0459-y
297. Coelho PL, da Silva Calestini GL, Alvo FS, de Moura Freitas JM, Castro PM, Konstantyner T. Segurança da vacina papillomavirus humano 6, 11, 16 e 18 (recombinante): revisão sistemática e metanálise. Rev Paul Pediatr. 2015;33(4):474-482. doi:10.1016/j.rpped.2015.02.006
298. Ding PN, Lord SJ, Gebski V, et al. Risk of Treatment-Related Toxicities from EGFR Tyrosine Kinase Inhibitors: A Meta-analysis of Clinical Trials of Gefitinib, Erlotinib, and Afatinib in Advanced EGFR-Mutated Non-Small Cell Lung Cancer. J Thorac Oncol. 2017;12(4):633-643. doi:10.1016/j.jtho.2016.11.2236
299. Dorjee P, Long ZW. A mixed treatment comparison of toxicity of gemcitabine combined with different targeted drugs in the treatment of advanced or metastatic pancreatic cancer. Cancer Biol Ther. 2018;19(6):497-506. doi:10.1080/15384047.2018.1433503
300. Elwood PC, Morgan G, Galante J, et al. Systematic Review and Meta-Analysis of Randomised Trials to Ascertain Fatal Gastrointestinal Bleeding Events Attributable to Preventive Low-Dose Aspirin: No Evidence of Increased Risk. PLoS One. 2016;11(11):e0166166. Published 2016 Nov 15. doi:10.1371/journal.pone.0166166
301. Polderman JA, Farhang-Razi V, Van Dieren S, et al. Adverse side effects of dexamethasone in surgical patients. Cochrane Database Syst Rev. 2018;8(8):CD011940. Published 2018 Aug 28. doi:10.1002/14651858.CD011940.pub2
302. Ghatalia P, Je Y, Nguyen PL, Trinh QD, Choueiri TK, Sonpavde G. Fatigue with vascular endothelial growth factor receptor tyrosine kinase inhibitors and mammalian target of rapamycin inhibitors in patients with renal cell carcinoma (RCC) and other malignancies: A meta-analysis of randomized clinical trials. Crit Rev Oncol Hematol. 2015;95(2):251-263. doi:10.1016/j.critrevonc.2015.03.006
303. Ghayoumi P, Kandemir U, Morshed S. Evidence based update: open versus closed reduction. Injury. 2015;46(3):467-473. doi:10.1016/j.injury.2014.10.011
304. Bundhun PK, Janoo G, Chen MH. Bleeding events associated with fibrinolytic therapy and primary percutaneous coronary intervention in patients with STEMI: A systematic review and meta-analysis of randomized controlled trials [published correction appears in Medicine (Baltimore). 2016 Jul 18;95(28):e0916]. Medicine (Baltimore). 2016;95(23):e3877. doi:10.1097/MD.0000000000003877
305. Bundhun PK, Bhurtu A, Pursun M, Soogund MZS, Teeluck AR, Huang WQ. Long-term (2-5 years) adverse clinical outcomes associated with ZES versus SES, PES and EES: A Meta-Analysis. Sci Rep. 2017;7(1):6385. Published 2017 Jul 25. doi:10.1038/s41598-017-06705-y
306. Bundhun PK, Janoo G, Huang F. Adverse drug events observed in patients with type 2 diabetes mellitus treated with 100 mg versus 300 mg canagliflozin: a systematic review and meta-analysis of published randomized controlled trials. BMC Pharmacol Toxicol. 2017;18(1):19. Published 2017 Apr 16. doi:10.1186/s40360-017-0126-9
307. Zhu Q, Hu H, Weng DS, et al. Pooled safety analyses of ALK-TKI inhibitor in ALK-positive NSCLC. BMC Cancer. 2017;17(1):412. Published 2017 Jun 12. doi:10.1186/s12885-017-3405-3
308. Su Q, Zhang XC, Wang DY, et al. The risk of immune-related endocrine disorders associated with anti-PD-1 inhibitors therapy for solid tumors: A systematic review and meta-analysis. Int Immunopharmacol. 2018;59:328-338. doi:10.1016/j.intimp.2018.04.021
309. Hua Q, Zhu Y, Liu H. Severe and fatal adverse events risk associated with rituximab addition to B-cell non-Hodgkin's lymphoma (B-NHL) chemotherapy: a meta-analysis. J Chemother. 2015;27(6):365-370. doi:10.1179/1973947815Y.0000000025
310. Cai Q, Feng L, Yap KZ. Systematic review and meta-analysis of reported adverse events of long-term intranasal oxytocin treatment for autism spectrum disorder. Psychiatry Clin Neurosci. 2018;72(3):140-151. doi:10.1111/pcn.12627
311. Di Franco R, Borzillo V, Ravo V, et al. Rectal/urinary toxicity after hypofractionated vs. conventional radiotherapy in high risk prostate cancer: systematic review and meta analysis. Eur Rev Med Pharmacol Sci. 2017;21(16):3563-3575.
312. Bannuru RR, Osani M, Vaysbrot EE, McAlindon TE. Comparative safety profile of hyaluronic acid products for knee osteoarthritis: a systematic review and network meta-analysis. Osteoarthritis Cartilage. 2016;24(12):2022-2041. doi:10.1016/j.joca.2016.07.010
313. Mincu RI, Mahabadi AA, Michel L, et al. Cardiovascular Adverse Events Associated With BRAF and MEK Inhibitors: A Systematic Review and Meta-analysis. JAMA Netw Open. 2019;2(8):e198890. Published 2019 Aug 2. doi:10.1001/jamanetworkopen.2019.8890
314. Meister R, von Wolff A, Mohr H, et al. Comparative Safety of Pharmacologic Treatments for Persistent Depressive Disorder: A Systematic Review and Network Meta-Analysis. PLoS One. 2016;11(5):e0153380. Published 2016 May 17. doi:10.1371/journal.pone.0153380
315. Cui R, Chu L, Liu ZQ, et al. Hematologic toxicity assessment in solid tumor patients treated with cetuximab: a pooled analysis of 18 randomized controlled trials [published correction appears in Int J Cancer. 2016 Apr 15;138(8):E4]. Int J Cancer. 2015;136(4):936-944. doi:10.1002/ijc.29045
316. Moreira R.B., de Biasi M., Francini E., Nuzzo P.V., de Velasco G., Maluf F.C., Fay A.P., Bellmunt J., Choueiri T.K., Schutz F.A. Differential side effects profile in patients with mCRPC treated with abiraterone or enzalutamide: A meta-analysis of randomized controlled trials. Oncotarget. 2017;8:84572–84578. doi: 10.18632/oncotarget.20028.
317. Pillai RN, Behera M, Owonikoko TK, et al. Comparison of the toxicity profile of PD-1 versus PD-L1 inhibitors in non-small cell lung cancer: A systematic analysis of the literature. Cancer. 2018;124(2):271-277. doi:10.1002/cncr.31043
318. Siemieniuk RA, Foroutan F, Mirza R, et al. Antiretroviral therapy for pregnant women living with HIV or hepatitis B: a systematic review and meta-analysis. BMJ Open. 2017;7(9):e019022. Published 2017 Sep 11. doi:10.1136/bmjopen-2017-019022
319. Costa R, Carneiro BA, Agulnik M, et al. Toxicity profile of approved anti-PD-1 monoclonal antibodies in solid tumors: a systematic review and meta-analysis of randomized clinical trials. Oncotarget. 2017;8(5):8910-8920. doi:10.18632/oncotarget.13315
320. Conway R, Low C, Coughlan RJ, O'Donnell MJ, Carey JJ. Risk of liver injury among methotrexate users: A meta-analysis of randomised controlled trials. Semin Arthritis Rheum. 2015;45(2):156-162. doi:10.1016/j.semarthrit.2015.05.003
321. Conway R, Low C, Coughlan RJ, O'Donnell MJ, Carey JJ. Methotrexate use and risk of lung disease in psoriasis, psoriatic arthritis, and inflammatory bowel disease: systematic literature review and meta-analysis of randomised controlled trials. BMJ. 2015;350:h1269. Published 2015 Mar 13. doi:10.1136/bmj.h1269
322. Conway R, Low C, Coughlan RJ, O'Donnell MJ, Carey JJ. Leflunomide Use and Risk of Lung Disease in Rheumatoid Arthritis: A Systematic Literature Review and Metaanalysis of Randomized Controlled Trials. J Rheumatol. 2016;43(5):855-860. doi:10.3899/jrheum.150674
323. Desai RJ, Thaler KJ, Mahlknecht P, et al. Comparative Risk of Harm Associated With the Use of Targeted Immunomodulators: A Systematic Review. Arthritis Care Res (Hoboken). 2016;68(8):1078-1088. doi:10.1002/acr.22815
324. Avery RL, Gordon GM. Systemic Safety of Prolonged Monthly Anti-Vascular Endothelial Growth Factor Therapy for Diabetic Macular Edema: A Systematic Review and Meta-analysis. JAMA Ophthalmol. 2016;134(1):21-29. doi:10.1001/jamaophthalmol.2015.4070
325. Puckrin R, Saltiel MP, Reynier P, Azoulay L, Yu OHY, Filion KB. SGLT-2 inhibitors and the risk of infections: a systematic review and meta-analysis of randomized controlled trials. Acta Diabetol. 2018;55(5):503-514. doi:10.1007/s00592-018-1116-0
326. Chow R, Bruera E, Arends J, et al. Enteral and parenteral nutrition in cancer patients, a comparison of complication rates: an updated systematic review and (cumulative) meta-analysis [published correction appears in Support Care Cancer. 2019 Dec 31;:]. Support Care Cancer. 2020;28(3):979-1010. doi:10.1007/s00520-019-05145-w
327. Lucchetta RC, Leonart LP, Becker J, Pontarolo R, Fernandez-Llimós F, Wiens A. Safety outcomes of disease-modifying therapies for relapsing-remitting multiple sclerosis: A network meta-analysis. Mult Scler Relat Disord. 2019;35:7-15. doi:10.1016/j.msard.2019.06.036
328. Klotz R, Probst P, Deininger M, et al. Percutaneous versus surgical strategy for tracheostomy: a systematic review and meta-analysis of perioperative and postoperative complications. Langenbecks Arch Surg. 2018;403(2):137-149. doi:10.1007/s00423-017-1648-8
329. Di Franco R, Borzillo V, Ravo V, et al. Rectal/urinary toxicity after hypofractionated vs conventional radiotherapy in low/intermediate risk localized prostate cancer: systematic review and meta analysis. Oncotarget. 2017;8(10):17383-17395. doi:10.18632/oncotarget.14798
330. Gaffar R, Habib B, Filion KB, Reynier P, Eisenberg MJ. Optimal Timing of Complete Revascularization in Acute Coronary Syndrome: A Systematic Review and Meta-Analysis. J Am Heart Assoc. 2017;6(4):e005381. Published 2017 Apr 10. doi:10.1161/JAHA.116.005381
331. Tian R, Yan H, Zhang F, et al. Incidence and relative risk of hemorrhagic events associated with ramucirumab in cancer patients: a systematic review and meta-analysis. Oncotarget. 2016;7(40):66182-66191. doi:10.18632/oncotarget.11097
332. Xu R, Lian Y, Li WX. Airway Complications during and after General Anesthesia: A Comparison, Systematic Review and Meta-Analysis of Using Flexible Laryngeal Mask Airways and Endotracheal Tubes. PLoS One. 2016;11(7):e0158137. Published 2016 Jul 14. doi:10.1371/journal.pone.0158137
333. Mozaffari S, Abdolghaffari AH, Nikfar S, Abdollahi M. Pregnancy outcomes in women with inflammatory bowel disease following exposure to thiopurines and antitumor necrosis factor drugs: a systematic review with meta-analysis. Hum Exp Toxicol. 2015;34(5):445-459. doi:10.1177/0960327114550882
334. Lewis SR, Butler AR, Parker J, Cook TM, Schofield-Robinson OJ, Smith AF. Videolaryngoscopy versus direct laryngoscopy for adult patients requiring tracheal intubation: a Cochrane Systematic Review. Br J Anaesth. 2017;119(3):369-383. doi:10.1093/bja/aex228
335. Sun S, Cui Z, Zhou M, et al. Proton pump inhibitor monotherapy and the risk of cardiovascular events in patients with gastro-esophageal reflux disease: a meta-analysis. Neurogastroenterol Motil. 2017;29(2):10.1111/nmo.12926. doi:10.1111/nmo.12926
336. Ghumman SS, Weinerman J, Khan A, et al. Contrast induced-acute kidney injury following peripheral angiography with carbon dioxide versus iodinated contrast media: A meta-analysis and systematic review of current literature. Catheter Cardiovasc Interv. 2017;90(3):437-448. doi:10.1002/ccd.27051
337. Martel S, Bruzzone M, Ceppi M, et al. Risk of adverse events with the addition of targeted agents to endocrine therapy in patients with hormone receptor-positive metastatic breast cancer: A systematic review and meta-analysis. Cancer Treat Rev. 2018;62:123-132. doi:10.1016/j.ctrv.2017.09.009
338. Manohar S, Kompotiatis P, Thongprayoon C, Cheungpasitporn W, Herrmann J, Herrmann SM. Programmed cell death protein 1 inhibitor treatment is associated with acute kidney injury and hypocalcemia: meta-analysis. Nephrol Dial Transplant. 2019;34(1):108-117. doi:10.1093/ndt/gfy105
339. Donegan S, Dixon P, Hemming K, Tudur-Smith C, Marson A. A systematic review of placebo-controlled trials of topiramate: How useful is a multiple-indications review for evaluating the adverse events of an antiepileptic drug?. Epilepsia. 2015;56(12):1910-1920. doi:10.1111/epi.13209
340. Vouri SM, Kebodeaux CD, Stranges PM, Teshome BF. Adverse events and treatment discontinuations of antimuscarinics for the treatment of overactive bladder in older adults: A systematic review and meta-analysis. Arch Gerontol Geriatr. 2017;69:77-96. doi:10.1016/j.archger.2016.11.006
341. Wang S, He Q, Shuai Z. Risk of serious infections in biological treatment of patients with ankylosing spondylitis and non-radiographic axial spondyloarthritis: a meta-analysis. Clin Rheumatol. 2018;37(2):439-450. doi:10.1007/s10067-017-3966-1
342. Yun S, Vincelette ND, Acharya U, Abraham I. Risk of Atrial Fibrillation and Bleeding Diathesis Associated With Ibrutinib Treatment: A Systematic Review and Pooled Analysis of Four Randomized Controlled Trials. Clin Lymphoma Myeloma Leuk. 2017;17(1):31-37.e13. doi:10.1016/j.clml.2016.09.010
343. Lasheen S, Shohdy KS, Kassem L, Abdel-Rahman O. Fatigue, alopecia and stomatitis among patients with breast cancer receiving cyclin-dependent kinase 4 and 6 inhibitors: a systematic review and meta-analysis. Expert Rev Anticancer Ther. 2017;17(9):851-856. doi:10.1080/14737140.2017.1355242
344. Wu S, Chai S, Yang J, et al. Gastrointestinal Adverse Events of Dipeptidyl Peptidase 4 Inhibitors in Type 2 Diabetes: A Systematic Review and Network Meta-analysis. Clin Ther. 2017;39(9):1780-1789.e33. doi:10.1016/j.clinthera.2017.07.036
345. Wu S, Cipriani A, Yang Z, et al. The cardiovascular effect of incretin-based therapies among type 2 diabetes: a systematic review and network meta-analysis. Expert Opin Drug Saf. 2018;17(3):243-249. doi:10.1080/14740338.2018.1424826
346. Ding SX, Chen T, Wang T, Liu CY, Lu WL, Fu R. The Risk of Clonal Evolution of Granulocyte Colony-Stimulating Factor for Acquired Aplastic Anemia: A Systematic Review and Meta-Analysis. Acta Haematol. 2018;140(3):141-145. doi:10.1159/000491816
347. Zhang S, Liang F, Zhu J, Chen Q. Risk of Pneumonitis Associated with Programmed Cell Death 1 Inhibitors in Cancer Patients: A Meta-analysis. Mol Cancer Ther. 2017;16(8):1588-1595. doi:10.1158/1535-7163.MCT-17-0155
348. Zhang S, Liang F, Li W, Wang Q. Risk of treatment-related mortality in cancer patients treated with ipilimumab: A systematic review and meta-analysis. Eur J Cancer. 2017;83:71-79. doi:10.1016/j.ejca.2017.06.021
349. Guo S, Chen L, Cheng S, Xu H. Comparative cardiovascular safety of selective serotonin reuptake inhibitors (SSRIs) among Chinese senile depression patients: A network meta-analysis of randomized controlled trials. Medicine (Baltimore). 2019;98(22):e15786. doi:10.1097/MD.0000000000015786
350. Ruan SY, Huang TM, Wu HY, Wu HD, Yu CJ, Lai MS. Inhaled nitric oxide therapy and risk of renal dysfunction: a systematic review and meta-analysis of randomized trials. Crit Care. 2015;19(1):137. Published 2015 Apr 3. doi:10.1186/s13054-015-0880-2
351. Rogers SC, Garcia CA, Wu S. Discontinuation of Everolimus Due to Related and Unrelated Adverse Events in Cancer Patients: A Meta-Analysis. Cancer Invest. 2017;35(8):552-561. doi:10.1080/07357907.2017.1344697
352. Lv S, Wang J, Xu Y. Safety of insulin analogs during pregnancy: a meta-analysis. Arch Gynecol Obstet. 2015;292(4):749-756. doi:10.1007/s00404-015-3692-3
353. Huang ST, Tian BS, Xiao O, Yang YJ, Zhou SY. Safety of antivascular endothelial growth factor administration in the ocular anterior segment in pterygium and neovascular glaucoma treatment: Systematic review and meta-analysis. Medicine (Baltimore). 2018;97(34):e11960. doi:10.1097/MD.0000000000011960
354. Baxi S, Yang A, Gennarelli RL, et al. Immune-related adverse events for anti-PD-1 and anti-PD-L1 drugs: systematic review and meta-analysis. BMJ. 2018;360:k793. Published 2018 Mar 14. doi:10.1136/bmj.k793
355. Hao S, Tian W, Gao B, et al. Does dual HER-2 blockade treatment increase the risk of severe toxicities of special interests in breast cancer patients: A meta-analysis of randomized controlled trials. Oncotarget. 2017;8(12):19923-19933. doi:10.18632/oncotarget.15252
356. Jin S, Zhou X. Influence of dexmedetomidine on cardiac complications in non-cardiac surgery: a meta-analysis of randomized trials. Int J Clin Pharm. 2017;39(4):629-640. doi:10.1007/s11096-017-0493-8
357. Minozzi S, Bonovas S, Lytras T, et al. Risk of infections using anti-TNF agents in rheumatoid arthritis, psoriatic arthritis, and ankylosing spondylitis: a systematic review and meta-analysis. Expert Opin Drug Saf. 2016;15(sup1):11-34. doi:10.1080/14740338.2016.1240783
358. Tarp S, Eric Furst D, Boers M, et al. Risk of serious adverse effects of biological and targeted drugs in patients with rheumatoid arthritis: a systematic review meta-analysis. Rheumatology (Oxford). 2017;56(3):417-425. doi:10.1093/rheumatology/kew442
359. Salim SA, Cheungpasitporn W, Elmaraezy A, et al. Infectious complications and mortality associated with the use of IV iron therapy: a systematic review and meta-analysis. Int Urol Nephrol. 2019;51(10):1855-1865. doi:10.1007/s11255-019-02273-4
360. Sonalkar S, Kapp N. Intrauterine device insertion in the postpartum period: a systematic review. Eur J Contracept Reprod Health Care. 2015;20(1):4-18. doi:10.3109/13625187.2014.971454
361. Tong S, Fan K, Jiang K, et al. Increased risk of severe infections in non-small-cell lung cancer patients treated with pemetrexed: a meta-analysis of randomized controlled trials. Curr Med Res Opin. 2017;33(1):31-37. doi:10.1080/03007995.2016.1232705
362. Grajek S, Michalak M, Gwizdała A, et al. Patients treated with bivalirudin are still at higher risk of stent thrombosis: a comprehensive meta-analysis of randomised clinical trials of bivalirudin and heparin for percutaneous coronary interventions. Kardiol Pol. 2018;76(4):740-749. doi:10.5603/KP.a2018.0024
363. Bonovas S, Fiorino G, Allocca M, et al. Biologic Therapies and Risk of Infection and Malignancy in Patients With Inflammatory Bowel Disease: A Systematic Review and Network Meta-analysis. Clin Gastroenterol Hepatol. 2016;14(10):1385-1397.e10. doi:10.1016/j.cgh.2016.04.039
364. Bonovas S, Minozzi S, Lytras T, et al. Risk of malignancies using anti-TNF agents in rheumatoid arthritis, psoriatic arthritis, and ankylosing spondylitis: a systematic review and meta-analysis. Expert Opin Drug Saf. 2016;15(sup1):35-54. doi:10.1080/14740338.2016.1238458
365. Bonovas S, Nikolopoulos GK, Lytras T, Fiorino G, Peyrin-Biroulet L, Danese S. Comparative safety of systemic and low-bioavailability steroids in inflammatory bowel disease: Systematic review and network meta-analysis. Br J Clin Pharmacol. 2018;84(2):239-251. doi:10.1111/bcp.13456
366. Kovacs SD, van Eijk AM, Sevene E, et al. The Safety of Artemisinin Derivatives for the Treatment of Malaria in the 2nd or 3rd Trimester of Pregnancy: A Systematic Review and Meta-Analysis. PLoS One. 2016;11(11):e0164963. Published 2016 Nov 8. doi:10.1371/journal.pone.0164963
367. Andersen SE, Christensen M. Hypoglycaemia when adding sulphonylurea to metformin: a systematic review and network meta-analysis. Br J Clin Pharmacol. 2016;82(5):1291-1302. doi:10.1111/bcp.13059
368. Lew S, Chamberlain RS. Risk of Metabolic Complications in Patients with Solid Tumors Treated with mTOR inhibitors: Meta-analysis. Anticancer Res. 2016;36(4):1711-1718.
369. Isomura T, Suzuki S, Origasa H, et al. Liver-related safety assessment of green tea extracts in humans: a systematic review of randomized controlled trials [published correction appears in Eur J Clin Nutr. 2016 Nov;70(11):1340]. Eur J Clin Nutr. 2016;70(11):1221-1229. doi:10.1038/ejcn.2016.78
370. Thakker D, Nair S, Pagada A, Jamdade V, Malik A. Statin use and the risk of developing diabetes: a network meta-analysis. Pharmacoepidemiol Drug Saf. 2016;25(10):1131-1149. doi:10.1002/pds.4020
371. Kötter T, da Costa BR, Fässler M, et al. Metamizole-associated adverse events: a systematic review and meta-analysis. PLoS One. 2015;10(4):e0122918. Published 2015 Apr 13. doi:10.1371/journal.pone.0122918
372. Wang T, Wang F, Zhou J, Tang H, Giovenale S. Adverse effects of incretin-based therapies on major cardiovascular and arrhythmia events: meta-analysis of randomized trials. Diabetes Metab Res Rev. 2016;32(8):843-857. doi:10.1002/dmrr.2804
373. Avni T, Bieber A, Grossman A, Green H, Leibovici L, Gafter-Gvili A. The safety of intravenous iron preparations: systematic review and meta-analysis. Mayo Clin Proc. 2015;90(1):12-23. doi:10.1016/j.mayocp.2014.10.007
374. Ando T, Briasoulis A, Holmes AA, Takagi H, Slovut DP. Percutaneous versus surgical cut-down access in transfemoral transcatheter aortic valve replacement: A meta-analysis. J Card Surg. 2016;31(12):710-717. doi:10.1111/jocs.12842
375. Ando T, Ashraf S, Villablanca PA, et al. Meta-Analysis Comparing the Incidence of Infective Endocarditis Following Transcatheter Aortic Valve Implantation Versus Surgical Aortic Valve Replacement. Am J Cardiol. 2019;123(5):827-832. doi:10.1016/j.amjcard.2018.11.031
376. Nishijima TF, Shachar SS, Nyrop KA, Muss HB. Safety and Tolerability of PD-1/PD-L1 Inhibitors Compared with Chemotherapy in Patients with Advanced Cancer: A Meta-Analysis. Oncologist. 2017;22(4):470-479. doi:10.1634/theoncologist.2016-0419
377. Funakoshi T, Suzuki M, Muss HB. Infection risk in breast cancer patients treated with trastuzumab: a systematic review and meta-analysis. Breast Cancer Res Treat. 2015;149(2):321-330. doi:10.1007/s10549-014-3184-3
378. Zhang T, Pope JE. Cardiovascular effects of urate-lowering therapies in patients with chronic gout: a systematic review and meta-analysis. Rheumatology (Oxford). 2017;56(7):1144-1153. doi:10.1093/rheumatology/kex065
379. Stub T, Musial F, Kristoffersen AA, Alræk T, Liu J. Adverse effects of homeopathy, what do we know? A systematic review and meta-analysis of randomized controlled trials. Complement Ther Med. 2016;26:146-163. doi:10.1016/j.ctim.2016.03.013
380. Ferreira VL, Assis Jarek NA, Tonin FS, Borba HH, Wiens A, Pontarolo R. Safety of interferon-free therapies for chronic hepatitis C: a network meta-analysis. J Clin Pharm Ther. 2016;41(5):478-485. doi:10.1111/jcpt.12426
381. Belum VR, Serna-Tamayo C, Wu S, Lacouture ME. Incidence and risk of hand-foot skin reaction with cabozantinib, a novel multikinase inhibitor: a meta-analysis. Clin Exp Dermatol. 2016;41(1):8-15. doi:10.1111/ced.12694
382. Belum VR, Benhuri B, Postow MA, et al. Characterisation and management of dermatologic adverse events to agents targeting the PD-1 receptor. Eur J Cancer. 2016;60:12-25. doi:10.1016/j.ejca.2016.02.010
383. Wadhwa V, Issa D, Garg S, Lopez R, Sanaka MR, Vargo JJ. Similar Risk of Cardiopulmonary Adverse Events Between Propofol and Traditional Anesthesia for Gastrointestinal Endoscopy: A Systematic Review and Meta-analysis. Clin Gastroenterol Hepatol. 2017;15(2):194-206. doi:10.1016/j.cgh.2016.07.013
384. Shahi V, Brinjikji W, Murad MH, Asirvatham SJ, Kallmes DF. Safety of Uninterrupted Warfarin Therapy in Patients Undergoing Cardiovascular Endovascular Procedures: A Systematic Review and Meta-Analysis. Radiology. 2016;278(2):383-394. doi:10.1148/radiol.2015142531
385. Pavlova V, Filipova E, Uzunova K, Kalinov K, Vekov T. Pioglitazone Therapy and Fractures: Systematic Review and Meta- Analysis. Endocr Metab Immune Disord Drug Targets. 2018;18(5):502-507. doi:10.2174/1871530318666180423121833
386. Favilla V, Russo GI, Privitera S, et al. Impact of combination therapy 5-alpha reductase inhibitors (5-ARI) plus alpha-blockers (AB) on erectile dysfunction and decrease of libido in patients with LUTS/BPH: a systematic review with meta-analysis. Aging Male. 2016;19(3):175-181. doi:10.1080/13685538.2016.1195361
387. Vinnakota DN, Kamatham R. Safety profile of phentolamine mesylate as reversal agent of pulpal and soft tissue dental anesthesia: a systematic review and meta-analysis. Quintessence Int. 2019;50(7):568-575. doi:10.3290/j.qi.a42574
388. Rungapiromnan W, Yiu ZZN, Warren RB, Griffiths CEM, Ashcroft DM. Impact of biologic therapies on risk of major adverse cardiovascular events in patients with psoriasis: systematic review and meta-analysis of randomized controlled trials. Br J Dermatol. 2017;176(4):890-901. doi:10.1111/bjd.14964
389. Ma W, Xu M, Liu Y, et al. Safety profile of combined therapy inhibiting EFGR and VEGF pathways in patients with advanced non-small-cell lung cancer: A meta-analysis of 15 phase II/III randomized trials. Int J Cancer. 2015;137(2):409-419. doi:10.1002/ijc.29377
390. Liu W, Ma X, Zhou W. Adverse events of benralizumab in moderate to severe eosinophilic asthma: A meta-analysis. Medicine (Baltimore). 2019;98(22):e15868. doi:10.1097/MD.0000000000015868
391. Gong W, Li A, Ai H, Shi H, Wang X, Nie S. Safety of early discharge after primary angioplasty in low-risk patients with ST-segment elevation myocardial infarction: A meta-analysis of randomised controlled trials. Eur J Prev Cardiol. 2018;25(8):807-815. doi:10.1177/2047487318763823
392. Wei W, Luo Z. Risk of gastrointestinal toxicities with PD-1 inhibitors in cancer patients: A meta-analysis of randomized clinical trials. Medicine (Baltimore). 2017;96(48):e8931. doi:10.1097/MD.0000000000008931
393. Zhang W, Lu M, Zhang C, et al. Therapeutic hypothermia increases the risk of cardiac arrhythmia for perinatal hypoxic ischaemic encephalopathy: A meta-analysis. PLoS One. 2017;12(3):e0173006. Published 2017 Mar 8. doi:10.1371/journal.pone.0173006
394. Qi WX, Fu S, Zhang Q, Guo XM. Bevacizumab increases the risk of infections in cancer patients: A systematic review and pooled analysis of 41 randomized controlled trials. Crit Rev Oncol Hematol. 2015;94(3):323-336. doi:10.1016/j.critrevonc.2015.02.007
395. Qi WX, Fu S, Zhang Q, Guo XM. Incidence and risk of hypertension associated with ramucirumab in cancer patients: A systematic review and meta-analysis. J Cancer Res Ther. 2016;12(2):775-781. doi:10.4103/0973-1482.148700
396. Xie W, Huang Y, Xiao S, Sun X, Fan Y, Zhang Z. Impact of Janus kinase inhibitors on risk of cardiovascular events in patients with rheumatoid arthritis: systematic review and meta-analysis of randomised controlled trials. Ann Rheum Dis. 2019;78(8):1048-1054. doi:10.1136/annrheumdis-2018-214846
397. Wang W, Lie P, Guo M, He J. Risk of hepatotoxicity in cancer patients treated with immune checkpoint inhibitors: A systematic review and meta-analysis of published data. Int J Cancer. 2017;141(5):1018-1028. doi:10.1002/ijc.30678
398. Yang W, Li S, Yang Q. Risk of dermatologic and mucosal adverse events associated with PD-1/PD-L1 inhibitors in cancer patients: A meta-analysis of randomized controlled trials. Medicine (Baltimore). 2019;98(20):e15731. doi:10.1097/MD.0000000000015731
399. Lv WW, Zhang JJ, Zhou XL, Song Z, Wei CM. Safety of combining vascular endothelial growth factor receptor tyrosine-kinase inhibitors with chemotherapy in patients with advanced non-small-cell lung cancer: A PRISMA-compliant meta-analysis. Medicine (Baltimore). 2019;98(23):e15806. doi:10.1097/MD.0000000000015806
400. Sun W, Li J. Skin Toxicities with Epidermal Growth Factor Receptor Tyrosine Kinase Inhibitors in Cancer Patients: A Meta-Analysis of Randomized Controlled Trials. Cancer Invest. 2019;37(6):253-264. doi:10.1080/07357907.2019.1634089
401. Luo W, Wang Z, Tian P, Li W. Safety and tolerability of PD-1/PD-L1 inhibitors in the treatment of non-small cell lung cancer: a meta-analysis of randomized controlled trials. J Cancer Res Clin Oncol. 2018;144(10):1851-1859. doi:10.1007/s00432-018-2707-4
402. Liang XJ, Shen J. Adverse events risk associated with angiogenesis inhibitors addition to therapy in ovarian cancer: a meta-analysis of randomized controlled trials. Eur Rev Med Pharmacol Sci. 2016;20(12):2701-2709.
403. Zhang X, Ran Y, Shao Y, Wang K, Zhu Y. Incidence and risk of severe infections associated with aflibercept in cancer patients: a systematic review and meta-analysis. Br J Clin Pharmacol. 2016;81(1):33-40. doi:10.1111/bcp.12758
404. Zhang X, Ran Y, Wang K, Zhu Y, Li J. Incidence and risk of hepatic toxicities with PD-1 inhibitors in cancer patients: a meta-analysis. Drug Des Devel Ther. 2016;10:3153-3161. Published 2016 Sep 28. doi:10.2147/DDDT.S115493
405. Zhang X, Shao Y, Wang K. Incidence and risk of hypertension associated with cabozantinib in cancer patients: a systematic review and meta-analysis. Expert Rev Clin Pharmacol. 2016;9(8):1109-1115. doi:10.1080/17512433.2016.1190269
406. Guo X, Yang Q, Dong J, Liao L, Zhang W, Liu F. Tumour Risk with Once-Weekly Glucagon-Like Peptide-1 Receptor Agonists in Type 2 Diabetes Mellitus Patients: A Systematic Review. Clin Drug Investig. 2016;36(6):433-441. doi:10.1007/s40261-016-0389-8
407. Jing X, Li Y, Xu J. Risk of Cardiovascular Events Associated with Inhaled Corticosteroid Treatment in Patients with Chronic Obstructive Pulmonary Disease: A Meta-Analysis. Can Respir J. 2018;2018:7097540. Published 2018 Jul 15. doi:10.1155/2018/7097540
408. Zhao X, Wu X, Dong J, Liu Y, Zheng L, Zhang L. A Meta-analysis of Postoperative Complications of Tissue Expander/Implant Breast Reconstruction Using Acellular Dermal Matrix. Aesthetic Plast Surg. 2015;39(6):892-901. doi:10.1007/s00266-015-0555-z
409. Zhuang XD, He X, Yang DY, et al. Comparative cardiovascular outcomes in the era of novel anti-diabetic agents: a comprehensive network meta-analysis of 166,371 participants from 170 randomized controlled trials. Cardiovasc Diabetol. 2018;17(1):79. Published 2018 Jun 5. doi:10.1186/s12933-018-0722-z
410. Wang XF, Huang WF, Nie J, Zhou Y, Tan DW, Jiang JH. Toxicity of chemotherapy regimens in advanced and metastatic pancreatic cancer therapy: A network meta-analysis. J Cell Biochem. 2018;119(7):5082-5103. doi:10.1002/jcb.26266
411. Li X, Wan J, Wu Z, et al. Fatal adverse events with molecular targeted agents in the treatment of advanced hepatocellular carcinoma: a meta-analysis of randomized controlled trials. Drug Des Devel Ther. 2018;12:3043-3049. Published 2018 Sep 18. doi:10.2147/DDDT.S151241
412. Shi XH, Zhou X, Zhang YM, Lei ZY, Liu T, Fan DL. Complications from Nasolabial Fold Injection of Calcium Hydroxylapatite for Facial Soft-Tissue Augmentation: A Systematic Review and Meta-Analysis. Aesthet Surg J. 2016;36(6):712-717. doi:10.1093/asj/sjv206
413. Zhang XH, Hao S, Gao B, et al. A network meta-analysis for toxicity of eight chemotherapy regimens in the treatment of metastatic/advanced breast cancer. Oncotarget. 2016;7(51):84533-84543. doi:10.18632/oncotarget.13023
414. Zhu X, Wu S. Increased Risk of Hypertension with Enzalutamide in Prostate Cancer: A Meta-Analysis. Cancer Invest. 2019;37(9):478-488. doi:10.1080/07357907.2019.1670203
415. Zhu X, Tian X, Yu C, Hong J, Fang J, Chen H. Increased risk of hemorrhage in metastatic colorectal cancer patients treated with bevacizumab: An updated meta-analysis of 12 randomized controlled trials. Medicine (Baltimore). 2016;95(34):e4232. doi:10.1097/MD.0000000000004232
416. Li X, Huang R, Xu Z. Risk of Adverse Vascular Events in Newly Diagnosed Glioblastoma Multiforme Patients Treated with Bevacizumab: a Systematic Review and Meta-Analysis. Sci Rep. 2015;5:14698. Published 2015 Oct 1. doi:10.1038/srep14698
417. Xu X, Zhu H, Lv H. Safety of Staphylococcus aureus four-antigen and three-antigen vaccines in healthy adults: A meta-analysis of randomized controlled trials. Hum Vaccin Immunother. 2018;14(2):314-321. doi:10.1080/21645515.2017.1395540
418. Feng X, Tian M, Zhang W, Mei H. Gastrointestinal safety of etoricoxib in osteoarthritis and rheumatoid arthritis: A meta-analysis. PLoS One. 2018;13(1):e0190798. Published 2018 Jan 10. doi:10.1371/journal.pone.0190798
419. Pan XB, Huang ST, Jiang YM, Ma JL, Zhu XD. Secondary malignancies after partial versus whole breast irradiation: a systematic review and meta-analysis. Oncotarget. 2016;7(44):71951-71959. doi:10.18632/oncotarget.12442
420. Yang X, Pan X, Cheng X, Cheng Y, Kuang Y. Risk of treatment-related mortality with sorafenib in cancer patients: a meta-analysis of 20 randomly controlled trials : Risk of sorafenib-associated death. Int J Clin Pharm. 2015;37(6):1047-1056. doi:10.1007/s11096-015-0151-y
421. Yang X, Pan X, Cheng X, Kuang Y, Cheng Y. Risk of Gastrointestinal Events During Vandetanib Therapy in Patients With Cancer: A Systematic Review and Meta-analysis of Clinical Trials. Am J Ther. 2017;24(3):e351-e360. doi:10.1097/MJT.0000000000000306
422. Yang X, Pan X, Cheng X, Kuang Y, Cheng Y. Risk of Gastrointestinal Events During Lapatinib Therapy: A Meta-Analysis From 12,402 Patients With Cancer. Am J Ther. 2018;25(4):e412-e422. doi:10.1097/MJT.0000000000000368
423. Xu N, Yu M, Liu X, Sun C, Chen Z, Liu Z. A systematic review of complications in thoracic spine surgery for ossification of the posterior longitudinal ligament. Eur Spine J. 2017;26(7):1803-1809. doi:10.1007/s00586-015-4097-5
424. 薛晓静,何饶丽,李伟兴,辛佳蔚,叶钦勇,陈晓春,潘晓东.非典型抗精神病药治疗痴呆精神行为症状安全性的系统评价[J].中华医学杂志,2018,98(25):2030-2036.
425. Huang Y, Fan H, Li N, Du J. Risk of immune-related pneumonitis for PD1/PD-L1 inhibitors: Systematic review and network meta-analysis. Cancer Med. 2019;8(5):2664-2674. doi:10.1002/cam4.2104
426. Wu Y, Mu Y, Yin L, Wang Z, Liu W, Wan H. Complications in the Management of Acute Achilles Tendon Rupture: A Systematic Review and Network Meta-analysis of 2060 Patients. Am J Sports Med. 2019;47(9):2251-2260. doi:10.1177/0363546518824601
427. Lin YS, Cheng SW, Wang YH, Chen KH, Fang CJ, Chen C. Systematic review with meta-analysis: risk of post-operative complications associated with pre-operative exposure to anti-tumour necrosis factor agents for Crohn's disease. Aliment Pharmacol Ther. 2019;49(8):966-977. doi:10.1111/apt.15184
428. Zhou Y, Lu H, Yang M, Xu C. Adverse drug events associated with ibrutinib for the treatment of elderly patients with chronic lymphocytic leukemia: A systematic review and meta-analysis of randomized trials. Medicine (Baltimore). 2019;98(33):e16915. doi:10.1097/MD.0000000000016915
429. Hu Y, Xu W, Cao F. A meta-analysis of randomized controlled trials: combination of ketamine and propofol versus ketamine alone for procedural sedation and analgesia in children. Intern Emerg Med. 2019;14(7):1159-1165. doi:10.1007/s11739-019-02173-6
430. Zhao Y, Peng H, Li X, et al. Dual antiplatelet therapy after coronary artery bypass surgery: is there an increase in bleeding risk? A meta-analysis. Interact Cardiovasc Thorac Surg. 2018;26(4):573-582. doi:10.1093/icvts/ivx374
431. Shen Y, Jia Y, Zhou J, Ji J, Xun P. Bayesian Network Meta-Analysis for Assessing Adverse Effects of Anti-hepatitis B Drugs. Clin Drug Investig. 2019;39(9):835-846. doi:10.1007/s40261-019-00802-8
432. Xia Y, Zhao J, Cao DS. Safety of Lipoabdominoplasty Versus Abdominoplasty: A Systematic Review and Meta-analysis. Aesthetic Plast Surg. 2019;43(1):167-174. doi:10.1007/s00266-018-1270-3
433. Bai Y, Chen H, Yang Y, et al. Safety of antithrombotic drugs in patients with atrial fibrillation and non-end-stage chronic kidney disease: Meta-analysis and systematic review. Thromb Res. 2016;137:46-52. doi:10.1016/j.thromres.2015.11.020
434. Zhang Y, Ma L. Effect of preoperative angiotensin-converting enzyme inhibitor on the outcome of coronary artery bypass graft surgery. Eur J Cardiothorac Surg. 2015;47(5):788-795. doi:10.1093/ejcts/ezu298
435. Liu YX, Zhang Y, Huang JF, Wang L. Meta-analysis comparing the safety of laparoscopic and open surgical approaches for suspected adnexal mass during the second trimester. Int J Gynaecol Obstet. 2017;136(3):272-279. doi:10.1002/ijgo.12069
436. Yang Y, Liu YH, Sun X, et al. Risk of peripheral edema in cancer patients treated with MEK inhibitors: a systematic review and meta-analysis of clinical trials. Curr Med Res Opin. 2017;33(9):1663-1675. doi:10.1080/03007995.2017.1349657
437. Pan Y, Hu C, Chen PH, et al. Association of oral endothelin receptor antagonists with risks of cardiovascular events and mortality: meta-analysis of randomized controlled trials. Eur J Clin Pharmacol. 2017;73(3):267-278. doi:10.1007/s00228-016-2171-5
438. Liu Y, Qi M, Hou S, et al. Risk of rash associated with vandetanib treatment in non-small-cell lung cancer patients: A meta-analysis of 9 randomized controlled trials. Medicine (Baltimore). 2017;96(43):e8345. doi:10.1097/MD.0000000000008345
439. Xing Y, Chen L, Feng Y, Zhou Y, Zhai Y, Lu J. Meta-analysis of the safety of voriconazole in definitive, empirical, and prophylactic therapies for invasive fungal infections. BMC Infect Dis. 2017;17(1):798. Published 2017 Dec 28. doi:10.1186/s12879-017-2913-8
440. Wang Y, Zhou S, Yang F, et al. Treatment-Related Adverse Events of PD-1 and PD-L1 Inhibitors in Clinical Trials: A Systematic Review and Meta-analysis. JAMA Oncol. 2019;5(7):1008-1019. doi:10.1001/jamaoncol.2019.0393
441. Liu Y, Zhang X, Chai S, Zhao X, Ji L. Risk of Malignant Neoplasia with Glucagon-Like Peptide-1 Receptor Agonist Treatment in Patients with Type 2 Diabetes: A Meta-Analysis. J Diabetes Res. 2019;2019:1534365. Published 2019 Jul 16. doi:10.1155/2019/1534365
442. Zhao YT, Li PY, Zhang JQ, Wang L, Yi Z. Angiotensin II Receptor Blockers and Cancer Risk: A Meta-Analysis of Randomized Controlled Trials. Medicine (Baltimore). 2016;95(18):e3600. doi:10.1097/MD.0000000000003600
443. Geng Z, Yu Y, Hu S, Dong L, Ye C. Tocilizumab and the risk of respiratory adverse events in patients with rheumatoid arthritis: a systematic review and meta-analysis of randomised controlled trials. Clin Exp Rheumatol. 2019;37(2):318-323.
444. Yuan ZZ, Yang Z, Liu Q, Liu YM. Complications following open reduction and internal fixation versus external fixation in treating unstable distal radius fractures: Grading the evidence through a meta-analysis. Orthop Traumatol Surg Res. 2018;104(1):95-103. doi:10.1016/j.otsr.2017.08.020
445. Malihi Z, Wu Z, Stewart AW, Lawes CM, Scragg R. Hypercalcemia, hypercalciuria, and kidney stones in long-term studies of vitamin D supplementation: a systematic review and meta-analysis. Am J Clin Nutr. 2016;104(4):1039-1051. doi:10.3945/ajcn.116.134981
446. Malihi Z, Wu Z, Mm Lawes C, Scragg R. Noncalcemic adverse effects and withdrawals in randomized controlled trials of long-term vitamin D2 or D3 supplementation: a systematic review and meta-analysis. Nutr Rev. 2017;75(12):1007-1034. doi:10.1093/nutrit/nux059
447. Malihi Z, Wu Z, Lawes CMM, Scragg R. Adverse events from large dose vitamin D supplementation taken for one year or longer. J Steroid Biochem Mol Biol. 2019;188:29-37. doi:10.1016/j.jsbmb.2018.12.002
448. Tang Z, Yang Y, Yang Z, Meng W, Li X. Early precut sphincterotomy does not increase the risk of adverse events for patients with difficult biliary access: A systematic review of randomized clinical trials with meta-analysis and trial sequential analysis. Medicine (Baltimore). 2018;97(36):e12213. doi:10.1097/MD.0000000000012213
449. Shen Z, Kong D. Meta-analysis of the adverse events associated with extended-release versus standard immediate-release pramipexole in Parkinson disease. Medicine (Baltimore). 2018;97(34):e11316. doi:10.1097/MD.0000000000011316
450. Jia Z, Lu H, Yang X, et al. Adverse Events of Botulinum Toxin Type A in Facial Rejuvenation: A Systematic Review and Meta-Analysis. Aesthetic Plast Surg. 2016;40(5):769-777. doi:10.1007/s00266-016-0682-1
451. Wu Z, Zhang H, Jin W, et al. The Effect of Renin-Angiotensin-Aldosterone System Blockade Medications on Contrast-Induced Nephropathy in Patients Undergoing Coronary Angiography: A Meta-Analysis. PLoS One. 2015;10(6):e0129747. Published 2015 Jun 17. doi:10.1371/journal.pone.0129747
452. Lv Z, Li Y, Wu Y, Qu Y. Surgical complications of primary rhegmatogenous retinal detachment: a meta-analysis. PLoS One. 2015;10(3):e0116493. Published 2015 Mar 3. doi:10.1371/journal.pone.0116493
453. Wang Z, Yang X, Wang J, et al. Risk of serious adverse event and fatal adverse event with molecular target anticancer drugs in cancer patients: A meta-analysis. J Cancer Res Ther. 2019;15(7):1435-1449. doi:10.4103/jcrt.JCRT_577_18
454. Zhu LN, Chen D, Chen T, Xu D, Chen SH, Liu L. The adverse event profile of brivaracetam: A meta-analysis of randomized controlled trials. Seizure. 2017;45:7-16. doi:10.1016/j.seizure.2016.11.008
455. Jiang Z, Xiao H, Zhang H, Liu S, Meng J. Comparison of adverse events between cluster and conventional immunotherapy for allergic rhinitis patients with or without asthma: A systematic review and meta-analysis. Am J Otolaryngol. 2019;40(6):102269. doi:10.1016/j.amjoto.2019.07.013
456. Tolkien Z, Stecher L, Mander AP, Pereira DI, Powell JJ. Ferrous sulfate supplementation causes significant gastrointestinal side-effects in adults: a systematic review and meta-analysis. PLoS One. 2015;10(2):e0117383. Published 2015 Feb 20. doi:10.1371/journal.pone.0117383

**List of included studies (2008-2011)**

1. Aksoy S, Dizdar O, Hayran M, Harputluoğlu H. Infectious complications of rituximab in patients with lymphoma during maintenance therapy: a systematic review and meta-analysis. Leuk Lymphoma. 2009 Mar;50(3):357-65. doi: 10.1080/10428190902730219. PMID: 19263297.
2. Albavera-Hernández C, Rodríguez JM, Idrovo AJ. Safety of botulinum toxin type A among children with spasticity secondary to cerebral palsy: a systematic review of randomized clinical trials. Clin Rehabil. 2009;23(5):394-407. doi:10.1177/0269215508099860
3. An MM, Zou Z, Shen H, et al. Incidence and risk of significantly raised blood pressure in cancer patients treated with bevacizumab: an updated meta-analysis. Eur J Clin Pharmacol. 2010;66(8):813-821. doi:10.1007/s00228-010-0815-4
4. Arbyn M, Kyrgiou M, Simoens C, et al. Perinatal mortality and other severe adverse pregnancy outcomes associated with treatment of cervical intraepithelial neoplasia: meta-analysis. BMJ. 2008;337:a1284. Published 2008 Sep 18. doi:10.1136/bmj.a1284
5. Bacha OM, Plante M, Kirschnick LS, Edelweiss MI. Evaluation of morbidity of suction drains after retroperitoneal lymphadenectomy in gynecological tumors: a systematic literature review. Int J Gynecol Cancer. 2009;19(2):202-207. doi:10.1111/IGC.0b013e31819a1fbe
6. Bager P, Wohlfahrt J, Westergaard T. Caesarean delivery and risk of atopy and allergic disease: meta-analyses. Clin Exp Allergy. 2008;38(4):634-642. doi:10.1111/j.1365-2222.2008.02939.x
7. Bangalore S, Kumar S, Kjeldsen SE, et al. Antihypertensive drugs and risk of cancer: network meta-analyses and trial sequential analyses of 324,168 participants from randomised trials. Lancet Oncol. 2011;12(1):65-82. doi:10.1016/S1470-2045(10)70260-6
8. Bangalore S, Kumar S, Wetterslev J, Messerli FH. Angiotensin receptor blockers and risk of myocardial infarction: meta-analyses and trial sequential analyses of 147 020 patients from randomised trials. BMJ. 2011;342:d2234. Published 2011 Apr 26. doi:10.1136/bmj.d2234
9. Bar-Oz B, Moretti ME, Boskovic R, O'Brien L, Koren G. The safety of quinolones--a meta-analysis of pregnancy outcomes. Eur J Obstet Gynecol Reprod Biol. 2009;143(2):75-78. doi:10.1016/j.ejogrb.2008.12.007
10. Bennett CL, Silver SM, Djulbegovic B, et al. Venous thromboembolism and mortality associated with recombinant erythropoietin and darbepoetin administration for the treatment of cancer-associated anemia. JAMA. 2008;299(8):914-924. doi:10.1001/jama.299.8.914
11. Bergendal A, Odlind V, Persson I, Kieler H. Limited knowledge on progestogen-only contraception and risk of venous thromboembolism. Acta Obstet Gynecol Scand. 2009;88(3):261-266. doi:10.1080/00016340902730375
12. Bhuriya R, Singh M, Molnar J, Arora R, Khosla S. Bisphosphonate use in women and the risk of atrial fibrillation: a systematic review and meta-analysis. Int J Cardiol. 2010;142(3):213-217. doi:10.1016/j.ijcard.2009.11.041
13. Bloch F, Thibaud M, Dugué B, Brèque C, Rigaud AS, Kemoun G. Laxatives as a risk factor for iatrogenic falls in elderly subjects: myth or reality?. Drugs Aging. 2010;27(11):895-901. doi:10.2165/11584280-000000000-00000
14. Bolland MJ, Avenell A, Baron JA, et al. Effect of calcium supplements on risk of myocardial infarction and cardiovascular events: meta-analysis. BMJ. 2010;341:c3691. Published 2010 Jul 29. doi:10.1136/bmj.c3691
15. Bongartz T, Warren FC, Mines D, Matteson EL, Abrams KR, Sutton AJ. Etanercept therapy in rheumatoid arthritis and the risk of malignancies: a systematic review and individual patient data meta-analysis of randomised controlled trials. Ann Rheum Dis. 2009;68(7):1177-1183. doi:10.1136/ard.2008.094904
16. Brunelli SM. Association between oral sodium phosphate bowel preparations and kidney injury: a systematic review and meta-analysis. Am J Kidney Dis. 2009;53(3):448-456. doi:10.1053/j.ajkd.2008.09.022
17. Canonico M, Plu-Bureau G, Lowe GD, Scarabin PY. Hormone replacement therapy and risk of venous thromboembolism in postmenopausal women: systematic review and meta-analysis. BMJ. 2008;336(7655):1227-1231. doi:10.1136/bmj.39555.441944.BE
18. Cao Y, Liao C, Tan A, Liu L, Gao F. Meta-analysis of incidence and risk of hypomagnesemia with cetuximab for advanced cancer. Chemotherapy. 2010;56(6):459-465. doi:10.1159/000321011
19. Cao Y, Liu L, Liao C, Tan A, Gao F. Meta-analysis of incidence and risk of hypokalemia with cetuximab-based therapy for advanced cancer. Cancer Chemother Pharmacol. 2010;66(1):37-42. doi:10.1007/s00280-009-1131-5
20. Carnes D, Mars TS, Mullinger B, Froud R, Underwood M. Adverse events and manual therapy: a systematic review. Man Ther. 2010;15(4):355-363. doi:10.1016/j.math.2009.12.006
21. Cardwell CR, Stene LC, Joner G, et al. Caesarean section is associated with an increased risk of childhood-onset type 1 diabetes mellitus: a meta-analysis of observational studies. Diabetologia. 2008;51(5):726-735. doi:10.1007/s00125-008-0941-z
22. Castillo JJ, Dalia S, Pascual SK. Association between red blood cell transfusions and development of non-Hodgkin lymphoma: a meta-analysis of observational studies. Blood. 2010;116(16):2897-2907. doi:10.1182/blood-2010-03-276683
23. Chakhtoura Z, Canonico M, Gompel A, Thalabard JC, Scarabin PY, Plu-Bureau G. Progestogen-only contraceptives and the risk of stroke: a meta-analysis. Stroke. 2009;40(4):1059-1062. doi:10.1161/STROKEAHA.108.538405
24. Chan CC, Reid CM, Aw TJ, Liew D, Haas SJ, Krum H. Do COX-2 inhibitors raise blood pressure more than nonselective NSAIDs and placebo? An updated meta-analysis. J Hypertens. 2009;27(12):2332-2341. doi:10.1097/HJH.0b013e3283310dc9
25. Chavez-Tapia NC, Tellez-Avila FI, Bedogni G, Crocè LS, Masutti F, Tiribelli C. Systematic review and meta-analysis on the adverse events of rimonabant treatment: considerations for its potential use in hepatology. BMC Gastroenterol. 2009;9:75. Published 2009 Oct 9. doi:10.1186/1471-230X-9-75
26. Chisholm EJ, Kulinskaya E, Tolley NS. Systematic review and meta-analysis of the adverse effects of thyroidectomy combined with central neck dissection as compared with thyroidectomy alone. Laryngoscope. 2009;119(6):1135-1139. doi:10.1002/lary.20236
27. Choueiri TK, Schutz FA, Je Y, Rosenberg JE, Bellmunt J. Risk of arterial thromboembolic events with sunitinib and sorafenib: a systematic review and meta-analysis of clinical trials. J Clin Oncol. 2010;28(13):2280-2285. doi:10.1200/JCO.2009.27.2757
28. Chu D, Lacouture ME, Fillos T, Wu S. Risk of hand-foot skin reaction with sorafenib: a systematic review and meta-analysis. Acta Oncol. 2008;47(2):176-186. doi:10.1080/02841860701765675
29. Chu D, Lacouture ME, Weiner E, Wu S. Risk of hand-foot skin reaction with the multitargeted kinase inhibitor sunitinib in patients with renal cell and non-renal cell carcinoma: a meta-analysis. Clin Genitourin Cancer. 2009;7(1):11-19. doi:10.3816/CGC.2009.n.002
30. Cleary BJ, Donnelly J, Strawbridge J, et al. Methadone dose and neonatal abstinence syndrome-systematic review and meta-analysis. Addiction. 2010;105(12):2071-2084. doi:10.1111/j.1360-0443.2010.03120.x
31. Cooper RD, Wiebe N, Smith N, Keiser P, Naicker S, Tonelli M. Systematic review and meta-analysis: renal safety of tenofovir disoproxil fumarate in HIV-infected patients. Clin Infect Dis. 2010;51(5):496-505. doi:10.1086/655681
32. Cornish JA, Tan E, Simillis C, Clark SK, Teare J, Tekkis PP. The risk of oral contraceptives in the etiology of inflammatory bowel disease: a meta-analysis. Am J Gastroenterol. 2008;103(9):2394-2400. doi:10.1111/j.1572-0241.2008.02064.x
33. Dhippayom T, Chaiyakunapruk N, Jongchansittho T. Safety of nortriptyline at equivalent therapeutic doses for smoking cessation: a systematic review and meta-analysis. Drug Saf. 2011;34(3):199-210. doi:10.2165/11585950-000000000-00000
34. Dugoua JJ, Machado M, Zhu X, Chen X, Koren G, Einarson TR. Probiotic safety in pregnancy: a systematic review and meta-analysis of randomized controlled trials of Lactobacillus, Bifidobacterium, and Saccharomyces spp. J Obstet Gynaecol Can. 2009;31(6):542-552. doi:10.1016/S1701-2163(16)34218-9
35. Edwards SJ, Smith CJ. Tolerability of atypical antipsychotics in the treatment of adults with schizophrenia or bipolar disorder: a mixed treatment comparison of randomized controlled trials. Clin Ther. 2009;31 Pt 1:1345-1359. doi:10.1016/j.clinthera.2009.07.004
36. Etminan M, Sadatsafavi M, Ganjizadeh Zavareh S, Takkouche B, FitzGerald JM. Inhaled corticosteroids and the risk of fractures in older adults: a systematic review and meta-analysis. Drug Saf. 2008;31(5):409-414. doi:10.2165/00002018-200831050-00005
37. Etminan M, Sadatsafavi M, Jafari S, Doyle-Waters M, Aminzadeh K, FitzGerald JM. Acetaminophen use and the risk of asthma in children and adults: a systematic review and metaanalysis. Chest. 2009;136(5):1316-1323. doi:10.1378/chest.09-0865
38. Eyers S, Weatherall M, Jefferies S, Beasley R. Paracetamol in pregnancy and the risk of wheezing in offspring: a systematic review and meta-analysis. Clin Exp Allergy. 2011;41(4):482-489. doi:10.1111/j.1365-2222.2010.03691.x
39. Fatourechi MM, Kudva YC, Murad MH, Elamin MB, Tabini CC, Montori VM. Clinical review: Hypoglycemia with intensive insulin therapy: a systematic review and meta-analyses of randomized trials of continuous subcutaneous insulin infusion versus multiple daily injections. J Clin Endocrinol Metab. 2009;94(3):729-740. doi:10.1210/jc.2008-1415
40. Fernández-Balsells MM, Murad MH, Lane M, et al. Clinical review 1: Adverse effects of testosterone therapy in adult men: a systematic review and meta-analysis. J Clin Endocrinol Metab. 2010;95(6):2560-2575. doi:10.1210/jc.2009-2575
41. Fernández-Guisasola J, Gómez-Arnau JI, Cabrera Y, del Valle SG. Association between nitrous oxide and the incidence of postoperative nausea and vomiting in adults: a systematic review and meta-analysis. Anaesthesia. 2010;65(4):379-387. doi:10.1111/j.1365-2044.2010.06249.x
42. Ford AC, Malfertheiner P, Giguere M, Santana J, Khan M, Moayyedi P. Adverse events with bismuth salts for Helicobacter pylori eradication: systematic review and meta-analysis. World J Gastroenterol. 2008;14(48):7361-7370. doi:10.3748/wjg.14.7361
43. Gagne JJ, Griesdale DE, Schneeweiss S. Aprotinin and the risk of death and renal dysfunction in patients undergoing cardiac surgery: a meta-analysis of epidemiologic studies. Pharmacoepidemiol Drug Saf. 2009;18(4):259-268. doi:10.1002/pds.1714
44. Gandhi R, Smith H, Lefaivre KA, Davey JR, Mahomed NN. Complications after minimally invasive total knee arthroplasty as compared with traditional incision techniques: a meta-analysis. J Arthroplasty. 2011;26(1):29-35. doi:10.1016/j.arth.2009.11.022
45. García Rodríguez LA, González-Pérez A, Bueno H, Hwa J. NSAID use selectively increases the risk of non-fatal myocardial infarction: a systematic review of randomised trials and observational studies. PLoS One. 2011;6(2):e16780. Published 2011 Feb 8. doi:10.1371/journal.pone.0016780
46. Gartlehner G, Thieda P, Hansen RA, et al. Comparative risk for harms of second-generation antidepressants : a systematic review and meta-analysis. Drug Saf. 2008;31(10):851-865. doi:10.2165/00002018-200831100-00004
47. Gehling M, Tryba M. Risks and side-effects of intrathecal morphine combined with spinal anaesthesia: a meta-analysis. Anaesthesia. 2009;64(6):643-651. doi:10.1111/j.1365-2044.2008.05817.x
48. Gill SK, O'Brien L, Koren G. The safety of histamine 2 (H2) blockers in pregnancy: a meta-analysis. Dig Dis Sci. 2009;54(9):1835-1838. doi:10.1007/s10620-008-0587-1
49. Gill SK, O'Brien L, Einarson TR, Koren G. The safety of proton pump inhibitors (PPIs) in pregnancy: a meta-analysis. Am J Gastroenterol. 2009;104(6):1541-1546. doi:10.1038/ajg.2009.122
50. Glaspy J, Crawford J, Vansteenkiste J, et al. Erythropoiesis-stimulating agents in oncology: a study-level meta-analysis of survival and other safety outcomes. Br J Cancer. 2010;102(2):301-315. doi:10.1038/sj.bjc.6605498
51. Greiser CM, Greiser EM, Dören M. Menopausal hormone therapy and risk of lung cancer-Systematic review and meta-analysis. Maturitas. 2010;65(3):198-204. doi:10.1016/j.maturitas.2009.11.027
52. Grootscholten K, Kok M, Oei SG, Mol BW, van der Post JA. External cephalic version-related risks: a meta-analysis. Obstet Gynecol. 2008;112(5):1143-1151. doi:10.1097/AOG.0b013e31818b4ade
53. Han J, Liu Y. Effect of ventilator circuit changes on ventilator-associated pneumonia: a systematic review and meta-analysis. Respir Care. 2010;55(4):467-474.
54. Hapani S, Chu D, Wu S. Risk of gastrointestinal perforation in patients with cancer treated with bevacizumab: a meta-analysis. Lancet Oncol. 2009;10(6):559-568. doi:10.1016/S1470-2045(09)70112-3
55. Hapani S, Sher A, Chu D, Wu S. Increased risk of serious hemorrhage with bevacizumab in cancer patients: a meta-analysis. Oncology. 2010;79(1-2):27-38. doi:10.1159/000314980
56. Hassan AK, Bergheanu SC, Stijnen T, et al. Late stent malapposition risk is higher after drug-eluting stent compared with bare-metal stent implantation and associates with late stent thrombosis. Eur Heart J. 2010;31(10):1172-1180. doi:10.1093/eurheartj/ehn553
57. Hegeman J, van den Bemt BJ, Duysens J, van Limbeek J. NSAIDs and the risk of accidental falls in the elderly: a systematic review. Drug Saf. 2009;32(6):489-498. doi:10.2165/00002018-200932060-00005
58. Heinemann J, Gillen G, Sanchez-Ramos L, Kaunitz AM. Do mechanical methods of cervical ripening increase infectious morbidity? A systematic review. Am J Obstet Gynecol. 2008;199(2):177-188. doi:10.1016/j.ajog.2008.05.005
59. Heinrich MC, Häberle L, Müller V, Bautz W, Uder M. Nephrotoxicity of iso-osmolar iodixanol compared with nonionic low-osmolar contrast media: meta-analysis of randomized controlled trials. Radiology. 2009;250(1):68-86. doi:10.1148/radiol.2501080833
60. Hohl CM, Kelly-Smith CH, Yeung TC, Sweet DD, Doyle-Waters MM, Schulzer M. The effect of a bolus dose of etomidate on cortisol levels, mortality, and health services utilization: a systematic review. Ann Emerg Med. 2010;56(2):105-13.e5. doi:10.1016/j.annemergmed.2010.01.030
61. Honrubia F, García-Sánchez J, Polo V, de la Casa JM, Soto J. Conjunctival hyperaemia with the use of latanoprost versus other prostaglandin analogues in patients with ocular hypertension or glaucoma: a meta-analysis of randomised clinical trials. Br J Ophthalmol. 2009;93(3):316-321. doi:10.1136/bjo.2007.135111
62. Hu ZY, Yu Q, Zhao YS. Dose-dependent association between UGT1A1*28 polymorphism and irinotecan-induced diarrhoea: a meta-analysis. Eur J Cancer. 2010;46(10):1856-1865. doi:10.1016/j.ejca.2010.02.049
63. Hujoel PP, Zina LG, Moimaz SA, Cunha-Cruz J. Infant formula and enamel fluorosis: a systematic review. J Am Dent Assoc. 2009;140(7):841-854. doi:10.14219/jada.archive.2009.0278
64. Ince H, Valgimigli M, Petzsch M, et al. Cardiovascular events and re-stenosis following administration of G-CSF in acute myocardial infarction: systematic review and meta-analysis. Heart. 2008;94(5):610-616. doi:10.1136/hrt.2006.111385
65. Iodice S, Barile M, Rotmensz N, et al. Oral contraceptive use and breast or ovarian cancer risk in BRCA1/2 carriers: a meta-analysis. Eur J Cancer. 2010;46(12):2275-2284. doi:10.1016/j.ejca.2010.04.018
66. Grzelewski T, Stelmach I. Exercise-induced bronchoconstriction in asthmatic children: a comparative systematic review of the available treatment options. Drugs. 2009;69(12):1533-1553. doi:10.2165/11316720-000000000-00000
67. Jamula E, Anderson J, Douketis JD. Safety of continuing warfarin therapy during cataract surgery: a systematic review and meta-analysis. Thromb Res. 2009;124(3):292-299. doi:10.1016/j.thromres.2009.01.007
68. Je Y, Schutz FA, Choueiri TK. Risk of bleeding with vascular endothelial growth factor receptor tyrosine-kinase inhibitors sunitinib and sorafenib: a systematic review and meta-analysis of clinical trials. Lancet Oncol. 2009;10(10):967-974. doi:10.1016/S1470-2045(09)70222-0
69. Jia Y, Lacouture ME, Su X, Wu S. Risk of skin rash associated with erlotinib in cancer patients: a meta-analysis. J Support Oncol. 2009;7(6):211-217.
70. Johansson K, Neovius K, DeSantis SM, Rössner S, Neovius M. Discontinuation due to adverse events in randomized trials of orlistat, sibutramine and rimonabant: a meta-analysis [published correction appears in Obes Rev. 2009 Sep;10(5):586]. Obes Rev. 2009;10(5):564-575. doi:10.1111/j.1467-789X.2009.00581.x
71. Johnstone J, Nerenberg K, Loeb M. Meta-analysis: proton pump inhibitor use and the risk of community-acquired pneumonia. Aliment Pharmacol Ther. 2010;31(11):1165-1177. doi:10.1111/j.1365-2036.2010.04284.x
72. Jolly SS, Amlani S, Hamon M, Yusuf S, Mehta SR. Radial versus femoral access for coronary angiography or intervention and the impact on major bleeding and ischemic events: a systematic review and meta-analysis of randomized trials. Am Heart J. 2009;157(1):132-140. doi:10.1016/j.ahj.2008.08.023
73. Karamchandani D, Arias-Amaya R, Donaldson N, Gilbert J, Schulte KM. Thyroid cancer and renal transplantation: a meta-analysis. Endocr Relat Cancer. 2010;17(1):159-167. Published 2010 Feb 18. doi:10.1677/ERC-09-0191
74. Khan K, Zamora J, Lamont RF, et al. Safety concerns for the use of calcium channel blockers in pregnancy for the treatment of spontaneous preterm labour and hypertension: a systematic review and meta-regression analysis. J Matern Fetal Neonatal Med. 2010;23(9):1030-1038. doi:10.3109/14767050903572182
75. Khawaja OA, Shaikh KA, Al-Mallah MH. Meta-analysis of adverse cardiovascular events associated with echocardiographic contrast agents. Am J Cardiol. 2010;106(5):742-747. doi:10.1016/j.amjcard.2010.04.034
76. Kim SY, Kim MJ, Cadarette SM, Solomon DH. Bisphosphonates and risk of atrial fibrillation: a meta-analysis. Arthritis Res Ther. 2010;12(1):R30. doi:10.1186/ar2938
77. Knight SR, Morris PJ. Steroid avoidance or withdrawal after renal transplantation increases the risk of acute rejection but decreases cardiovascular risk. A meta-analysis. Transplantation. 2010;89(1):1-14. doi:10.1097/TP.0b013e3181c518cc
78. Krasopoulos G, Brister SJ, Beattie WS, Buchanan MR. Aspirin "resistance" and risk of cardiovascular morbidity: systematic review and meta-analysis. BMJ. 2008;336(7637):195-198. doi:10.1136/bmj.39430.529549.BE
79. Kulisevsky J, Pagonabarraga J. Tolerability and safety of ropinirole versus other dopamine agonists and levodopa in the treatment of Parkinson's disease: meta-analysis of randomized controlled trials. Drug Saf. 2010;33(2):147-161. doi:10.2165/11319860-000000000-00000
80. Kuratani N, Oi Y. Greater incidence of emergence agitation in children after sevoflurane anesthesia as compared with halothane: a meta-analysis of randomized controlled trials. Anesthesiology. 2008;109(2):225-232. doi:10.1097/ALN.0b013e31817f5c18
81. Kwok CS, Loke YK. Meta-analysis: the effects of proton pump inhibitors on cardiovascular events and mortality in patients receiving clopidogrel [published correction appears in Aliment Pharmacol Ther. 2010 Sep;32(5):696]. Aliment Pharmacol Ther. 2010;31(8):810-823. doi:10.1111/j.1365-2036.2010.04247.x
82. Lakhdar R, Al-Mallah MH, Lanfear DE. Safety and tolerability of angiotensin-converting enzyme inhibitor versus the combination of angiotensin-converting enzyme inhibitor and angiotensin receptor blocker in patients with left ventricular dysfunction: a systematic review and meta-analysis of randomized controlled trials. J Card Fail. 2008;14(3):181-188. doi:10.1016/j.cardfail.2007.11.008
83. Leombruno JP, Einarson TR, Keystone EC. The safety of anti-tumour necrosis factor treatments in rheumatoid arthritis: meta and exposure-adjusted pooled analyses of serious adverse events. Ann Rheum Dis. 2009;68(7):1136-1145. doi:10.1136/ard.2008.091025
84. Loke YK, Jeevanantham V, Singh S. Bisphosphonates and atrial fibrillation: systematic review and meta-analysis. Drug Saf. 2009;32(3):219-228. doi:10.2165/00002018-200932030-00004
85. Loke YK, Singh S, Furberg CD. Long-term use of thiazolidinediones and fractures in type 2 diabetes: a meta-analysis. CMAJ. 2009;180(1):32-39. doi:10.1503/cmaj.080486
86. Loke YK, Kwok CS, Singh S. Risk of myocardial infarction and cardiovascular death associated with inhaled corticosteroids in COPD. Eur Respir J. 2010;35(5):1003-1021. doi:10.1183/09031936.00095909
87. Loke YK, Kwok CS, Singh S. Comparative cardiovascular effects of thiazolidinediones: systematic review and meta-analysis of observational studies. BMJ. 2011;342:d1309. Published 2011 Mar 17. doi:10.1136/bmj.d1309
88. Luykx J, Mason M, Ferrari MD, Carpay J. Are migraineurs at increased risk of adverse drug responses? A meta-analytic comparison of topiramate-related adverse drug reactions in epilepsy and migraine. Clin Pharmacol Ther. 2009;85(3):283-288. doi:10.1038/clpt.2008.203
89. Ma MH, Cope AP, Scott DL. Safety of combination therapies in early rheumatoid arthritis: a systematic comparison between antirheumatic drugs and TNF inhibitors with methotrexate. International Journal of Clinical Rheumatology 2010; 5(5): 547-554.
90. Mahmud SM, Franco EL, Aprikian AG. Use of nonsteroidal anti-inflammatory drugs and prostate cancer risk: a meta-analysis. Int J Cancer. 2010;127(7):1680-1691. doi:10.1002/ijc.25186
91. Makarounas-Kirchmann K, Glover-Koudounas S, Ferrari P. Results of a meta-analysis comparing the tolerability of lercanidipine and other dihydropyridine calcium channel blockers [published correction appears in Clin Ther. 2010 Feb;32(2):401-2]. Clin Ther. 2009;31(8):1652-1663. doi:10.1016/j.clinthera.2009.08.010
92. Mangiapane S, Blettner M, Schlattmann P. Aspirin use and breast cancer risk: a meta-analysis and meta-regression of observational studies from 2001 to 2005. Pharmacoepidemiol Drug Saf. 2008;17(2):115-124. doi:10.1002/pds.1503
93. Mannucci E, Monami M, Lamanna C, Gensini GF, Marchionni N. Pioglitazone and cardiovascular risk. A comprehensive meta-analysis of randomized clinical trials. Diabetes Obes Metab. 2008;10(12):1221-1238. doi:10.1111/j.1463-1326.2008.00892.x
94. Mannucci E, Monami M, Di Bari M, et al. Cardiac safety profile of rosiglitazone: a comprehensive meta-analysis of randomized clinical trials. Int J Cardiol. 2010;143(2):135-140. doi:10.1016/j.ijcard.2009.01.064
95. Massó González EL, Patrignani P, Tacconelli S, García Rodríguez LA. Variability among nonsteroidal antiinflammatory drugs in risk of upper gastrointestinal bleeding. Arthritis Rheum. 2010;62(6):1592-1601. doi:10.1002/art.27412
96. Mauri D, Valachis A, Polyzos IP, et al. Osteonecrosis of the jaw and use of bisphosphonates in adjuvant breast cancer treatment: a metanalysis. Breast Cancer Res Treat 116, 433–439 (2009). https://doi.org/10.1007/s10549-009-0432-z
97. McDonald SD, Han Z, Mulla S, et al. Preterm birth and low birth weight among in vitro fertilization twins: a systematic review and meta-analyses. Eur J Obstet Gynecol Reprod Biol. 2010;148(2):105-113. doi:10.1016/j.ejogrb.2009.09.019
98. McDonald SD, Han Z, Mulla S, et al. Preterm birth and low birth weight among in vitro fertilization singletons: a systematic review and meta-analyses. Eur J Obstet Gynecol Reprod Biol. 2009;146(2):138-148. doi:10.1016/j.ejogrb.2009.05.035
99. Miller ER 3rd, Juraschek S, Pastor-Barriuso R, Bazzano LA, Appel LJ, Guallar E. Meta-analysis of folic acid supplementation trials on risk of cardiovascular disease and risk interaction with baseline homocysteine levels. Am J Cardiol. 2010;106(4):517-527. doi:10.1016/j.amjcard.2010.03.064
100. Mills EJ, Wu P, Lockhart I, Wilson K, Ebbert JO. Adverse events associated with nicotine replacement therapy (NRT) for smoking cessation. A systematic review and meta-analysis of one hundred and twenty studies involving 177,390 individuals. Tob Induc Dis. 2010;8(1):8. Published 2010 Jul 13. doi:10.1186/1617-9625-8-8
101. Monami M, Lamanna C, Marchionni N, Mannucci E. Rosiglitazone and risk of cancer: a meta-analysis of randomized clinical trials. Diabetes Care. 2008;31(7):1455-1460. doi:10.2337/dc07-2308
102. Nagajothi N, Adigopula S, Balamuthusamy S, et al. Pioglitazone and the risk of myocardial infarction and other major adverse cardiac events: a meta-analysis of randomized, controlled trials. Am J Ther. 2008;15(6):506-511. doi:10.1097/MJT.0b013e318167180c
103. Nalluri SR, Chu D, Keresztes R, Zhu X, Wu S. Risk of venous thromboembolism with the angiogenesis inhibitor bevacizumab in cancer patients: a meta-analysis. JAMA. 2008;300(19):2277-2285. doi:10.1001/jama.2008.656
104. Natanson C, Kern SJ, Lurie P, Banks SM, Wolfe SM. Cell-free hemoglobin-based blood substitutes and risk of myocardial infarction and death: a meta-analysis [published correction appears in JAMA. 2008 Sep 17;300(11): 1300]. JAMA. 2008;299(19):2304-2312. doi:10.1001/jama.299.19.jrv80007
105. Novara G, Galfano A, Boscolo-Berto R, et al. Complication rates of tension-free midurethral slings in the treatment of female stress urinary incontinence: a systematic review and meta-analysis of randomized controlled trials comparing tension-free midurethral tapes to other surgical procedures and different devices. Eur Urol. 2008;53(2):288-308. doi:10.1016/j.eururo.2007.10.073
106. O'Brien L, Einarson TR, Sarkar M, Einarson A, Koren G. Does paroxetine cause cardiac malformations?. J Obstet Gynaecol Can. 2008;30(8):696-701. doi:10.1016/S1701-2163(16)32918-8
107. Ormerod S, McDowell SE, Coleman JJ, Ferner RE. Ethnic differences in the risks of adverse reactions to drugs used in the treatment of psychoses and depression: a systematic review and meta-analysis. Drug Saf. 2008;31(7):597-607. doi:10.2165/00002018-200831070-00005
108. Palmer SC, Navaneethan SD, Craig JC, et al. Meta-analysis: erythropoiesis-stimulating agents in patients with chronic kidney disease. Ann Intern Med. 2010;153(1):23-33. doi:10.7326/0003-4819-153-1-201007060-00252
109. Palomba S, Falbo A, Orio F Jr, Zullo F. Effect of preconceptional metformin on abortion risk in polycystic ovary syndrome: a systematic review and meta-analysis of randomized controlled trials. Fertil Steril. 2009;92(5):1646-1658. doi:10.1016/j.fertnstert.2008.08.087
110. Papatheodoridis GV, Lampertico P, Manolakopoulos S, Lok A. Incidence of hepatocellular carcinoma in chronic hepatitis B patients receiving nucleos(t)ide therapy: a systematic review. J Hepatol. 2010;53(2):348-356. doi:10.1016/j.jhep.2010.02.035
111. Pascual J, Galeano C, Royuela A, Zamora J. A systematic review on steroid withdrawal between 3 and 6 months after kidney transplantation. Transplantation. 2010;90(4):343-349. doi:10.1097/TP.0b013e3181e58912
112. Petrov MS, van Santvoort HC, Besselink MG, van der Heijden GJ, Windsor JA, Gooszen HG. Enteral nutrition and the risk of mortality and infectious complications in patients with severe acute pancreatitis: a meta-analysis of randomized trials. Arch Surg. 2008;143(11):1111-1117. doi:10.1001/archsurg.143.11.1111
113. Petrov MS, Whelan K. Comparison of complications attributable to enteral and parenteral nutrition in predicted severe acute pancreatitis: a systematic review and meta-analysis. Br J Nutr. 2010;103(9):1287-1295. doi:10.1017/S0007114510000887
114. Polyzos NP, Mauri D, Tsioras S, Messini CI, Valachis A, Messinis IE. Intraperitoneal dissemination of endometrial cancer cells after hysteroscopy: a systematic review and meta-analysis. Int J Gynecol Cancer. 2010;20(2):261-267. doi:10.1111/igc.0b013e3181ca2290
115. Poynten IM, Millwood IY, Falster MO, et al. The safety of candidate vaginal microbicides since nonoxynol-9: a systematic review of published studies. AIDS. 2009;23(10):1245-1254. doi:10.1097/QAD.0b013e32832b4271
116. Rahimi R, Nikfar S, Rezaie A, Abdollahi M. Pregnancy outcome in women with inflammatory bowel disease following exposure to 5-aminosalicylic acid drugs: a meta-analysis. Reprod Toxicol. 2008;25(2):271-275. doi:10.1016/j.reprotox.2007.11.010
117. Rajpathak SN, Kumbhani DJ, Crandall J, Barzilai N, Alderman M, Ridker PM. Statin therapy and risk of developing type 2 diabetes: a meta-analysis. Diabetes Care. 2009;32(10):1924-1929. doi:10.2337/dc09-0738
118. Ramritu P, Halton K, Cook D, Whitby M, Graves N. Catheter-related bloodstream infections in intensive care units: a systematic review with meta-analysis. J Adv Nurs. 2008;62(1):3-21. doi:10.1111/j.1365-2648.2007.04564.x
119. Ranpura V, Hapani S, Chuang J, Wu S. Risk of cardiac ischemia and arterial thromboembolic events with the angiogenesis inhibitor bevacizumab in cancer patients: a meta-analysis of randomized controlled trials. Acta Oncol. 2010;49(3):287-297. doi:10.3109/02841860903524396
120. Ranpura V, Pulipati B, Chu D, Zhu X, Wu S. Increased risk of high-grade hypertension with bevacizumab in cancer patients: a meta-analysis. Am J Hypertens. 2010;23(5):460-468. doi:10.1038/ajh.2010.25
121. Ranpura V, Hapani S, Wu S. Treatment-related mortality with bevacizumab in cancer patients: a meta-analysis [published correction appears in JAMA. 2011 Jun 8;305(22):2294]. JAMA. 2011;305(5):487-494. doi:10.1001/jama.2011.51
122. Rao AD, Kuhadiya N, Reynolds K, Fonseca VA. Is the combination of sulfonylureas and metformin associated with an increased risk of cardiovascular disease or all-cause mortality?: a meta-analysis of observational studies. Diabetes Care. 2008;31(8):1672-1678. doi:10.2337/dc08-0167
123. Ravindran V, Rachapalli S, Choy EH. Safety of medium- to long-term glucocorticoid therapy in rheumatoid arthritis: a meta-analysis. Rheumatology (Oxford). 2009;48(7):807-811. doi:10.1093/rheumatology/kep096
124. Reed M, Meier P, Tamhane UU, Welch KB, Moscucci M, Gurm HS. The relative renal safety of iodixanol compared with low-osmolar contrast media: a meta-analysis of randomized controlled trials [published correction appears in JACC Cardiovasc Interv. 2009 Nov;2(11):1167]. JACC Cardiovasc Interv. 2009;2(7):645-654. doi:10.1016/j.jcin.2009.05.002
125. Rizvi AZ, Murad MH, Fairman RM, Erwin PJ, Montori VM. The effect of left subclavian artery coverage on morbidity and mortality in patients undergoing endovascular thoracic aortic interventions: a systematic review and meta-analysis. J Vasc Surg. 2009;50(5):1159-1169. doi:10.1016/j.jvs.2009.09.002
126. Robert M, Soraisham A, Sauve R. Postoperative urinary incontinence after total abdominal hysterectomy or supracervical hysterectomy: a metaanalysis. Am J Obstet Gynecol. 2008;198(3):264.e1-264.e2645. doi:10.1016/j.ajog.2007.09.033
127. Rodrigo GJ, Nannini LJ, Rodríguez-Roisin R. Safety of long-acting beta-agonists in stable COPD: a systematic review. Chest. 2008;133(5):1079-1087. doi:10.1378/chest.07-1167
128. Rodrigo GJ, Moral VP, Marcos LG, Castro-Rodriguez JA. Safety of regular use of long-acting beta agonists as monotherapy or added to inhaled corticosteroids in asthma. A systematic review. Pulm Pharmacol Ther. 2009;22(1):9-19. doi:10.1016/j.pupt.2008.10.008
129. Rodrigo GJ, Castro-Rodriguez JA, Nannini LJ, Plaza Moral V, Schiavi EA. Tiotropium and risk for fatal and nonfatal cardiovascular events in patients with chronic obstructive pulmonary disease: systematic review with meta-analysis. Respir Med. 2009;103(10):1421-1429. doi:10.1016/j.rmed.2009.05.020
130. Salliot C, Dougados M, Gossec L. Risk of serious infections during rituximab, abatacept and anakinra treatments for rheumatoid arthritis: meta-analyses of randomised placebo-controlled trials. Ann Rheum Dis. 2009;68(1):25-32. doi:10.1136/ard.2007.083188
131. Samii A, Etminan M, Wiens MO, Jafari S. NSAID use and the risk of Parkinson's disease: systematic review and meta-analysis of observational studies. Drugs Aging. 2009;26(9):769-779. doi:10.2165/11316780-000000000-00000
132. Dören M. Association between hormone replacement therapy and subsequent arterial and venous vascular events: a meta-analysis. Eur Heart J. 2009;30(7):866-867. doi:10.1093/eurheartj/ehn618
133. Satterthwaite TD, Wolf DH, Rosenheck RA, Gur RE, Caroff SN. A meta-analysis of the risk of acute extrapyramidal symptoms with intramuscular antipsychotics for the treatment of agitation. J Clin Psychiatry. 2008;69(12):1869-1879. doi:10.4088/jcp.v69n1204
134. Sattar N, Preiss D, Murray HM, et al. Statins and risk of incident diabetes: a collaborative meta-analysis of randomised statin trials. Lancet. 2010;375(9716):735-742. doi:10.1016/S0140-6736(09)61965-6
135. Sawka AM, Thabane L, Parlea L, et al. Second primary malignancy risk after radioactive iodine treatment for thyroid cancer: a systematic review and meta-analysis. Thyroid. 2009;19(5):451-457. doi:10.1089/thy.2008.0392
136. Schwarz EB, Moretti ME, Nayak S, Koren G. Risk of hypospadias in offspring of women using loratadine during pregnancy: a systematic review and meta-analysis. Drug Saf. 2008;31(9):775-788. doi:10.2165/00002018-200831090-00006
137. Schnaudigel S, Gröschel K, Pilgram SM, Kastrup A. New brain lesions after carotid stenting versus carotid endarterectomy: a systematic review of the literature. Stroke. 2008;39(6):1911-1919. doi:10.1161/STROKEAHA.107.500603
138. Schulze-Rath R, Hammer GP, Blettner M. Are pre- or postnatal diagnostic X-rays a risk factor for childhood cancer? A systematic review. Radiat Environ Biophys. 2008;47(3):301-312. doi:10.1007/s00411-008-0171-2
139. Scott PA, Kingsley GH, Scott DL. Non-steroidal anti-inflammatory drugs and cardiac failure: meta-analyses of observational studies and randomised controlled trials. Eur J Heart Fail. 2008;10(11):1102-1107. doi:10.1016/j.ejheart.2008.07.013
140. Scott AS, Parr LA, Johnstone PA. Risk of cerebrovascular events after neck and supraclavicular radiotherapy: a systematic review. Radiother Oncol. 2009;90(2):163-165. doi:10.1016/j.radonc.2008.12.019
141. Selvin E, Bolen S, Yeh HC, et al. Cardiovascular outcomes in trials of oral diabetes medications: a systematic review. Arch Intern Med. 2008;168(19):2070-2080. doi:10.1001/archinte.168.19.2070
142. Shah PS, Zao J; Knowledge Synthesis Group of Determinants of preterm/LBW births. Induced termination of pregnancy and low birthweight and preterm birth: a systematic review and meta-analyses. BJOG. 2009;116(11):1425-1442. doi:10.1111/j.1471-0528.2009.02278.x
143. Shibata MC, León H, Chatterley T, Dorgan M, Vandermeer B. Do calcium channel blockers increase the diagnosis of heart failure in patients with hypertension?. Am J Cardiol. 2010;106(2):228-235. doi:10.1016/j.amjcard.2010.02.031
144. Sin DD, Tashkin D, Zhang X, et al. Budesonide and the risk of pneumonia: a meta-analysis of individual patient data. Lancet. 2009;374(9691):712-719. doi:10.1016/S0140-6736(09)61250-2
145. Singh S, Loke YK, Furberg CD. Inhaled anticholinergics and risk of major adverse cardiovascular events in patients with chronic obstructive pulmonary disease: a systematic review and meta-analysis [published correction appears in JAMA. 2009 Mar 25;301(12):1227-30]. JAMA. 2008;300(12):1439-1450. doi:10.1001/jama.300.12.1439
146. Singh S, Amin AV, Loke YK. Long-term use of inhaled corticosteroids and the risk of pneumonia in chronic obstructive pulmonary disease: a meta-analysis. Arch Intern Med. 2009;169(3):219-229. doi:10.1001/archinternmed.2008.550
147. Sint Nicolaas J, de Jonge V, Steyerberg EW, Kuipers EJ, van Leerdam ME, Veldhuyzen-van Zanten SJ. Risk of colorectal carcinoma in post-liver transplant patients: a systematic review and meta-analysis. Am J Transplant. 2010;10(4):868-876. doi:10.1111/j.1600-6143.2010.03049.x
148. Slobogean BL, Jackman H, Tennant S, Slobogean GP, Mulpuri K. Iatrogenic ulnar nerve injury after the surgical treatment of displaced supracondylar fractures of the humerus: number needed to harm, a systematic review. J Pediatr Orthop. 2010;30(5):430-436. doi:10.1097/BPO.0b013e3181e00c0d
149. Smith M, Hopkins D, Peveler RC, Holt RI, Woodward M, Ismail K. First- v. second-generation antipsychotics and risk for diabetes in schizophrenia: systematic review and meta-analysis. Br J Psychiatry. 2008;192(6):406-411. doi:10.1192/bjp.bp.107.037184
150. Smith LA, Cornelius VR, Plummer CJ, et al. Cardiotoxicity of anthracycline agents for the treatment of cancer: systematic review and meta-analysis of randomised controlled trials. BMC Cancer. 2010;10:337. Published 2010 Jun 29. doi:10.1186/1471-2407-10-337
151. Solomon SD, Wittes J, Finn PV, et al. Cardiovascular risk of celecoxib in 6 randomized placebo-controlled trials: the cross trial safety analysis. Circulation. 2008;117(16):2104-2113. doi:10.1161/CIRCULATIONAHA.108.764530
152. Southey ER, Soares-Weiser K, Kleijnen J. Systematic review and meta-analysis of the clinical safety and tolerability of ibuprofen compared with paracetamol in paediatric pain and fever. Curr Med Res Opin. 2009;25(9):2207-2222. doi:10.1185/03007990903116255
153. Sipahi I, Debanne SM, Rowland DY, Simon DI, Fang JC. Angiotensin-receptor blockade and risk of cancer: meta-analysis of randomised controlled trials. Lancet Oncol. 2010;11(7):627-636. doi:10.1016/S1470-2045(10)70106-6
154. Sperber K, Hom C, Chao CP, Shapiro D, Ash J. Systematic review of hydroxychloroquine use in pregnant patients with autoimmune diseases. Pediatr Rheumatol Online J. 2009;7:9. Published 2009 May 13. doi:10.1186/1546-0096-7-9
155. Subramanian V, Saxena S, Kang JY, Pollok RC. Preoperative steroid use and risk of postoperative complications in patients with inflammatory bowel disease undergoing abdominal surgery. Am J Gastroenterol. 2008;103(9):2373-2381. doi:10.1111/j.1572-0241.2008.01942.x
156. Sun JC, Whitlock R, Cheng J, et al. The effect of pre-operative aspirin on bleeding, transfusion, myocardial infarction, and mortality in coronary artery bypass surgery: a systematic review of randomized and observational studies. Eur Heart J. 2008;29(8):1057-1071. doi:10.1093/eurheartj/ehn104
157. Tacconelli E, De Angelis G, Cataldo MA, Pozzi E, Cauda R. Does antibiotic exposure increase the risk of methicillin-resistant Staphylococcus aureus (MRSA) isolation? A systematic review and meta-analysis. J Antimicrob Chemother. 2008;61(1):26-38. doi:10.1093/jac/dkm416
158. Takagi H, Matsui M, Umemoto T. Increased late mortality with percutaneous stenting for unprotected left main coronary artery stenosis relative to coronary artery bypass grafting: a meta-analysis of observational studies. J Thorac Cardiovasc Surg. 2010;139(5):1351-1353. doi:10.1016/j.jtcvs.2010.01.024
159. 唐凌峰,姜辉,商学军,赵连明,白泉,洪锴,刘德风,柳建明,袁人培,陈茜,马潞林.输精管结扎术与前列腺癌发病风险关系的Meta分析[J].中华男科学杂志,2009,15(06):545-550.
160. Testa L, Van Gaal WJ, Biondi Zoccai GG, et al. Myocardial infarction after percutaneous coronary intervention: a meta-analysis of troponin elevation applying the new universal definition. QJM. 2009;102(6):369-378. doi:10.1093/qjmed/hcp005
161. Thavagnanam S, Fleming J, Bromley A, Shields MD, Cardwell CR. A meta-analysis of the association between Caesarean section and childhood asthma. Clin Exp Allergy. 2008;38(4):629-633. doi:10.1111/j.1365-2222.2007.02780.x
162. Tondo L, Vázquez G, Baldessarini RJ. Mania associated with antidepressant treatment: comprehensive meta-analytic review. Acta Psychiatr Scand. 2010;121(6):404-414. doi:10.1111/j.1600-0447.2009.01514.x
163. Torloni MR, Vedmedovska N, Merialdi M, et al. Safety of ultrasonography in pregnancy: WHO systematic review of the literature and meta-analysis. Ultrasound Obstet Gynecol. 2009;33(5):599-608. doi:10.1002/uog.6328
164. Touzé E, Trinquart L, Chatellier G, Mas JL. Systematic review of the perioperative risks of stroke or death after carotid angioplasty and stenting [published correction appears in Stroke. 2010 Apr;41(4):e400] [published correction appears in Stroke. 2010 Nov;41(11):e601]. Stroke. 2009;40(12):e683-e693. doi:10.1161/STROKEAHA.109.562041
165. Trelle S, Reichenbach S, Wandel S, et al. Cardiovascular safety of non-steroidal anti-inflammatory drugs: network meta-analysis. BMJ. 2011;342:c7086. Published 2011 Jan 11. doi:10.1136/bmj.c7086
166. Vercellini P, Eskenazi B, Consonni D, et al. Oral contraceptives and risk of endometriosis: a systematic review and meta-analysis. Hum Reprod Update. 2011;17(2):159-170. doi:10.1093/humupd/dmq042
167. Wan Y, Heneghan C, Perera R, et al. Anticoagulation control and prediction of adverse events in patients with atrial fibrillation: a systematic review. Circ Cardiovasc Qual Outcomes. 2008;1(2):84-91. doi:10.1161/CIRCOUTCOMES.108.796185
168. Wang T, Collet JP, Shapiro S, Ware MA. Adverse effects of medical cannabinoids: a systematic review. CMAJ. 2008;178(13):1669-1678. doi:10.1503/cmaj.071178
169. Weatherall M, James K, Clay J, et al. Dose-response relationship for risk of non-vertebral fracture with inhaled corticosteroids. Clin Exp Allergy. 2008;38(9):1451-1458. doi:10.1111/j.1365-2222.2008.03029.x
170. Weatherall M, Clay J, James K, Perrin K, Shirtcliffe P, Beasley R. Dose-response relationship of inhaled corticosteroids and cataracts: a systematic review and meta-analysis. Respirology. 2009;14(7):983-990. doi:10.1111/j.1440-1843.2009.01589.x
171. Woolcott JC, Richardson KJ, Wiens MO, et al. Meta-analysis of the impact of 9 medication classes on falls in elderly persons [published correction appears in Arch Intern Med. 2010 Mar 8;170(5):477]. Arch Intern Med. 2009;169(21):1952-1960. doi:10.1001/archinternmed.2009.357
172. Wu S, Chen JJ, Kudelka A, Lu J, Zhu X. Incidence and risk of hypertension with sorafenib in patients with cancer: a systematic review and meta-analysis. Lancet Oncol. 2008;9(2):117-123. doi:10.1016/S1470-2045(08)70003-2
173. Wu S, Kim C, Baer L, Zhu X. Bevacizumab increases risk for severe proteinuria in cancer patients. J Am Soc Nephrol. 2010;21(8):1381-1389. doi:10.1681/ASN.2010020167
174. Xing KH, Morrison G, Lim W, Douketis J, Odueyungbo A, Crowther M. Has the incidence of deep vein thrombosis in patients undergoing total hip/knee arthroplasty changed over time? A systematic review of randomized controlled trials. Thromb Res. 2008;123(1):24-34. doi:10.1016/j.thromres.2008.05.005
175. Yaghoobi M, Farrokhyar F, Yuan Y, Hunt RH. Is there an increased risk of GERD after Helicobacter pylori eradication?: a meta-analysis. Am J Gastroenterol. 2010;105(5):1007-1014. doi:10.1038/ajg.2009.734
176. Yang P, Zhou Y, Chen B, et al. Aspirin use and the risk of gastric cancer: a meta-analysis. Dig Dis Sci. 2010;55(6):1533-1539. doi:10.1007/s10620-009-0915-0
177. Yu SH, Beirne OR. Laryngeal mask airways have a lower risk of airway complications compared with endotracheal intubation: a systematic review. J Oral Maxillofac Surg. 2010;68(10):2359-2376. doi:10.1016/j.joms.2010.04.017
178. Yu SK, Tait G, Karkouti K, Wijeysundera D, McCluskey S, Beattie WS. The safety of perioperative esmolol: a systematic review and meta-analysis of randomized controlled trials. Anesth Analg. 2011;112(2):267-281. doi:10.1213/ANE.0b013e3182025af7
179. Zagui RM, Matayoshi S, Moura FC. Efeitos adversos associados à aplicação de toxina botulínica na face: revisão sistemática com meta-análise [Adverse effects associated with facial application of botulinum toxin: a systematic review with meta-analysis]. Arq Bras Oftalmol. 2008;71(6):894-901. doi:10.1590/s0004-27492008000600027
180. 张书宁,孙爱军,葛均波,姚康,黄浙勇,王克强,邹云增.经冠状动脉自体骨髓干细胞移植治疗急性心肌梗死安全性的系统评价[J].中华心血管病杂志,2008(08):679-684.
181. 张峰,吴斌,高爽.中国人耻骨上前列腺摘除术与经尿道前列腺电切术对勃起功能及逆行射精影响的Meta分析[J].中华男科学杂志,2009,15(08):738-741.
182. Zhao YS, Zhu S, Li XW, et al. Association between NSAIDs use and breast cancer risk: a systematic review and meta-analysis. Breast Cancer Res Treat. 2009;117(1):141-150. doi:10.1007/s10549-008-0228-6
183. Zhou B, Sun Q, Cong R, et al. Hormone replacement therapy and ovarian cancer risk: a meta-analysis [published correction appears in Gynecol Oncol. 2008 Sep;110(3):455]. Gynecol Oncol. 2008;108(3):641-651. doi: 10.1016/j.ygyno.2007.12.003
184. Zreik TG, Mazloom A, Chen Y, et al. Fertility drugs and the risk of breast cancer: a meta-analysis and review. Breast Cancer Res Treat. 2010;124(1):13-26. doi:10.1007/s10549-010-1140-4

| **1** | **(Sub-) item #** | **Checklist item** | | | | | **Reported**  **on page #** |
| --- | --- | --- | --- | --- | --- | --- | --- |
| **TITLE** | | | | | | |  |
| 1. Title | 1a | Specify the study design with terms such as “overview of (systematic) reviews,” “umbrella review,” “(systematic) review of systematic reviews,” or “(systematic) meta-review” in the title of the OoSRs. | | | | | 1 |
|  | 1b | Mention “safety” or harms related terms, or the adverse event(s) of interest in the title of the OoSRs. | | | | | 1 |
| **ABSTRACT** | | | | | | |  |
| 2. Structured-like summary | 2a | Provide a structured-like abstract, as applicable: background, objective, data sources, selection criteria, data extraction, review appraisal, data synthesis methods, results, limitations, conclusions. | | | | | 2 |
|  | 2b | Report the main findings of analysis of harms undertaken in the OoSRs or/and in the included SRs. | | | | | 2 |
| **INTRODUCTION** | | | | | | |  |
| 3. Rationale | 3a | Specify the rationale and the scope (wide or narrow agendas) for the overview in the context of an existing body of knowledge on the topic. | | | | | 3 |
|  | 3b | Provide a balanced presentation of potential benefits and harms of the intervention(s). | | | | | 3 |
|  | 3c**^a^** | Define which events are considered harms according to previous literature and provide a clear rationale for the specific harms included in the OoSRs. | | | | | 5 |
| 4. Objectives  (PICOS) | 4 | Provide an explicit statement of research question(s) that specifies PICOS: | | | | | 4 |
|  |  | \| 4 \| \| --- \|  - Participants | \| 4 \| \| --- \|  - Interventions | \| 4 \| \| --- \|  - Comparators | \| 4 \| \| --- \|  - Outcomes | \| 4 \| \| --- \|  - Study design |  |
| **METHODS** | | | | | | |  |
| 5. Protocol and registration | 5a | Indicate clearly if a protocol exists or not. | | | | | 5, and supplementary file |
|  | 5b | If registered, provide the name of the registry (such as a valid Web address, PROSPERO). | | | | | Not registered |
| 6. Eligibility criteria  & outcomes of interest | 6a | Specify inclusion and exclusion criteria for study design, participants, interventions and comparators in detail. | | | | | 5 |
|  | 6b | List (and define whenever it is necessary) the outcomes for which data were recorded, ideally include prioritization of main and additional outcomes. | | | | | 5-6 |
|  | 6c | Include adverse events as (primary or secondary) outcome of interest. Define them and grade their severity (such as mild, moderate, severe, fatal; severity could also be described in the appendix), if appropriate. | | | | | 5 |
|  | 6d**^b^** | Specify report characteristics (such as language restrictions, publication status, and years considered) used as criteria for eligibility for the OoSRs (see also item 7). | | | | | 5 |
| 7. Information sources | 7a | Search at least two electronic bases. | | | | | 4-5 |
|  | 7b | Search supplementary sources (e.g. hand-searching, reference lists, related reviews and guidelines, protocol registries, conference abstracts, and other gray literature). | | | | | 4-5 |
|  | 7c | Report the date last searched and/or dates of coverage for each database. | | | | | 4 |
| 8. Search strategy**^c^** | 8a | Specify full electronic search strategy (algorithm) for at least one database including any limits used (e.g. language and date restrictions-see also subitems 6d and 7c) such that it could be repeated. | | | | | Supplementary |
|  | 8b | Present any additional search process (e.g. algorithm or filter for adverse events, searches in pertinent websites) specifically to identify adverse events that have been investigated. | | | | | 5 |
| 9. Data management &  selection process | 9a**^d^** | Describe the software that was used to manage records and data throughout the OoSRs. | | | | | 5 |
|  | 9b | Define what is a SR and provide the process for selecting SRs and its relevant details (screening the title and abstract or full text by at least two reviewers, selection by multiple independent investigators and resolving disagreements by consensus). | | | | | 5 |
|  | 9c | Report any attempt to handle overlapping (include one review among multiple potential candidates by choosing for example the most updated SR, the most methodologically rigorous SR or the SR with larger number of primary studies). | | | | | NA |
| 10. Additional search for  primary studies | 10 | Report additional search to identify eligible primary studies (e.g. searching in more databases or update the search) and its relevant details. | | | | | NA |
| 11. Data collection process | 11a | Describe the method of data extraction from included SRs (e.g. data collection form, extraction in duplicate and independently, resolving disagreements by consensus). | | | | | 5-6 |
|  | 11b | Report any processes for obtaining, confirming or updating data from investigators (e.g. contact with authors of included reviews, obtain data from primary studies of included reviews). | | | | | NA |
| 12. Data items | 12 | List (and define whenever is necessary) the specific variables for which data were recorded (e.g. PICOS items, number of included studies and participants, dose, length of follow up, results, funding sources) and any data assumptions and simplifications made. | | | | | 5-6 |
| 13. Assessment of methodological quality & quality of evidence | 13a | State the evaluation of reporting or/and methodological quality (eg. using PRISMA or PRISMA-harms, AMSTAR or R-AMSTAR) of the included reviews. | | | | | NA |
|  | 13b**^e^** | State the evaluation of quality for individual studies that were included in the SRs (inform whether tools such as Jadad or RoB of Cochrane were used by the included reviews) and for the additional primary studies. | | | | | NA |
|  | 13c | State the evaluation of quality of evidence (e.g. using GRADE approach). | | | | | NA |
|  | 13d | Describe the methods (e.g. piloted forms, independently, in duplicate) used for the quality assessment. | | | | | NA |
| 14. Meta-bias(es) | 14 | Specify any planned assessment of meta-bias(es) (such as publication bias or selective reporting across studies, ROBIS tool). | | | | | NA |
| 15. Data synthesis | 15a | Specify clearly the method (narrative, meta-analysis or network meta-analysis) of handling or synthesizing data and their details (e.g. state the principal summary measures that were extracted or calculated, how heterogeneity was assessed, what statistical approaches were used if a quantitative synthesis has been conducted). | | | | | 6-7 |
|  | 15b | Describe the software that was used to analyze the data if a quantitative synthesis has been conducted. | | | | | 6-7 |
|  | 15c | Report if zero events are included in the studies and how they were handled in statistical analyses, if relevant. | | | | | 6-7 |
|  | 15d | Describe methods of any pre-specified additional analyses (such as sensitivity or subgroup analyses, meta-regression). | | | | | 6-7 |
| **RESULTS** | | | | | | |  |
| 16. Review & primary study selection | 16a | Provide the details of review selection (e.g. numbers of reviews screened, retrieved, and included and excluded in the overview) and the number of the additional eligible primary studies that were included, ideally with a flow diagram of the overview process. | | | | | 7-8 |
|  | 16b | Present a flow diagram that gives separately the number of studies focused on harms outcomes. | | | | | Figure 1 |
|  | 16c**^c^** | List the studies (full citation) that were excluded after reading the full text and provide reasons. | | | | | NA |
| 17. Review & primary study characteristics | 17a**^c^** | Describe characteristics of each included SR in tables (such as title or author, search date, PICOS, design and number of studies included, number and age range of participants, dose/frequency, follow up period [treatment duration], review limitations, results or conclusion) and of each additional primary study. | | | | | 7-8, Table 1, 2 |
|  | 17b | For each included SR report language and publication status restrictions that have been used. | | | | | 7-8 |
| 18. Overlapping | 18 | Present or/and discuss about overlapping of studies within SRs (at least one of the following): | | | | | NA |
|  |  | - Present measures of overlap (such as CCA). | | | | | NA |
|  |  | - Provide citation matrix.**^c^** | | | | | NA |
|  |  | - Give the number of index publications or/and discuss about overlapping.**^f^** | | | | | NA |
| 19. Present assessment of methodological quality & quality of evidence | 19 | Present results in text or/and tables**^c^** of any quality assessment (see also subitems 13a-c): | | | | | NA |
|  |  | - Reporting or/and methodological quality of the included SRs. | | | | | NA |
|  |  | - Inform for the quality of the individual studies that were included in the SRs (report results for sequence generation, allocation concealment, blinding, withdrawals, bias etc.) and for the additional included primary studies. | | | | | NA |
|  |  | - Quality of evidence. | | | | | NA |
| 20. Present meta-bias(es) | 20 | Present results of any assessment of meta-bias(es) (such as publication bias or selective reporting across studies, ROBIS assessment). | | | | | NA |
| 21. Synthesis of results | 21a | Summarize and present the main findings of the overview for benefits and harms. If a quantitative synthesis has been conducted, present each summary measure with a confidence interval, prediction interval or a credible interval and measures of heterogeneity or inconsistency. | | | | | 8-10 |
|  | 21b | Give results of any additional analyses (such as sensitivity, subgroup analyses, or meta-regression). | | | | | 8-10 |
|  | 21c | Report results for adverse events separately for each intervention. | | | | | 8-10 |
| **DISCUSSION** | | | | | | |  |
| 22. Summary of evidence | 22 | Provide a concise summary of the main findings with the strength and shortcomings of evidence for each main outcome. | | | | | 11-12 |
| 23. Limitations | 23a | Discuss limitations of either the overview or included studies (or both) (e.g. different eligibility criteria, limitations of searching reviews, language restrictions, publication and selection bias). | | | | | 13-14 |
|  | 23b | Report possible limitations of the included reviews related to harms (issues of missing data and information, definitions of harms, rare adverse effects). | | | | | 13-14 |
| 24. Conclusions | 24a | Provide a general interpretation of the results in coherence with the review findings and present implications for practice; consider the harms equally as carefully as the benefits and in the context of other evidence. | | | | | 13 |
|  | 24b | Present implications for future research. | | | | | 14 |
| **AUTHORSHIP** | | | | | | |  |
| 25. Contributions of authors | 25 | Provide contributions of authors. | | | | | 15 |
| 26. Dual (co-)authorship | 26 | Report about dual (co-)authorship in the limitation or declarations of interest section. | | | | | 15 |
| **FUNDING** | | | | | | |  |
| 27. Funding or other support | 27a | Indicate sources of financial and other support for the OoSRs (direct funding) or for the authors (indirect funding), or report no funding. | | | | | 15 |
|  | 27b | Provide name for the overview funder and/or sponsor, or for the authors’ supporters. | | | | | 15 |
|  | 27c | Describe roles of funder(s), sponsor(s), and/or institution(s), if any, in conducted the OoSRs. | | | | | 15 |

**Figure S1.** Comparison of the proportions of discarding zero-events studies for the present to the past.


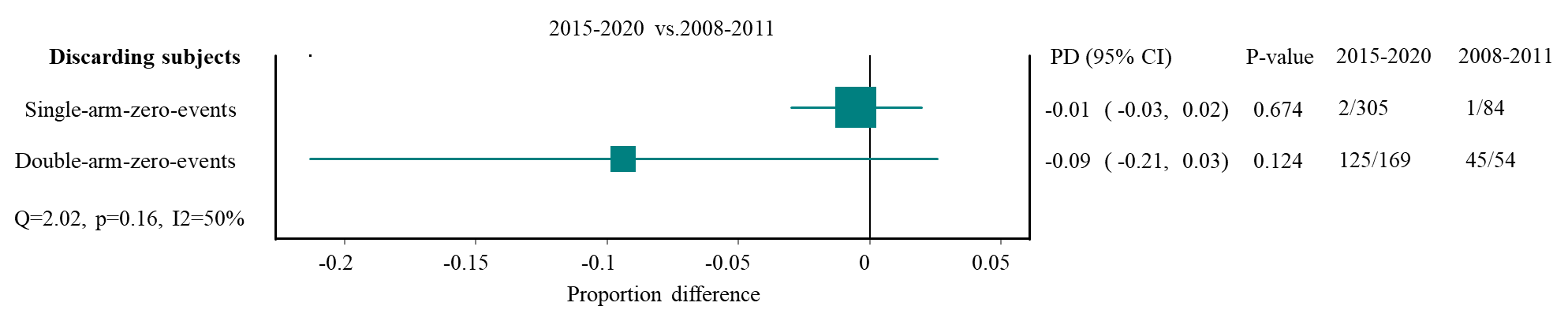


**Figure S2.** Comparison of the proportions of discarding zero-events studies in terms of protocol development or not.


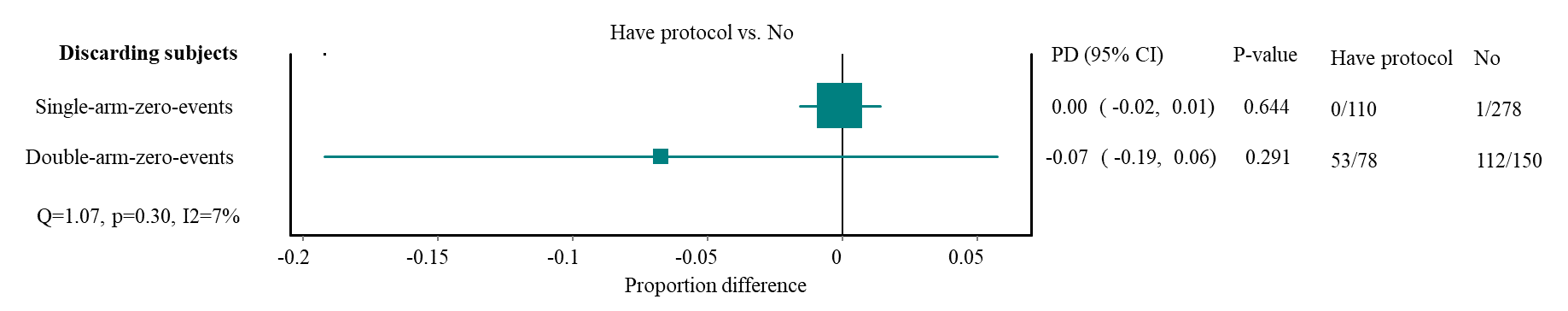


**Figure S3. The framework to guide evidence-synthesis for meta-analysis with zero-events studies**

**
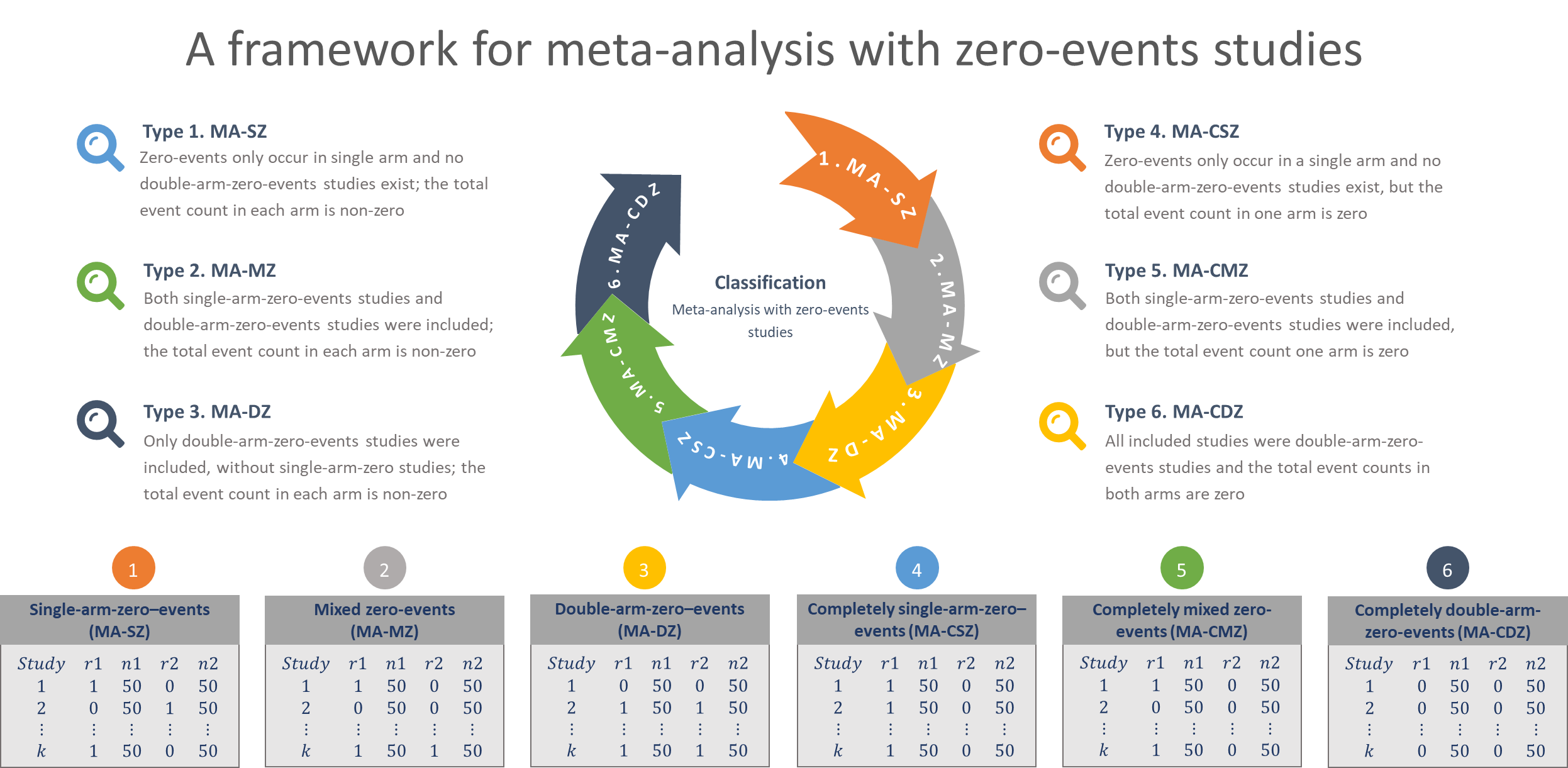
**

**(**Figure is from: *Xu C, Furuya-Kanamori L, Zorzela L, Lin L, Vohra S. A proposed framework to guide evidence synthesis practice for meta-analysis with zero-events studies. 2021;135:70-78.***)**

**Six subtypes of meta-analysis with zero-events studies:**

1) meta-analysis contains zero-events only occurring in single arms, no double-arm-zero-events studies are included, and the total events count in neither arm is zero (MA-SZ);

2) meta-analysis has a mixture of both single-arm-zero-events and double-arm-zero-events studies, and the total events count in neither arm is zero (MA-MZ);

3) meta-analysis only includes double-arm-zero-events studies, and the total events count in neither arm is zero (MA-DZ);

4) meta-analysis contains zero-events occurring in single arms, and no double-arm-zero-events studies are included, while the events count in *one of the arms* is completely zero in all studies (MA-CSZ);

5) meta-analysis has a mixture of both single-arm-zero-events and double-arm-zero-events studies, and the events count in *one of the arms* is completely zero in all studies (MA-CMZ);

6) meta-analysis only includes double-arm-zero-events studies, and the events count *in both arms* is completely zero in all studies (MA-CDZ)

**Figure S4. The flow diagram to guide synthesis practice under the proposed framework**

**
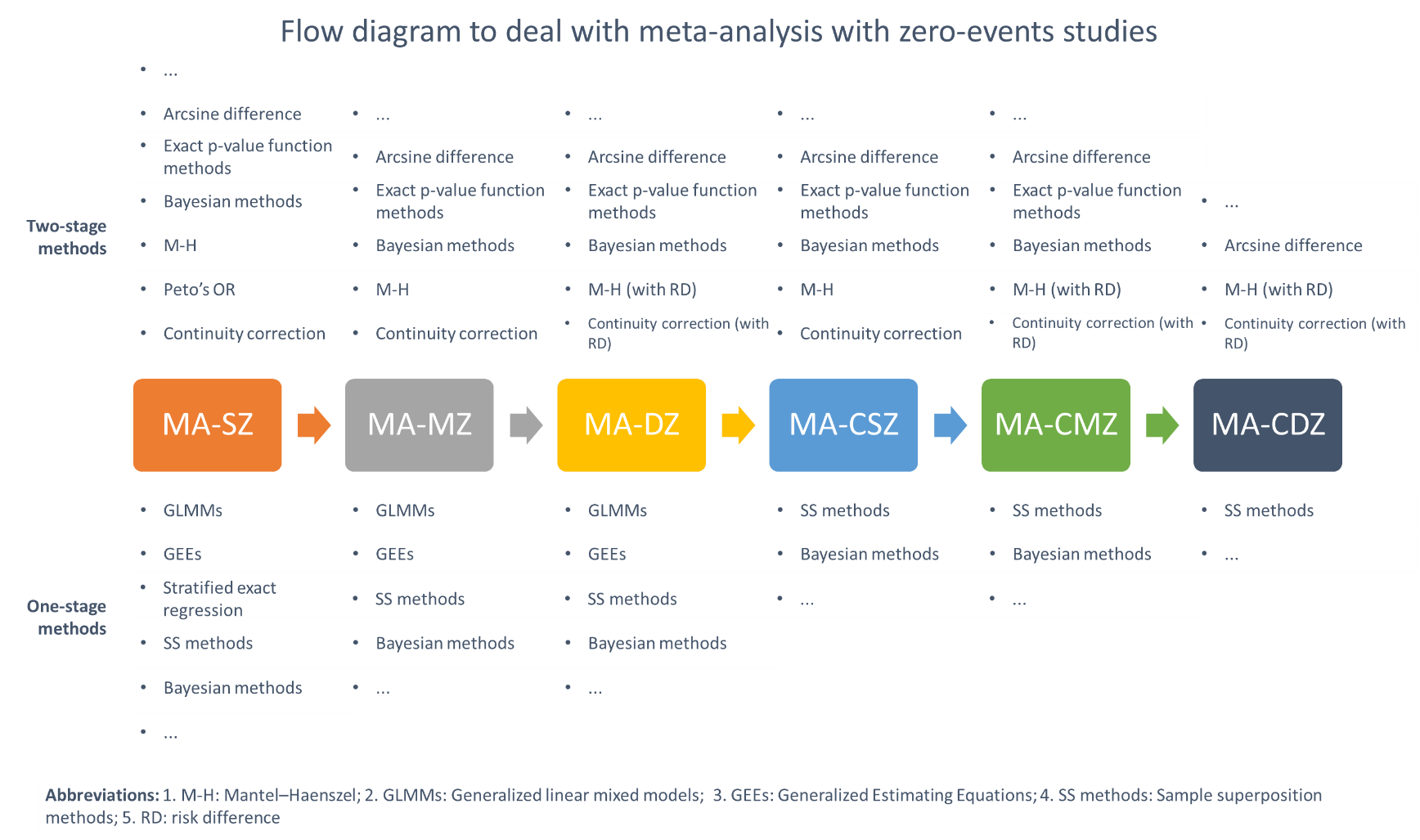
**

**(**Figure is from: *Xu C, Furuya-Kanamori L, Zorzela L, Lin L, Vohra S. A proposed framework to guide evidence synthesis practice for meta-analysis with zero-events studies. 2021;135:70-78.***)**
